# The rhizome of *Reclinomonas americana*, *Homo sapiens*, *Pediculus humanus *and *Saccharomyces cerevisiae *mitochondria

**DOI:** 10.1186/1745-6150-6-55

**Published:** 2011-10-20

**Authors:** Kalliopi Georgiades, Didier Raoult

**Affiliations:** 1 Unité de Recherche en Maladies Infectieuses Tropical Emergentes (URMITE) CNRS-IRD UMR 6236-198, Université de la Méditerranée, Faculté de Médecine La Timone, 27, Bd Jean Moulin, 13385, Marseille cedex 5, France

## Abstract

**Background:**

Mitochondria are thought to have evolved from eubacteria-like endosymbionts; however, the origin of the mitochondrion remains a subject of debate. In this study, we investigated the phenomenon of chimerism in mitochondria to shed light on the origin of these organelles by determining which species played a role in their formation. We used the mitochondria of four distinct organisms, *Reclinomonas americana*, *Homo sapiens*, *Saccharomyces cerevisiae *and multichromosome *Pediculus humanus*, and attempted to identify the origin of each mitochondrial gene.

**Results:**

Our results suggest that the origin of mitochondrial genes is not limited to the *Rickettsiales *and that the creation of these genes did not occur in a single event, but through multiple successive events. Some of these events are very old and were followed by events that are more recent and occurred through the addition of elements originating from current species. The points in time that the elements were added and the parental species of each gene in the mitochondrial genome are different to the individual species. These data constitute strong evidence that mitochondria do not have a single common ancestor but likely have numerous ancestors, including proto-*Rickettsiales*, proto-*Rhizobiales *and proto-*Alphaproteobacteria*, as well as current alphaproteobacterial species. The analysis of the multichromosome *P. humanus *mitochondrion supports this mechanism.

**Conclusions:**

The most plausible scenario of the origin of the mitochondrion is that ancestors of *Rickettsiales *and *Rhizobiales *merged in a proto-eukaryotic cell approximately one billion years ago. The fusion of the *Rickettsiales *and *Rhizobiales *cells was followed by gene loss, genomic rearrangements and the addition of alphaproteobacterial elements through ancient and more recent recombination events. Each gene of each of the four studied mitochondria has a different origin, while in some cases, multichromosomes may allow for enhanced gene exchange. Therefore, the tree of life is not sufficient to explain the chimeric structure of current genomes, and the theory of a single common ancestor and a top-down tree does not reflect our current state of knowledge. Mitochondrial evolution constitutes a rhizome, and it should be represented as such.

**Reviewers:**

This article was revised by William Martin, Arcady Mushegian and Eugene V. Koonin.

## Background

Mitochondria are thought to have evolved from eubacteria-like endosymbionts [1]. The origin of the mitochondrion has been widely studied but remains a subject of debate. In general, ancestors of the *Alphaproteobacteria *subgroup are thought to be the progenitors of mitochondria [2,3]. Indeed, molecular phylogenomic analyses of whole mitochondrial proteins rooted mitochondria among the *Alphaproteobacteria *[4-6]. However, the identity of the organism most related to eukaryotic mitochondria and the placement of the mitochondrial tree branch are contested [7], even though it has been argued that the closest relatives to mitochondria are organisms in the order of *Rickettsiales *[8]. Nevertheless, the relationship of mitochondria to *Rickettsiales *has been challenged based on phylogenomic studies that have demonstrated a close relationship of mitochondria to *Rhodospirillum rubrum *[4]. Furthermore, other studies have linked mitochondria to *Rhizobiales *and *Rhodobacterales *[6]. Finally, a recent study demonstrated the significant role of other bacteria outside the order of *Rickettsiales *in the formation of mitochondria and the occurrence of genome chimerism [9]. All of this evidence allows us to consider the possibility that mitochondria of different organisms may not have originated from the same ancestor and that mitochondria may contain elements with different origins.

In our study, we wished to investigate the phenomenon of chimerism in mitochondria to shed light on the origin of these organelles by determining which species played a role in mitochondria formation. We used the mitochondria of four distinct organisms, *Reclinomonas americana*, *Homo sapiens*, *Saccharomyces cerevisiae and Pediculus humanus*, and attempted to identify the origin of each gene. Then, with the obtained results, we attempted to reconstruct the genealogical tree of the four studied types of mitochondria, which led us to a reconstruction of the mitochondria rhizome.

## Results

### Phylogenetic analyses and mosaic structure

In every phylogenetic gene tree for each of the four types of mitochondria, we searched for the mitochondrion's sister taxa and classified the sister taxa in categories according to the bootstrap values obtained. For the *Reclinomonas americana *mitochondrion, four genes have a group of *Rickettsiales *as a sister taxon, with a bootstrap value greater than 95. Four other genes also have *Rickettsiales *as a sister taxon, but with bootstrap values less than 95. Only one gene has *Rhizobiales *as a sister taxon, with a bootstrap value equal to 100, whereas six other genes with *Rhizobiales *as a sister taxon have bootstrap values less than 95. Finally, eight genes have other *Alphaproteobacteria *as sister taxa, with bootstrap values varying from 94 to 22 (Table 1). In conclusion, 25% of the *Reclinomonas americana *mitochondrial genes have *Rickettsiales *as a sister taxon, 50% of which have a bootstrap value greater than 95, and 21.8% of the genes have *Rhizobiales *as a sister taxon, with only one bootstrap value greater than 95 (see additional file 1: *Reclinomonas americana *mitochondrial phylogenies).

**Table 1:** Sister taxa of *Reclinomonas americana *mitochondrion genes.

Sister taxon Rickettsiales/Rhizobiales bootstrap > 95		
Genes	Sister taxon	Bootstrap
Ribosomal protein L2	Rickettsia prowazekii/Rhizobiales	99
RNA polymerase beta	Rickettsiales	100
RNA polymerase beta'	Rickettsiales (Ehrlichia canis; E. ruminantium)	99
Succinate ubiquinone oxidoreductase 2	Rickettsiales (Ehrlichia canis)	100
ATP transporter ATP binding	Rhizobiales (Mesorhizobium opportunistum; M. loti)	100

**Sister taxon Rickettsiales/Rhizobiales bootstrap < 95**		
**Genes**	**Sister taxon**	**Bootstrap**

Ribosomal protein L20	Rickettsiales	65
Ribosomal protein L27	Rickettsiales (Ehrlichia species)	32
Ribosomal protein L14	Rickettsiales (Rickettsia prowazekii, R. typhi, Pelagibacter ubique)	21
TatC translocase	Rickettsiales (Pelagibacter ubique)	21
Ribosomal protein S1	Rhizobiales	52
Ribosomal protein S4	Rhizobiales (Starkeya novella)	46
Ribosomal protein S14	Rhizobiales	38
NADH deshydrogenase subunit 1	Rhizobiales (O. anthropi; R. rubrum; R. palustris)	57
NADH deshydrogenase subunit 8	Rhizobiales (O. anthropi; R. rubrum; R. palustris; R. sphaeroides)	88
Haem biosynthesis	Rhizobiales (O. anthropi; R. rubrum; R. palustris; R. sphaeroides)	38

**Sister taxon other Alphaproteobacteria**		
**Genes**	**Sister taxon**	**Bootstrap**

Ribosomal protein S2	Group Alphaproteobacteria	94
Ribosomal protein S7	Group Alphaproteobacteria	69
ABC transporter C subunit	Group Alphaproteobacteria	40
Ribosomal protein L11	Group Alphaproteobacteria	11
Ribosomal protein L6	Betaproteobacteria	47
Elongation factor Tu	Granulibacter bethesdensis	41
Ribosomal protein L16	Parvularcula bermudensis	38
Ribosomal protein S19	Maricaulis maris	22

For the *H. sapiens *mitochondrion, one gene was found to cluster with *Rickettsiales*, *Ehrlichia canis *and *E. chaffeensis*, with a bootstrap value equal to 90, and two other genes were identified to be sister taxa of *Pelagibacter ubique *(bootstrap value < 90). Eight other genes clustered with *Rhizobiales *species (bootstrap values < 90) (Table 2). Finally, 72% of the *H. sapiens *mitochondrial genes were found to be related to *Rhizobiales *genes (see additional file 2: *Homo sapiens *mitochondrial phylogenies).

**Table 2:** Sister taxa of the *Homo sapiens *mitochondrion genes.

Sister taxon Rickettsiales		
Genes	Sister taxon	Bootstrap
NADH deshydrogenase subunit 6	Rickettsiales (Ehrlichia canis/E. chaffeensis)	90
Cytochrome c oxidase I	Pelagibacter ubique	53
NADH deshydrogenase subunit 3	Pelagibacter ubique	23

**Sister taxon Rhizobiales**		
**Genes**	**Sister taxon**	**Bootstrap**

NADH deshydrogenase subunit 1	Group Rhizobiales (Rhodospirillales)	92
NADH deshydrogenase subunit 4	Group Rhizobiales	88
ATP synthase F0 subunit 6	Beijerinckia indica	65
ATP synthase F0 subunit 8	Bradyrhizobium sp.	40
NADH deshydrogenase subunit 4L	Rhodospirillum centenum	37
Cytochrome c oxidase II	Group Rhizobiales	36
Cytochrome c oxidase III	Azospirillum sp./R. bellii/R. canadensis	17
NADH deshydrogenase subunit 2	Mitochondrion outgroup of Rhizobiales	-

For the *Saccharomyces cerevisiae *mitochondrion, five genes have *Rickettsiales *species as a sister taxon (bootstrap value > 95), and two have *Rhizobiales *as a sister taxon, one *Ochrobactrum anthropi *and *Brucella *species and one *Azorhizobium caulinodans *(bootstrap value = 95). Twenty-nine other genes have bootstrap values less than 90 on the node of the mitochondrion with its sister taxa, of which 12 are *Rickettsiales *and 18 are *Rhizobiales*. Finally, six other genes have other *Alphaproteobacteria *as sister taxa (bootstrap value < 90) (Table 3). To summarize, 46% of the *S. cerevisiae *mitochondrial gene trees present *Rhizobiales *as a mitochondrial sister taxon, of which 11% have a bootstrap value greater than 95, and 43.5% present *Rickettsiales *as a mitochondrial sister taxon, with 29% having a bootstrap value greater than 95 (see additional file 3: Saccharomyces cerevisiae mitochondrial phylogenies).

**Table 3:** Sister taxa of *Saccharomyces cerevisiae *mitochondrion genes.

Sister taxon Rickettsiales/Rhizobiales bootstrap > 95		
Genes	Sister taxon	Bootstrap
Cytochrome beta	group Rickettsiales	99
MutS	Rickettsia bellii	99
Glutamyl-tRNA amidotransferase	Orientia tsutsugamushi	99
tRNA-delta isopentepyrophosphatetransferase	Pelagibacter ubique/Alphaproteobacteria	99
Maturase	group Rickettsiales/Parvibaculum lavamentivorans	98
Tyrosyl-tRNA synthetase	Ochrobactrum anthropi/Brucella species	99
Helicase	Azorhizobium caulinodans/Xanthobacter autotrophicus	99

**Sister taxon Rickettsiales/Rhizobiales bootstrap < 90**		
**Genes**	**Sister taxon**	**Bootstrap**

Ubiquinol cytochrome C	group Rickettsiales	86
Elongation factor G	group Rickettsiales	72
Ribosomal protein L2	Wolbachia endosymbiont of Drosophila	72
Seryl-tRNA synthetase	Ehrlichia canis/Wolbachia/Orientia species	71
Cysteine desulfurase	Rickettsia bellii/Magnetospirillum magneticum	67
Prolyl-tRNA synthetase	group Rickettsiales (Anaplasma spp.)	52
Thioredoxin reductase	Pelagibacter ubique	42
Endonuclease	Pelagibacter ubique/Rhodopseudomonas palustris/Azorhizobium caulinodans/Xanthobacter autotrophicus	37
Pyruvate deshydrogenase	Anaplasma phagocytophilium	30
Cytochrome beta	Anaplasma phagocytophilium	29
Ribosomal protein L3	Rickettsia felis	18
Arginyl-tRNA synthetase	Rhodobacter sphaeroides	88
Ribosomal protein S2	Bartonella tribocorum	85
Elongation factor Tu	Sinorhizobium japonicum	82
Ribosomal protein S12	Rhodospirillum rubrum	80
DnaK	group Rhizobiales (dont R. palustris)	71
Succinyl-CoA synthetase	Azospirillum sp.	66
Acetyl-CoA carboxylase	Rhodospirillum rubrum	59
DnaK	Sinorhizobium meliloti/Sinorhizobium medicae/Mesorhizobium loti	49
Enoyl-CoA hydratase	Bartonella henselae/grahamii/tribocorum	49
DEAD box	Rhodospirillum rubrum	48
Oli1p	Rhodospirillum rubrum	40
Glutathione oxidoreductase	Rhodospirillum centeum/Magnetospirillum magneticum	35
GroEL	Rhizobium etli	35
Formate tetrahydrofolate ligase	group Methylobacteria	30
Dihydrolipoamide	Rhodobacter sphaeroides/Rhodobacter capsulatus	16

**Sister taxon other Alphaproteobacteria bootstrap < 90**		
**Genes**	**Sister taxon**	**Bootstrap**

Fumarate hydratase	Ruegeria sp.	82
Deshydrogenase	Geobacter sp./Acetobacter sp.	81
Reverse transcriptase	Sphingomonas wittichii/Novosphingobium aromaticivorans	46
Tryptophanyl-tRNA synthetase	Ruegeria pomeroyi	30
5-aminolevulinate	Gluconobacter oxydans	26
Sco1	Caulobacter crescentus; C.segnis/Beijerinckia indica	23
Aconitate hydratase	Gammaproteobacteria	60

For *Pediculus humanus*, three genes have *Rickettsiales *species as sister taxa (bootstrap value > 95), 11 genes also have *Rickettsiales *as sister taxa, but with bootstrap values less than 95, whereas four other genes have *Rhizobiales *as sister taxa (bootstrap value < 95). Finally, eight genes have other Alphaproteobacteria as sister taxa (Table 4). In short, 51.75% of the *P. humanus *mitochondrial gene trees present *Rickettsiales *as a mitochondrion sister taxon, of which 11% have bootstrap values greater than 95, and 14.8% have *Rhizobiales *as a sister taxon (see additional file 4: *Pediculus humanus *mitochondrial phylogenies).

**Table 4:** Sister taxa of *Pediculus humanus *mitochondrion genes

Sister taxon Rickettsiales/Rhizobiales bootstrap > 95		
Genes	Sister taxon	Bootstrap
Cytochrome oxidase beta	Rickettsia canadensis; R. akari; R. rickettsii	99

Ribosomal protein S5	Pelagibacter ubique	99

Cox3	Rickettsiales (Ehrlichia canis; Neorickettsia sennetsu; Neorickettsia risticii)	95

**Sister taxon Rickettsiales/Rhizobiales bootstrap < 95**		
**Genes**	**Sister taxon**	**Bootstrap**

Nad4	N. sennetsu/N. risticii	93
tRNA-tyrosine	Pelagibacter ubique	91
Cox1	Pelagibacter ubique	90
NADH deshydrogenase 3	Rickettsiae	89
Nad2	Rhodobacter sphaeroides	89
Ribosomal protein L22	Orientia tsutsugamushi	75
Ribosomal protein L19	Ehrlichia spp.	73
Ribosomal protein S2	Wolbachia spp.	66
Ribosomal protein S16	Wolbachia spp.	61
Ribosomal protein S14	Wolbachia spp.	55
ATP synthase	Rhizobium leguminosarum	54
Nad3	Rhodopsirillum centenum/Azospirillum sp.	54
ATP synthase F0	Rickettsiales (Neorickettsia, Wolbachia, Anaplasma, Ehrlichia)	51
Cytochrome oxidase 2	Wolbachia endosymbionts of Drosophila and Culex	48
Ribosomal protein L13	Liberibacter asiaticus	44

**Sister taxon other Alphaproteobacteria**		
**Genes**	**Sister taxon**	**Bootstrap**

Intermediate peptidase	Pseudomonas aeruginosa	99
Nad5	Gluconacetobacter diazotrophicus	77
Nd1	Magnetospirillum magneticum	75
Ribosomal protein L24	Hirschia baltica	66
Ribosomal protein L17	Geobacter sulfureducens	61
Ribosomal protein L27	Nitrobacter	46
Cytochrome oxidase su 1	Xanthobacter autotrophicus	40
tRNA-glycine	Acidiphilium cryptum	32

**Figure 1:**
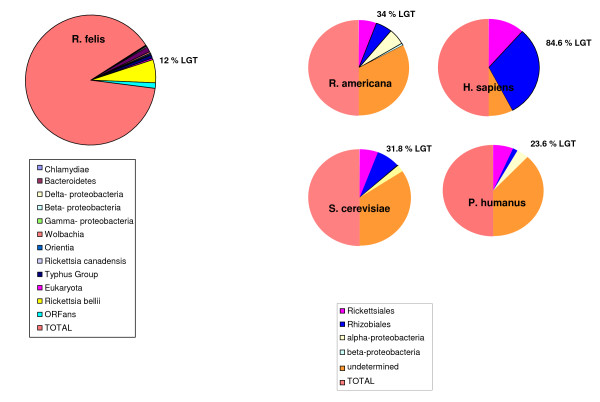
**Percentage of LGT in *Rickettsia felis *and mitochondrial genomes**. The different origins of genes are represented by different colored circles.

**Figure 2:**
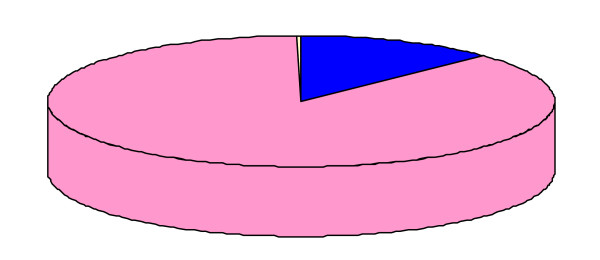
***Rickettsia *species pan-genome**. The core genome (701 genes) is represented in blue, LGT (22 genes) in yellow and unique genes (4726 genes) in pink.

For the four analyzed types of mitochondria, not all genes gave interpretable BLAST matches or phylogenies; in some cases, the BLAST search gave hits with very low e-values and coverage percentages, or the topologies were too difficult to analyze. We therefore focused on the most robust results to draw conclusions. The use of phylogenies to identify horizontal transfers has been demonstrated to cause interpretation problems [10,11]. Furthermore, Alphaproteobacteria are thought to be mosaics, and they are known to have undergone multiple gene transfers [12]. We therefore checked for such ancestral transfers in the species found as mitochondrial sister taxa, and we found only two cases of previous gene transfers: the ribosomal protein L6 of *R. americana *was gained by Betaproteobacteria, whereas the aconitate hydratase protein *of S. cerevisiae *was gained by Gammaproteobacteria (see additional file 5: Previous horizontal gene transfers in Alphaproteoabacteria). However, none of the other phylogenies demonstrated any ancestral transfers. The proportion of mosaicism in ancestors such as *rickettsial *species is statistically inferior to the proportion of mosaicism in *R. americana, H. sapiens and S. cerevisiae *mitochondrial genomes (p = 0.049; p < 0.0001; and p = 0.073, respectively) (Figure 1). Moreover, the pan-genome of *Rickettsia *species is mostly composed of specific genes, and lateral gene transfer (LGT) events occupy a very small place in the pan-genome (Figure 2). Therefore, all of the identified transfers in this study took place directly in the mitochondrial genomes.

**Figure 3:**
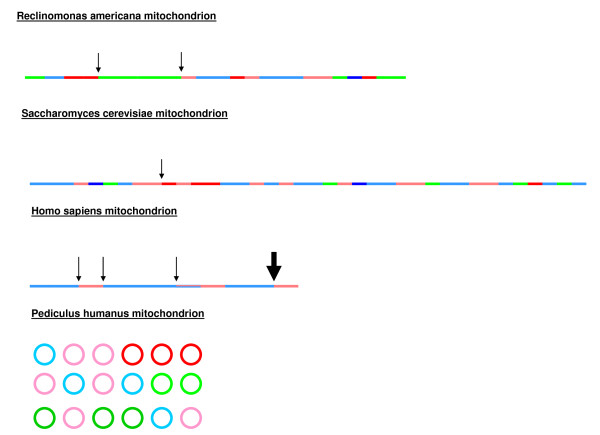
**Mosaic structure of the four studied types of mitochondria**. Each colored line represents a gene. Colors vary according to the original species (red and pink for *Rickettsiales*; blue and sky-blue for *Rhizobiales*). Dark colors represent stable topologies. Arrows show the positions of repeat elements.

Using the evidence from these phylogenies, we represented the resulting mosaic structure of each of the four types of mitochondria (Figure 3). Each gene is represented with a colored line according to its origin. This visualization demonstrates the mosaic structure of mitochondria, which seem to be mostly composed of *Rickettsiales *and *Rhizobiales *genes, whereas some alphaproteobacterial elements appear to have been added more recently in the evolutionary time scale. However, this structure is not stable, and topologies were not always robust. Therefore, the question emerges of whether mitochondria are the outcome of genomic fusions or recombination events.

### Recombination events

Hotspots of repeat elements may constitute regions of genomic recombination events. Therefore, we first looked for possible repeat elements in the genomes of the four types of mitochondria. In the *R. americana *mitochondrion, we found very few repeat elements around an *Alphaproteobacteria*-originated gene region. In the human mitochondrion, we identified four hotspots of repeat elements, whereas in the *S. cerevisiae *mitochondrion, we only identified one repeat element (Figure 3). The *P. humanus *genome is still incomplete, so we were not able to use it to identify repeat elements. Next, we used the Recombination Analysis Tool (RAT) to identify possible recombination events in the genomes of the four types of mitochondria. The software did not reveal any recombination events for *H. sapiens *and *S. cerevisiae *mitochondria. In contrast, for *R. americana*, we identified four genes obtained *via *recombination events. The ribosomal protein L16 recombined with a sequence from *Parvularcula bermudensis*, a marine alphaproteobacterium; the ribosomal proteins L14 and L27 recombined with sequences from *Pelagibacter ubique *of the order of *Rickettsiales*; and finally, succinate ubiquinone oxidoreductase recombined with the sequence of another member of the *Rickettsiales *order, *Ehrlichia canis *(see additional file 6: Recombination events). There is synteny conservation between the regions around the ribosomal L16 proteins of *P. bermudensis *and the mitochondrion. In the mitochondrial genome, the ribosomal protein L16 is found in a region that contains four other ribosomal proteins (L2, S19, S3 and L14). These proteins are also found in the *P. bermudensis *genome near the L16 ribosomal protein sequence and are in the same order. Similar synteny conservation is also observed for the ribosomal protein L14 of the mitochondrion and of *P. ubique*. The L14 sequence is found near the L16, L5 and S14 ribosomal proteins in the mitochondrion, which are also found in the region close to the ribosomal protein L14 in *P. ubique*. The ribosomal proteins L14 and L16 are found on each side of a repeat element in the mitochondrial genome. The locations of the ribosomal protein L27 and succinate ubiquinone oxidoreductase, however, do not demonstrate synteny conservation either with *P*. ubique or E. canis. For *P. humanus*, the program identified four recombination events: the ribosomal protein L22 recombined with *Orientia tsutsugamushi *Ikeda, the S2 and the S16 with *Wolbachia *species and finally the ribosomal protein L13 with *Pseudomonas aeruginosa*. No synteny conservation was observed (see additional file 6: Recombination events).

### Divergence time and the creation of mitochondria

**Figure 4:**
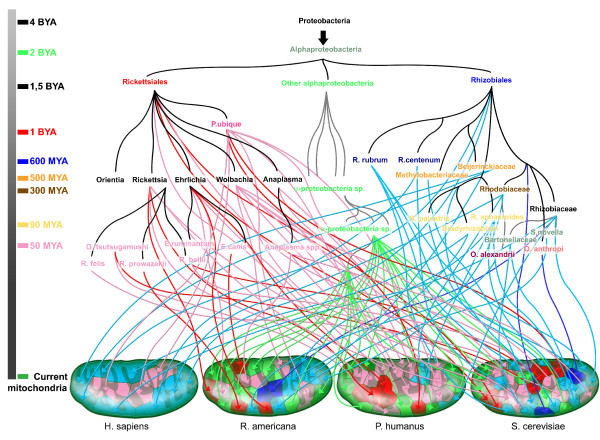
**The rhizome of mitochondria**. The origin of each gene is represented along with the time scale of the species divergence. Dark blue and red arrows are for sister taxa with high bootstrap values, and light blue and pink arrows for sister taxa with low bootstrap values. Green arrows are for sister taxa from the *Alphaproteobacteria *subgroup. Colors on the time scale coincide with the emergence of the corresponding colored species.

We estimated the approximate divergence time of the recombined species and the times when genes were most likely introduced into the genomes of the four studied types of mitochondria using 16S rRNA and gene sequence phylogenies and an estimated evolutionary rate of 1-2% per 50 million years. *Rickettsiales *and *Rhizobiales *most likely diverged 1.5 billion years ago (BYA). Their fusion probably created the first mitochondrion approximately 1 BYA. The *R. americana *mitochondrion also contains genes that were likely acquired 500-600 million years ago (MYA) from proto-*Rickettsiae*, proto-*Ehrlichia*, proto-*Wolbachia*, proto-*Rhodobiaceae *and proto-*Bradyrhizobiaceae *species. More genes were added later, between 30-90 MYA, from current rickettsial species and current *Rhizobiales*. All elements from other *Alphaproteobacteria *were added recently, approximately 40-70 MYA (Figure 4). The *Homo sapiens *mitochondrion contains mostly *Rhizobiales *genes obtained between 500-600 MYA, while three genes of *Rickettsiales *origin were in place much earlier, approximately 1 BYA. The *P. humanus *mitochondrion contains mostly *Rickettsiales *genes, some of which were in place in the very beginning of the creation of mitochondrion, while others were added later around 300-500 MYA. It also contains some *Rhizobiales *and other alpha-proteobacterial genes, which were added more recently, around 40-90 MYA. Finally, the *S. cerevisiae *mitochondrion contains primarily *Rhizobiales *genes that were gained 500-600 MYA, but it also contains some alpha-proteobacterial elements that were added later, approximately 40-90 MYA. As for the genes of *Rickettsiales *origin, most of them were added recently from current rickettsial, *Anaplasma*, oriential and *Ehrlichia *species, whereas some of these genes are older and were derived from ancestral *Rickettsiales *species (Figure 4).

## Discussion

It is thought that mitochondria originated through an endosymbiotic event that occurred between the proto-*Rickettsiales *and a proto-eukaryotic cell during the early stages of eukaryotic evolution [13-15]. Molecular phylogenomic analyses of mitochondrial proteins place mitochondria in the alphaproteobacterial subdivision, whereas several reconstructions place mitochondria specifically in the *Rickettsiaceae *family [16-20] or even at the root of the *Rickettsiales *order [8]. It has also been proposed that *Rickettsiae *and mitochondria shared a last common ancestor that was probably a parasite of proto-eukaryotic cells [21]. Even though most studies have argued that mitochondria are closely related to the *Rickettsiales *order, recent studies of the mitochondrion of the green algae *Chlamydomonas reinhardtii *have proposed that most of the species' mitochondrial protein sister taxa are members of the *Rhizobiales *and the *Rhodobacterales *rather than the *Rickettsiales *order [6]. It has also been proposed that the mitochondrial ancestor is a mix of different eubacterial genes, some of which are still conserved in alphaproteobacterial genomes [22]. Furthermore, a recent study using the *Saccharomyces cerevisiae *mitochondrion demonstrated that a certain chimerism of bacterial genomes occurred during the formation of mitochondria [9].

**Figure 5:**
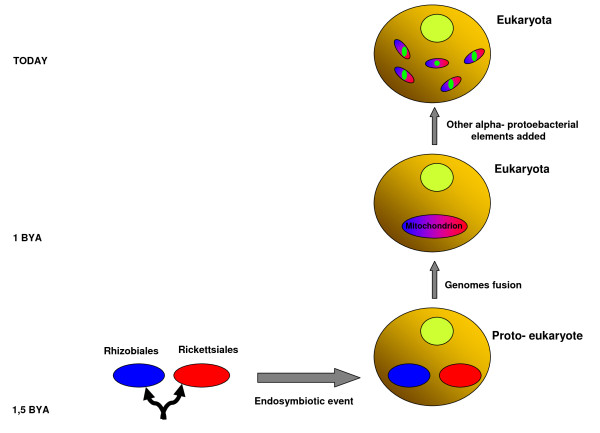
**The creation of mitochondria**. *Rhizobiales *and *Rickettsiales *ancestors diverged approximately 1.5 billion years ago. They both found themselves in a proto-eukaryote after an endosymbiotic event one billion years ago. Genome fusion took place, creating mitochondria and eukaryotes. Mitochondria evolved further by acquiring new elements from other *Alphaproteobacteria*. *Rhizobiales *are represented in blue, *Rickettsiales *in red and other *Alphaproteobacteria *in green.

We wanted to go a step further and investigate the phenomenon of mosaicism in mitochondria, revisit the idea of a common ancestor and try to understand the origin of the mitochondrion, with the ultimate goal of building a genealogical tree for mitochondria. Our results demonstrate a true mosaic structure that is different for each of the four studied types of mitochondria. Indeed, the origin of the mitochondrial genes does not seem to be limited to the *Rickettsiales*. Quite often, as in the study of Atteia *et al*. [6], mitochondrial protein sister taxa are members of the *Rhizobiales*. Moreover, in some cases, and especially in the case of *Reclinomonas americana *mitochondria, other *Alphaproteobacteria *were found to be protein sister taxa. Our data suggest that the genomes of a *Rickettsiales *and a *Rhizobiales *ancestor likely merged during the first endosymbiotic event in a proto-eukaryotic cell approximately one billion years ago. This fusion coincides with the rise of eukaryotes and mitochondria. Mitochondria were created as a mosaic and later incorporated more elements through lateral gene transfer (LGT) or recombination events from other Alphaproteobacteria (Figure 5). The use of four types of mitochondria from four different organisms (protozoa, yeast, louse, humans) allowed us to demonstrate that the mitochondria of different organisms are composed of different elements and have different genealogical trees. The sister taxa of *Saccharomyces cerevisiae *and human mitochondrial proteins are mostly members of *Rhizobiales*, whereas the sister taxa of *R. americana *mitochondrial proteins are mostly members of *Rickettsiales*. There are additional alphaproteobacterial elements in the *S. cerevisiae *mitochondrion than in the three other types of mitochondria. In addition, we detected some recent recombination events in *R. americana *and in *P. humanus *mitochondria, mostly involving *Rickettsiales*. Indeed, using the louse mitochondrion allowed us to demonstrate how the mitochondria creation model is not fixed and that the mitochondria were not created through a single event. The mitochondrial genome of *P. humanus *is fragmented into 18 mini-chromosomes. This event likely took place after the emergence of the mitochondrion through a series of events involving the excision and rejoining of fragments over a long period of time [23].

The high degree of genetic transfer into eukaryotic genomes from bacteria may seem surprising. However, the *Wolbachia *paradigm confirms this mode of recombinogenic genome creation. Comparative genomic studies have provided evidence for progressive LGTs from *Wolbachia *to arthropods, insects and nematodes, and transfers involve nearly the entire *Wolbachia *genome [24-26]. Other cases are observed in human genomes, as *Trypanosoma cruzi *sequences have integrated into human genomes [27], and HHV6 sequences were also found integrated in patient genomes and were even transmitted to descendants [28]. Finally, a recent study supported a scenario in which *Myxococcales *may have contributed key metabolic genes to the first eukaryotes [22]. Moreover, a recent analysis indicates both homologous and non-homologous recombination between the minichromosomes in the mitochondria of the human body louse [29]. It has therefore already been demonstrated that LGT and recombination events are possible, and it may be easy to transfer sequences from microorganisms infecting eukaryotic cells continuously over such a long period of time.

The creation of mitochondria may not have occurred as a single event but as multiple successive events. Some of these events are very old and constitute the basis of each species' mitochondria. These events were followed by events that are more recent and by the addition of elements originating from current species. The times at which elements were added and the parental species of each gene in mitochondrial genomes are not the same for different species. These data constitute strong evidence that mitochondria do not have a single common ancestor, but probably have numerous ancestors comprising proto-*Rickettsiales*, proto-*Rhizobiales*, and proto-*Alphaproteobacteria*, as well as current alphaproteobacterial species. Mitochondrial genomes have also undergone genome reduction. The gene loss of the mitochondria of different organisms is more or less abundant and has resulted from the transfer of genes to the host nucleus and from the irreversible loss of redundant genes [30,31]. The observation that mitochondrial genomes vary enormously in size and gene content suggests that gene transfer might be dependent on environmental conditions. Indeed, a large portion of mitochondrial genes have been transferred to the nucleus; however, it is difficult to identify mitochondria-targeted genes encoded in the nuclear genomes. Bioinformatic analyses have been uncharacteristically unsuccessful in estimating the number of nuclear genes that code for mitochondrial proteins [32]. It is thought that there are approximately 1200 such genes in the human genome. This difficulty led to our decision to not include other mitochondria-targeted genes encoded in the nucleus. Nevertheless, the *R. americana *mitochondrion contains the least derived mitochondrial genome with the largest coding capacity and is therefore representative of the ancestral mitochondrion [33].

## Conclusions

The most plausible scenario of the origin of the mitochondrion is that ancestors of Rickettsiales and *Rhizobiales *merged in a proto-eukaryotic cell approximately one billion years ago and that this fusion was followed by probable gene loss, genomic rearrangements and the addition of alphaproteobacterial elements through ancient (500-600 MYA) and more recent (30-90 MYA) recombination events and LGTs. Mitochondrial evolution constitutes a rhizome (Figure 4). The tree of life (TOL) is not sufficient to explain the chimeric structure of current genomes. The TOL and Darwin's theory on the common descent of species are contradicted by more and more evidence from genomic analyses, suggesting that there are no two genomes with a similar history [34,35]. This theory is striking in the case of mitochondria and, in particular, when looking at the case of the multichromosome mitochondrion *of P. humanus*. This multichromosomal situation clearly demonstrates that mitochondria do not have a stable or unique form; therefore, the history of their evolution cannot be the same. This likelihood is also supported by the fact that they do not have the same number of genes. Gene loss and transfer events to the nucleus were not the same for the mitochondria of different organisms. For the first time, we present evidence that the common ancestor theory is likely incorrect, even in the case of organelles such as mitochondria. Indeed, the sources of mitochondrial genes were not the same between different organisms. Some elements were established quite early during the evolution of mitochondria (approximately one billion years ago), while others were added much later, after the divergence of the different rickettsial species (Figure 5). Moreover, recombination and gene exchange events occur so often in all organisms and to such an extent that it seems absolutely plausible that an elevated rate of such events took place in mitochondria as well, especially when we consider their long existence of approximately 1 BY, which gave them the opportunity to recombine, rearrange and shape their genomes in various ways. In the case of organelles, genealogical trees better represent these multiple origins of the genomic repertoire of mitochondria. Unfortunately, the reconstruction of the rhizome has its limits. We are able to detect signals from two of three ancestral generations, and based on these signals, we have suggested scenarios that retrace history back a couple of billion years, but after a certain point, the signals are no longer detectable, and the accurate determination of the gene repertoire of ancestors is not possible. However, there is sufficient evidence to track evolution quite far and to pose the following idea: if even organelles such as mitochondria are mosaics, then the theory of a single common ancestor and a top-down tree do not reflect our current state of knowledge.

## Methods

### Sequence similarity search

We analyzed the sequences of the 67 protein-coding genes of the *Reclinomonas americana *mitochondrion, the 13 protein-coding genes of the human mitochondrion, the 110 mitochondria-related proteins of *Pediculus humanus *(37 internal mitochondrial genes found on 18 mini-chromosomes and 73 nuclear genes) and a total of 91 mitochondria-related proteins (both internal to mitochondria and nuclear genes) of *Saccharomyces cerevisiae *that gave matches to 5 alphaproteobacterial species (*Rickettsia prowazekii*, *Rhodospirillum rubrum*, *Rhodopseudomonas palustris*, *Rhodobacter sphaeroides *and *Ochrobactrum anthropi*) in the study of Abhishek *et al*. (2011) [9] using a BLASTP search. Each gene sequence of the identified sister taxa of the mitochondria was blasted against the redundant NR database to identify any previous LGTs in Alphaproteobacteria. All results were filtered using an e-value cut-off of 10-15.

### Phylogenetic analyses

All of the best matches for each of the mitochondrial (and sister taxa) proteins in each of the four organisms were used to construct Maximum Likelihood (ML) phylogenetic trees. Alignments were performed with ClustalX2 [36], and trees were constructed and visualized using Mega4 [37]. Bootstrap values were computed for all trees using 100 replications. Trees were then classified according to the bootstrap value obtained at the mitochondrion/sister taxon node. The percentage of LGTs was calculated for each of the four mitochondria, and the information *on R. felis *was retrieved from the study of Merhej *et al*., 2011 [35]. A χ2 statistical test was performed to determine possible significant differences.

### Evidence for recombination events

To identify possible recombination events, we first searched for repeat elements in the three mitochondrial genomes using the REPuter tool [38]. We also used the RAT [39] on each protein sequence to detect potential recombination events.

### Divergence time calculation

The divergence time of recombined species was calculated using 16S rRNA phylogenies and the molecular clock option of the Mega4 program, assuming a relatively constant rate of evolution of 1 to 2% per 50 million years [40,41].

## List of abbreviations

RAT: Recombination analysis tool; BYA: billion years ago; MYA: million years ago; LGT: lateral gene transfers; TOL: tree of life; ML: maximum likelihood.

## Competing interests

The authors declare that they have no competing interests.

## Authors' contributions

DR designed the research project. KG performed the genomic analysis and analyzed the data. KG and DR wrote the paper. RD revised the paper. All authors read and approved the final version.

## Reviewers' comments

### Reviewer's report 1

William Martin, Institut of Botanic III, Heinrich-Heine-University, Düsseldorf

#### Reviewer 1

This paper examines individual gene phylogenies in an attempt to examine the origin of mitochondria. It comes in the wake of a much more thorough study by Stephen Giovannoni's group (Thrash et al., Phylogenomic evidence for a common ancestor of mitochondria and the SAR11 clade, Scientific Reports Volume: 1, Article number: 13 DOI: doi:10.1038/srep00013) but taxon sampling is not the main problem with this paper. The problem is the interpretation of the the results. The authors find, like Abishek et al., 2011 (ref 9) and many of us over the last 10 years or so have found, that different mitochondrial genes do not trace to the same source in phylogenetic trees. Georgiades and Raoult interpret that as evidence to indicate that mitochondria acquired different genes from different sources, and that is probably how many folks would have interpreted such results 15 or so years ago. But what Georgiades and Raoult forget to mention is that their interpretation is very one-sided (and to some extent upside-down-and-backwards): they assume 1) that all of the lateral transfer occurred from different bacteria to mitochondria (and to the nucleus in the case of their reanalysis of Abishek's data) and 2) that the free-living prokaryotes from which these genes are presumed to have been donated never undergo lateral transfer of genes. That is excruciatingly unlikely, considering what we know about LGT among prokaryotes. Free-living prokaryotes undergo LGT all the time. [If the authors need a reference for that, there are the 700 papers that cited WF Doolittle Science 1999 or the 1100 papers that cited Ochman et al., Nature 2000.] And they did at the time that mitochondria arose too (1.5 Ga at least; Javaux et al., Nature 2001). The result of LGT among free-living relatives of mitochondria (and chloroplasts) prior to and subsequent to the origins of those organelles is that genes brought in to the eukaryotic lineage by the mitochondrion might now look in a phylogenetic tree as if they had been brought in by a different lineage. I have been making that point since 1999 (1-8).

1. Martin W: Mosaic bacterial chromosomes--a challenge en route to a tree of genomes. BioEssays 21:99-104 (1999).

2. Rujan T, Martin W: How many genes in Arabidopsis come from cyanobacteria? An estimate from 386 protein phylogenies. Trends Genet. 17:113-120 (2001).

3. Schnarrenberger C, Martin W: Evolution of the enzymes of the citric acid cycle and the glyoxylate cycle of higher plants: A case study of endosymbiotic gene transfer. Eur. J. Biochem. 269:868-883 (2002).

4. Martin W, Rujan T, Richly E, Hansen A, Cornelsen S, Lins T, Leister D, Stoebe B, Hasegawa M, Penny D: Evolutionary analysis of Arabidopsis, cyanobacterial, and chloroplast genomes reveals plastid phylogeny and thousands of cyanobacterial genes in the nucleus. Proc. Natl. Acad. Sci. USA 99:12246-12251 (2002).

5. Esser C, Ahmadinejad N, Wiegand C, Rotte C, Sebastaini F, Gelius-Dietrich G, Henze K, Kretschmann E, Richly E, Leister D, Bryant D, Steel MA, Lockhart PJ, Penny D, Martin W: A genome phylogeny for mitochondria among a-proteobacteria and a predominantly eubacterial ancestry of yeast nuclear genes. Mol. Biol. Evol. 21:1643-1660 (2004).

6. Esser C, Martin W, Dagan T: The origin of mitochondria in light of a fluid prokaryotic chromosome model. Biol. Lett. 3:180-184 (2007).

7. Deusch O, Landan G, Roettger M, Gruenheit N, Kowallik KV, Allen JF, Martin W, Dagan T: Genes of cyanobacterial origin in plant nuclear genomes point to a heterocyst-forming plastid ancestor. Mol. Biol. Evol. 25:748-761 (2008).

8. Atteia A, Adrait A, Brugière S, van Lis R, Tardif M, Deusch O, Dagan T, Kuhn L, Gontero B, Martin W, Garin G, Joyard J, Rolland N: A proteomic survey of Chlamydomonas reinhardtii mitochondria sheds new light on the metabolic plasticity of the organelle and on the nature of the alpha-proteobacterial mitochondrial ancestor. Mol. Biol. Evol. 29:1533-1548 (2009).

And it seems now that folks are beginning to get it, for example as in: Richards TA, Archibald JM (2011) Gene transfer agents and the origin of mitochondria. Curr Biol 21: R112-R114. But the point is not obvious, so I will explain a bit more. And none of this is to say that Georgiades and Raoult should cite my papers, it is just to substantiate the point: The ancestor of mitochondria lived at least 1.5 billion years ago, and it possessed in its chromosome(s) a specific collection of genes -- by becoming an endosymbiont and an organelle it became cut off from standard gene flow with free-living bacteria (just like *Buchnera*, *Wolbachia *or *Rickettsia *become cut off). Hence the origin of mitochondria was a sampling process of one genome's worth of ancient eubacterial gene diversity (making no statement about the size of that genome). The closest relatives of the mitochondrial ancestor living 1.5 billion years ago (1/3 of earth's age ago) had about the same collection of genes, but over time they donated some to other lineages and collected some from yet other lineages, etc. etc. etc. and on the bottom line we do not know exactly how much gene transfer among free-living prokaryotes went on, but we know it was a lot! After all, modern alphaproteobacterial genomes are highly chimaeric themselves, and it is silly to expect that any modern bacterium should possess exactly the same collection of genes as the ancestor of mitochondria possessed. For example, a collection of 82 alphaproteobacterial genomes contains 27,810 gene families (excluding singletons) (9), the proteobacteria harbour 74667 families (9). Those genes were not all present in the "last common ancestor" of alphaproteobacteria (or proteobacteria, respectively), otherwise its genome would have been too big to be true (the Genome of Eden problem). We have to accept that genes really are on the move across prokaryote genomes over time and we know the mechanisms (transformation, transduction, conjugation, gene transfer agents). That does not make the orign of mitochondria easier to reconstruct, but it does mean that if we are to allow lateral gene transfer into the issue at the origin of mitochondria, as Georgiades and Raoult laudably are doing, we have to consider known mechanisms (LGT among prokaryotes) first (please), before we start making radical claims about lateral transfers to mitochondrial genomes.

9. Kloesges T, Martin W, Dagan T: Networks of gene sharing among 329 proteobacterial genomes reveal differences in lateral gene transfer frequency at different phylogenetic depths. Mol. Biol. Evol. 28: 1057-1074 (2011).

Overall, the situation is not that dire. Yes, mitochondria are chimaeric, because the ancestor of mitochondria was chimaeric. But the simple observation that all mitochondrial genes are still a subset of the *Reclinomonas *gene set indicates in the most straightforward manner that mitochondria have gone genome reduction in evolution, not gene acquisition. So the figure that Georgiades and Raoult present here is wrong (though pretty), but the observations upon which it is based remain valid. It is just that gene transfers among prokaryotes (my model) explains both Georgiades and Raoult findings as well as chmiaerism among prokaryotes, where as their model only accounts for mitochondrial chimaerism while assuming (and actually demanding) that free-living prokaryotes have been immune to LGT. If they want a picture of this issue that is constructed via calculations by a computer rather than drawn by hand as an artist's impression (like I did in ref. 1, 1999), see (10).

10. Dagan T, Martin W: Getting a better picture of microbial evolution en route to a network of genomes. Phil. Trans Roy. Soc. Lond. B 364: 2187-2196 (2009). With thanks for your patience and trusting that you will understand my points here and rewrite the paper accordingly and reinterpret the observations to obtain a less radical inference about mitochondrial history, I remain with my best regards,

Yours sincerely

Bill Martin

### Authors' response

*Thank you for the time and energy you spent giving us such beautiful and interesting remarks, which we took under consideration. It is true that, in the first version, we did not think about considering previous LGTs in Alphaproteobacteria. Therefore, in the revised version, we looked for LGTs by re-BLASTing all the sequences of the mitochondria sister taxa against NR and building phylogenies including the hits of these BLASTS. However, no previous LGTs were detected except for two cases, for which ancestors acquired genes before the mitochondria did. These cases include the ribosomal protein L6, which seems to have been transferred to the alphaproteobacterium Methylibium petroleiphilum from Betaproteobacteria, and the aconitate hydratase protein that was passed over to some members of the Rickettsiales from Gammaproteobacteria **(Lines: 125-139; 320-323; 325)**. None of the other phylogenies revealed a previous acquisition of genes by ancestors. The idea of mosaicism in ancestral genomes is interesting, but even if rickettsial genomes are chimerical, they are not as chimerical as mitochondria. The proportion of mosaicism in R. Americana (34%), H. sapiens (84.6%) and S. cerevisiae (31.8%) mitochondria is statistically more elevated than the proportion of mosaicism in Rickettsia species (12%) ***(See **Figure 1;*Lines: 125-139; 329-332). Moreover, in a recent study identifying LGT events in rickettsial species, we identified very small numbers of such events (6) in current rickettsial species *[42]. *LGTs and recombination events are not rare, and it is easy for organisms to exchange sequences continuously over different periods of time. Mitochondria appeared approximately 1 BYA; it is therefore plausible that such events occurred very often in mitochondrial genomes **(Lines: 247-255)***.

*Mitochondrial genome reduction was also considered and included in the revised version. The gene loss of mitochondria of different organisms is more or less abundant and resulted from the transfer of genes to the host nucleus and the irreversible loss of redundant genes *[24,25]***(Lines: 263-276)**. There are two possible hypotheses on the creation of mitochondria: either sequences were rearranged and exchanged after the emergence of mitochondria by fusion of the ancestors of Rickettsiales and Rhizobiales that merged in a proto-eukaryotic cell or current mitochondrial genes were gained by mitochondrial ancestors and were selected before the emergence of mitochondria. Our evidence demonstrates the first scenario **(Lines: 279-283; 287-292; 294-301)***.

*Finally, by adding the Pediculus humanus mitochondrion in the revised version **(Lines: 32-33; 43-44; 79; 80; 82; 115-121; 154-155; 174-177; 190-194; 314-316)**, we clearly demonstrate that there is not a unique ancestral model that is fixed and rigid. The mitochondrial genome of P. humanus is fragmented into 18 mini-chromosomes*. *In some cases, multichromosomes may allow for enhanced gene exchange. This event likely took place after the emergence of the mitochondrion through a series of events involving the excision and rejoining of fragments over a long period of time **(Lines: 50; 237-242)***.

### Reviewer's report 2

*Arcady Mushegian, Department of Bioinformatics, Stowers Institute for Medical Research, Kansas City, Missouri, USA*.

#### Reviewer 2

The authors ask: which representative of Alphaproteobacteria may have given rise to mitochondria? Their answer is that there were multiple round of acquisition of mitochondrial genes from different Alphaproteobacteria ("rhizosphere" of ancient eukaryotes), with recombinational gene replacement and lineage-specific loss of mitochondrial genes, so that the mitochondrial genomes (plus nucleus-encoded mitochondrial proteins) of present-day eukaryotic lineages are the patchwork of old and new genes, mixed in different proportions and going back to different (but still mostly alphaproteobacterial) ancestors. These ideas are interesting, but I do not think that the data presented by the authors actually support their case that well. Certainly, in the Discussion, the authors are carried away with the statements such as "The TOL and Darwin's theory on the common descent of species is contradicted by more and more evidence from genomic analyses, suggesting that there are not two genes with a similar history" (I see plenty of evidence in the genomic data that many pairs of genes have very similar history, and surely there may be some pairs with the identical history)

### Authors' response

*This statement was rephrased as the following*: *"The TOL and Darwin's theory on the common descent of species are contradicted by more and more evidence from genomic analyses, suggesting that there are no two genomes with a similar history" **(Line: 286)***.

#### Reviewer 2

and "For the first time, we present evidence that the common ancestor theory is incorrect, even in the case of organelles such as mitochondria" (I see no earth-shattering evidence to that effect in this study, as I will try to point out below).

### Authors' response

*This was rephrased as the following*: *"For the first time, we present evidence that the common ancestor theory is likely incorrect, even in the case of organelles such as mitochondria"**(Line: 293)***.

#### Reviewer 2

The main device used by the authors is phylogenetic trees of protein families which have as their members select mitochondria-encoded and nuclear-encoded mitochondrial proteins from three eukaryotic species. The selection of these proteins is inconsistent between a protist, yeast and human: why only mitochondria-encoded proteins are used in the case of human and protest mitochondria, whereas only in yeast this is supplemented with mitochondria-targeted genes encoded in the nuclear genome?

### Authors' response

*Unfortunately, it is not easy to identify mitochondria-targeted genes encoded in nuclear genomes. Indeed, bioinformatic analyses have been uncharacteristically unsuccessful in estimating the number of nuclear genes that code for mitochondrial proteins (estimates range from 349-2,897 in different species) *[27]. *It is thought that are approximately 1200 such genes in the human genome. We only completed our Saccharomyces cerevisiae mitochondrial database with such genes because our study was initially inspired by the study by Abhishek et al., 2011 *[9]*, in which S. cerevisiae mitochondria-targeted genes encoded in the nuclear genomes were used. We were also able to use the mitochondria-targeted genes of Pediculus humanus that are annotated in the NCBI database. On the other hand, the Reclinomonas americana mitochondrion contains the least-derived mitochondrial genome with the largest coding capacity and encodes 97 genes *[32]***(Lines: 263-276)***.

#### Reviewer 2

The other inconsistency is manifest when we compare the trees for various protein families. Most of these trees contain proteins from different subsets of Alphaproteobacteria: many trees have no representatives from *Rickettsiales*, even when it is known that these species have the proper orthologs (e.g., many ribosomal proteins), some have no representatives from *Rhizobiales*, etc. I suspect that this is an artefact of "filtering the BLASTP results" by E-value of 10^-15, for which there cannot be good scientific justification. I trust that the authors would agree with me that the statement "the nearest tree neighbor of an eukaryotic mitochondrial protein comes from a taxon other than Rickettsiales" makes little sense when a bona fide ortholog of the eukaryotic protein exists in Rickettsiales but has not been included into the alignment from which the tree was inferred.

### Authors' response

*We consider an e-value of 10^-15 ^to be stringent enough to obtain reliable alignments and homologs. In Abhishek's study *[9]*, an e-value cut-off of 10^-22 ^was used*.

#### Reviewer 2

Even with these quite substantial technical shortcomings, a look at the Tables 1, 2, 3 displays a quite strong trend that seems to argue against the authors' interpretation. Namely, when the partition with a mitochondrial protein in it has strong statistical support, the neighbor is usually from *Rickettsiales *or, less commonly, from *Rhizobiales*; and when the neighbor is from another clade of Alphaproteobacteria, the partition tends to be supported weakly. This, in my opinion, is good evidence that the overwhelming majority of mitochondrial genes comes from the *Rickettsiales *clade. This signal will probably be stronger when more consistent collection of homologs will be analyzed.

### Authors' response

*This explanation could be a possibility; however, we know that when bootstraps are not significant, adding some species into the phylogeny will result in a more elevated bootstrap. The weak bootstrap values could also be due to many recombination events that were not showcased in our study*.

#### Reviewer 2

Another comment has to do with the evolutionary timing. The authors cite the time of split between *Rickettsiales *and *Rhizobiales *of 1.5 BYA. Incidentally, this same time point is considered by many authors to be the lower bound of the eukaryote age, based on the fossil record; indeed, eukaryotes may be even older. But if both dates are correct, then there were no separate clades of *Rickettsiales *and *Rhizobiales *at the time of the eukaryote origin, only the common alphaproteobacterial ancestor. In this case, the "patchiness" of mitochondria is even less, as distinction between the present-day rickettsial and rhizobial neighbors is moot (and the difference should be screened for parallel evolution, branch attraction artefacts, etc). This is not to say that there was no introgression and replacement of mitochondrial genes (whether mitochondria-encoded or nuclear) by later rounds of horizontal transfer from bacteria - by the way, not only Alphaproteobacteria, and indeed the search for mitochondrial genes transferred from more distant lineages could be a good way to develop this theme. But currently, the evidence is probably just not there.

### Authors' response

*Eukaryotes emerged at about the same time as mitochondria after the first endosymbiotic event which took place between 1.5 and 1 BYA, not earlier. During that time, Rickettsiales and Rhizobiales diverged. As for other LGTs, that point was taken under consideration in the revised version (see Reviewer 1)*.

### Reviewer's report 3

*Eugene V. Koonin, NCBI, NLM, NIH, Bethesda, MD 20894, USA*.

#### Reviewer 3

In this provocative article, Georgiades and Raoult propose a 'rhizome' scenario of mitochondrial genome evolution according to which "The most plausible scenario of the origin of the mitochondrion is that ancestors of *Rickettsiales *and *Rhizobiales *merged in a proto-eukaryotic cell approximately one billion years ago". Beyond any question, this is a provocative and novel suggestion. Moreover, I should note that the rhizome (perhaps, more precisely, network or web) of prokaryote evolution is generally valid beyond doubt. It is no exaggeration to maintain that any prokaryote genome is a palimpsest of multiple gene exchange, replicon fusion and recombination events. More specifically, however, I think the authors of this paper are missing two key points.

First, exactly because each prokaryote genome is a complex chimera, it is impossible to accurately reconstruct the gene repertoire of the alpha-proteobacterial ancestor of the mitochondrion. Genomes of modern alpha-proteobacteria are extremely poor guides for such a reconstruction. This point is very clearly demonstrated and emphasized in the important paper by Esser, Martin and Dagan (Esser C, Martin W, Dagan T. The origin of mitochondria in light of a fluid prokaryotic chromosome model. Biol Lett. 2007 Apr 22;3(2):180-4). Thus, the chimeric character of the mitochondrial genome is quite likely to be accounted for by the mosaicism of the ancestral alpha-proteobacterial genome.

### Authors' response

*We are fully aware of the limits of such a reconstruction, and we do criticize these limits in our Discussion*. *We are able to detect signals from two of three ancestral generations, and based on these signals, we suggested scenarios that retrace history for a couple of billion years, but after a certain point, the signals are no longer detectable, and an accurate determination of the gene repertoire of ancestors is not possible **(Lines: 302-306)**. The case of previous LGTs in Alphaproteobacteria was taken under consideration in the revised version (See Reviewer 1). Furthermore, as discussed in our response to Reviewer 1, the mosaicism of rickettsial species is significantly lower than the mosaicism of mitochondria (See *Figure 1, *Reviewer 1). Moreover, when we look in the pan-genome of Rickettsia, it is obvious that even though there are some LGT events in their genomes, they are really not very numerous; the biggest portion of the Rickettsia pan-genome is composed of specific genes (*Figure 2*) **(Lines: 125-139)***.

#### Reviewer 3

In addition, it is well known that after the endosymbiosis numerous genes from the endosymbiont have been relocated to the host nuclear genome. Although this set of genes is difficult to delineate precisely, there is reasonable confidence regarding the transfer of several hundred genes, so these genes necessarily have to be taken into account in any reconstruction of mitochondrial genome evolution. Thus, in my view, the rhizome scenario of mitochondrial evolution, however interesting, runs afoul of the Occam razor.

### Authors' response

*Indeed, delineating the set of mitochondria-targeted nuclear genes is very difficult, which is why we were not able to identify them in R. americana or H. sapiens mitochondria (see Reviewer 2)*.

*We are convinced that parsimony is essential, but the simplest way of explaining mosaicism is not to say that an endosymbiont acquired a great proportion of genes from its ancestor but rather that mitochondria continued to evolve by integrating exogenous sequences. The fragmented chromosome of the P. humanus mitochondrion, used in our revised version, is the best evidence showing the progressive evolution of mitochondria over a long period of time, even though such a series of events is not parsimonious *[23]***(Lines 237-242)***.

*Recent evidence has shown that exogenous sequences from Trypanosoma cruzi and the HHV6 virus were also integrated into the human genome, thus demonstrating a continuous chimeric formation. Furthermore, if such events are visible in such a short timescale as in the human genome, we do not see why it would not be visible in something as old as mitochondria **(Lines: 247-253; 294-301)**. If we consider a post-Darwinian model of evolution, mosaicism is the simplest explanation and therefore most likely the correct one according to Occam's razor*.

## Supplementary Material

Additional file 1 ***Reclinomonas americana *mitochondrial phylogenies**. Click here for file

Additional file 2 ***Homo sapiens *mitochondrial phylogenies**. Click here for file

Additional file 3 ***Saccharomyces cerevisiae *mitochondrial phylogenies**. Click here for file

Additional file 4 ***Pediculus humanus *mitochondrial phylogenies**. Click here for file

Additional file 5 **Previous horizontal gene transfers in Alphaproteobacteria**. Click here for file

Additional file 6 **Recombination events in the Reclinomonas americana and *Pediculus humanus mitochondria***. Click here for file
