# Supplementary material for: The rhizome of Reclinomonas americana, Homo sapiens, Pediculus humanus and Saccharomyces cerevisiae mitochondria
Source: Biol Direct. 2011 Oct 20;6:55. doi: 10.1186/1745-6150-6-55 (PMC3214132; doi:10.1186/1745-6150-6-55)

# Ribosomal protein L2

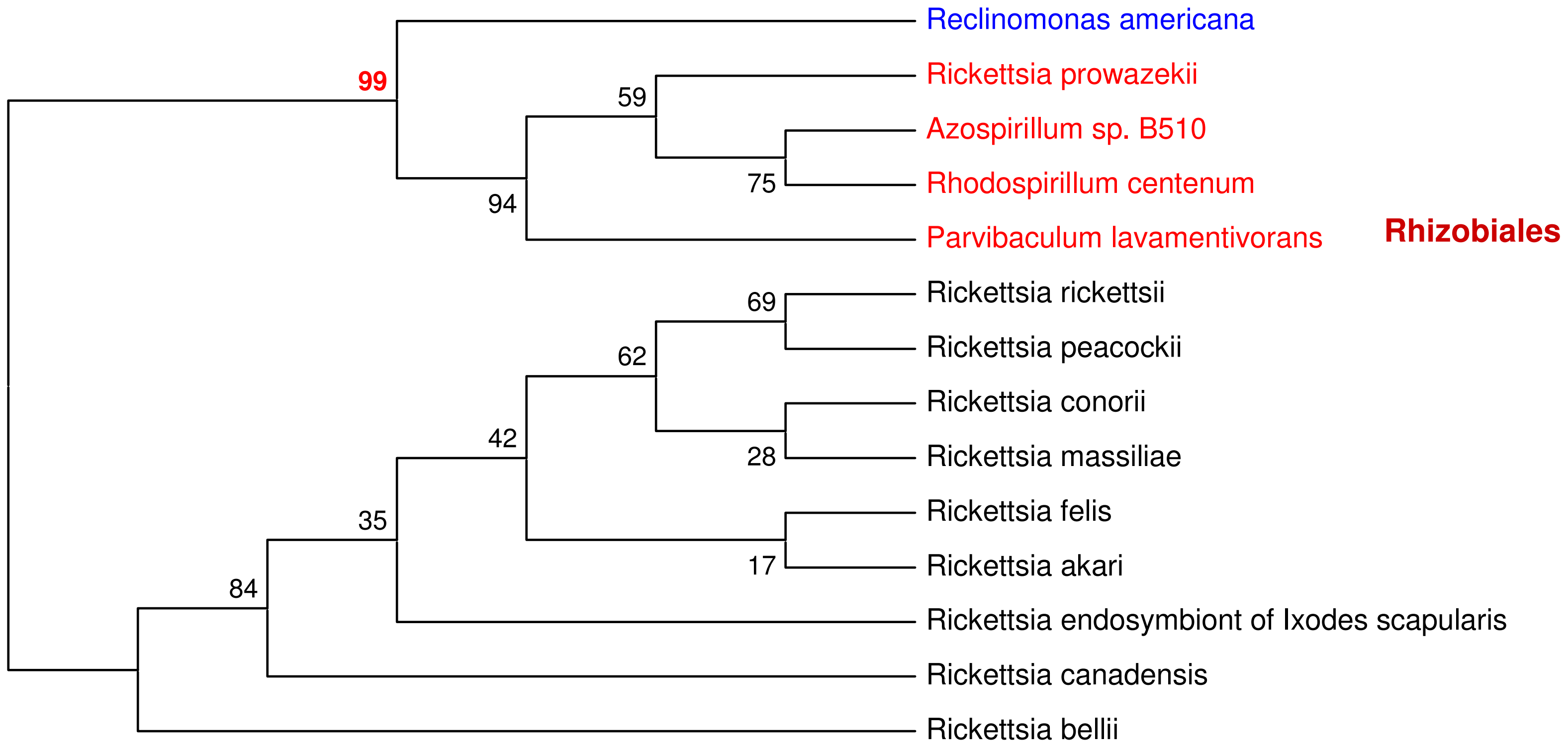

# RNA polymerase subunit beta

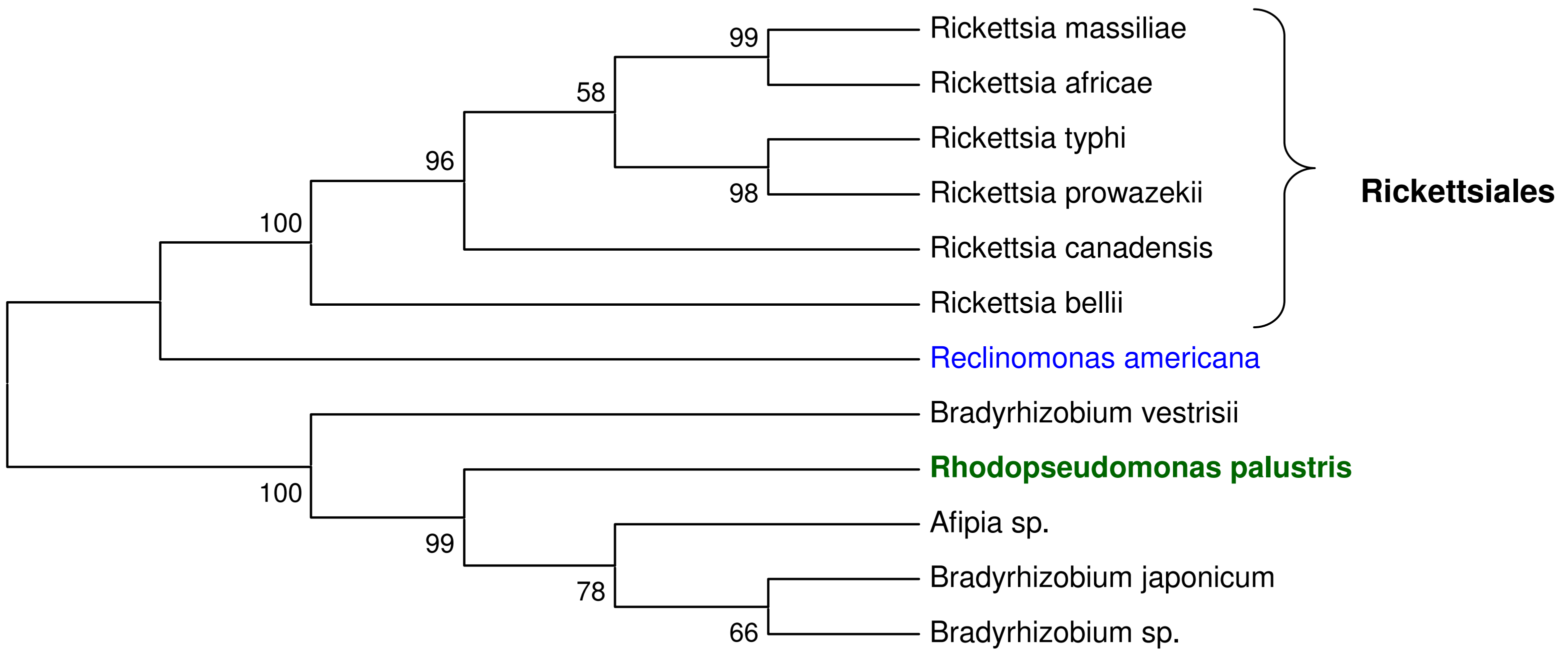


# RNA polymerase subunit beta’

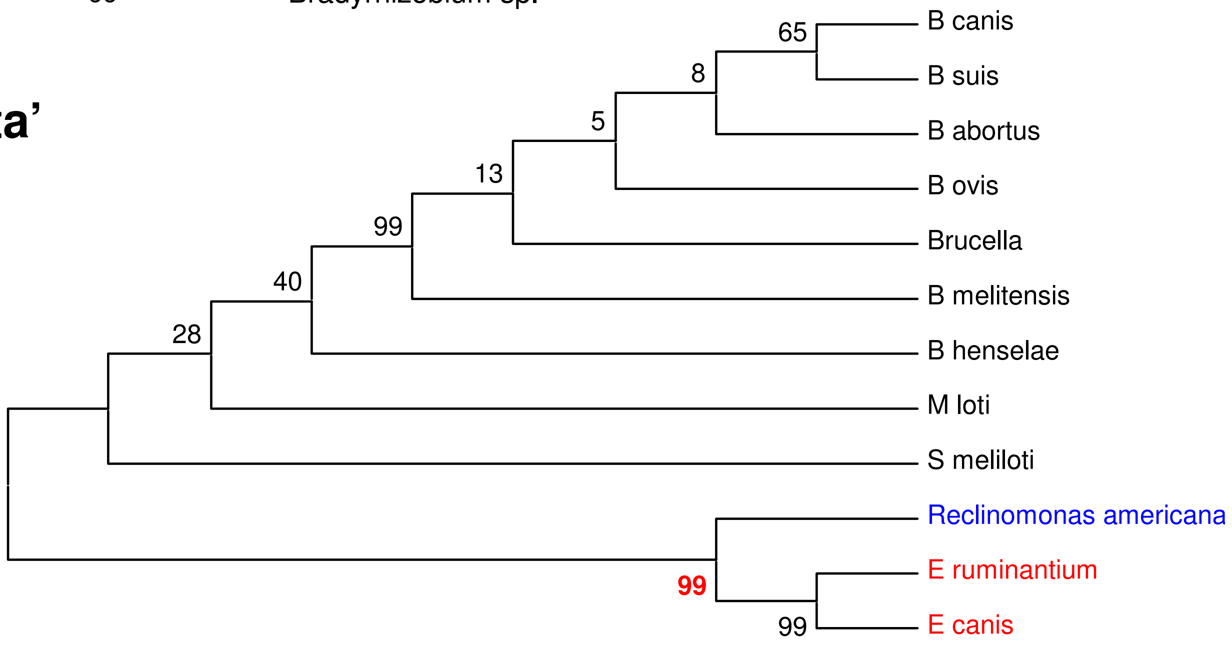

## Succinate ubiquinone oxidoreductase 2

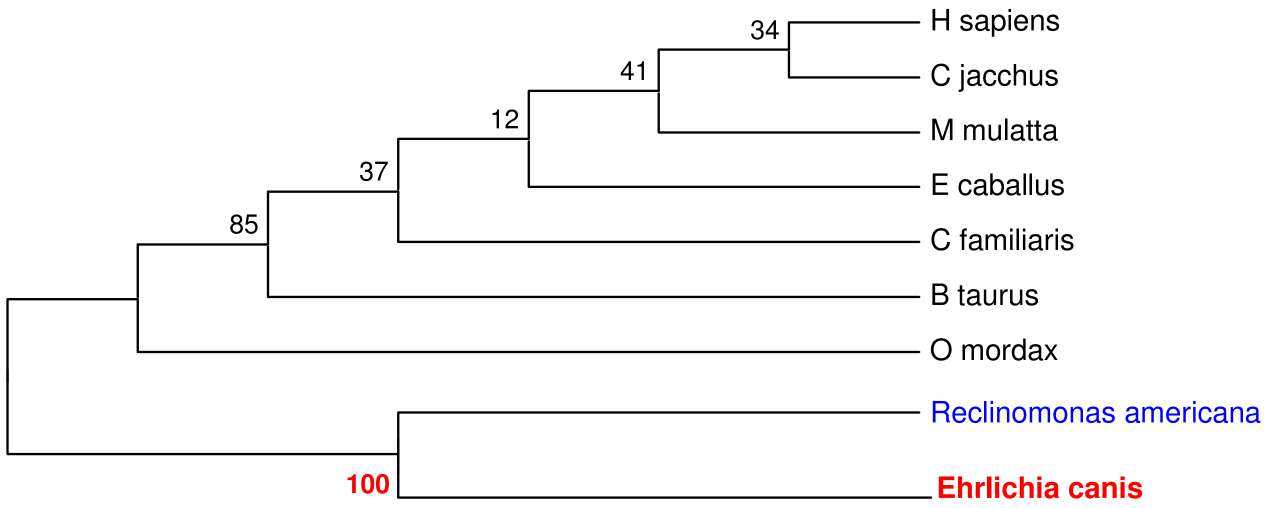


## ABC transporter ATP binding

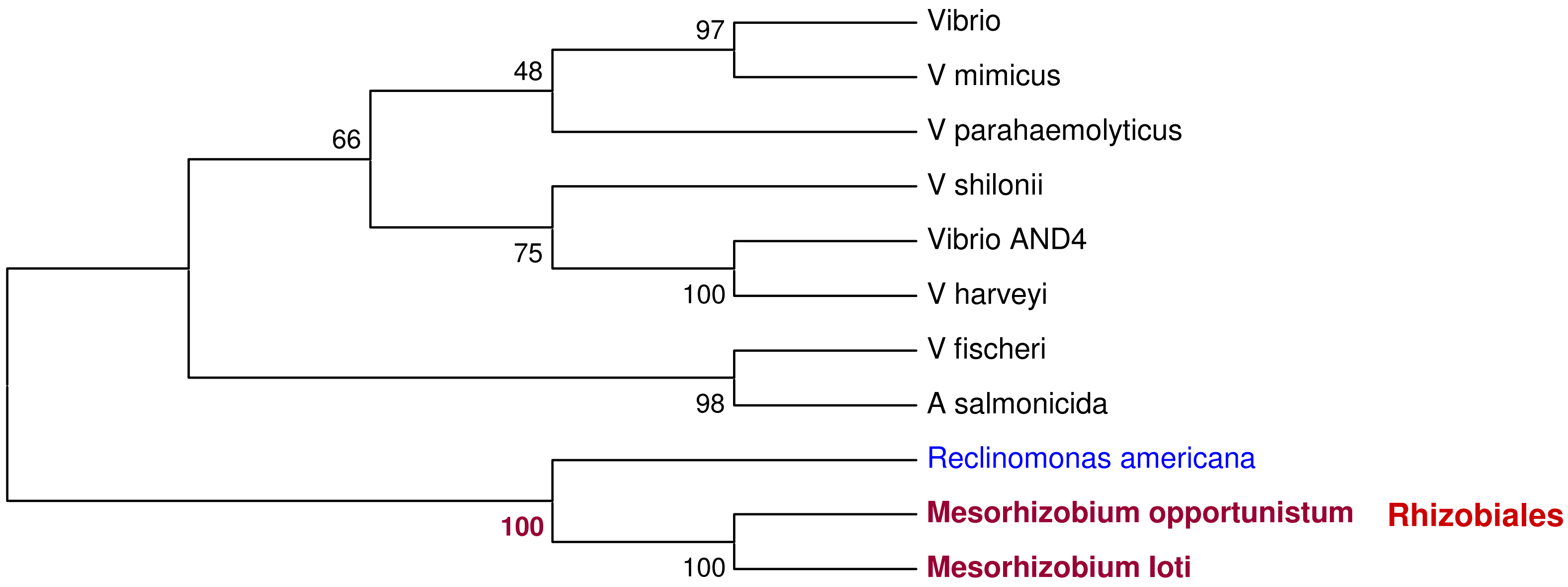

# Ribosomal protein L20

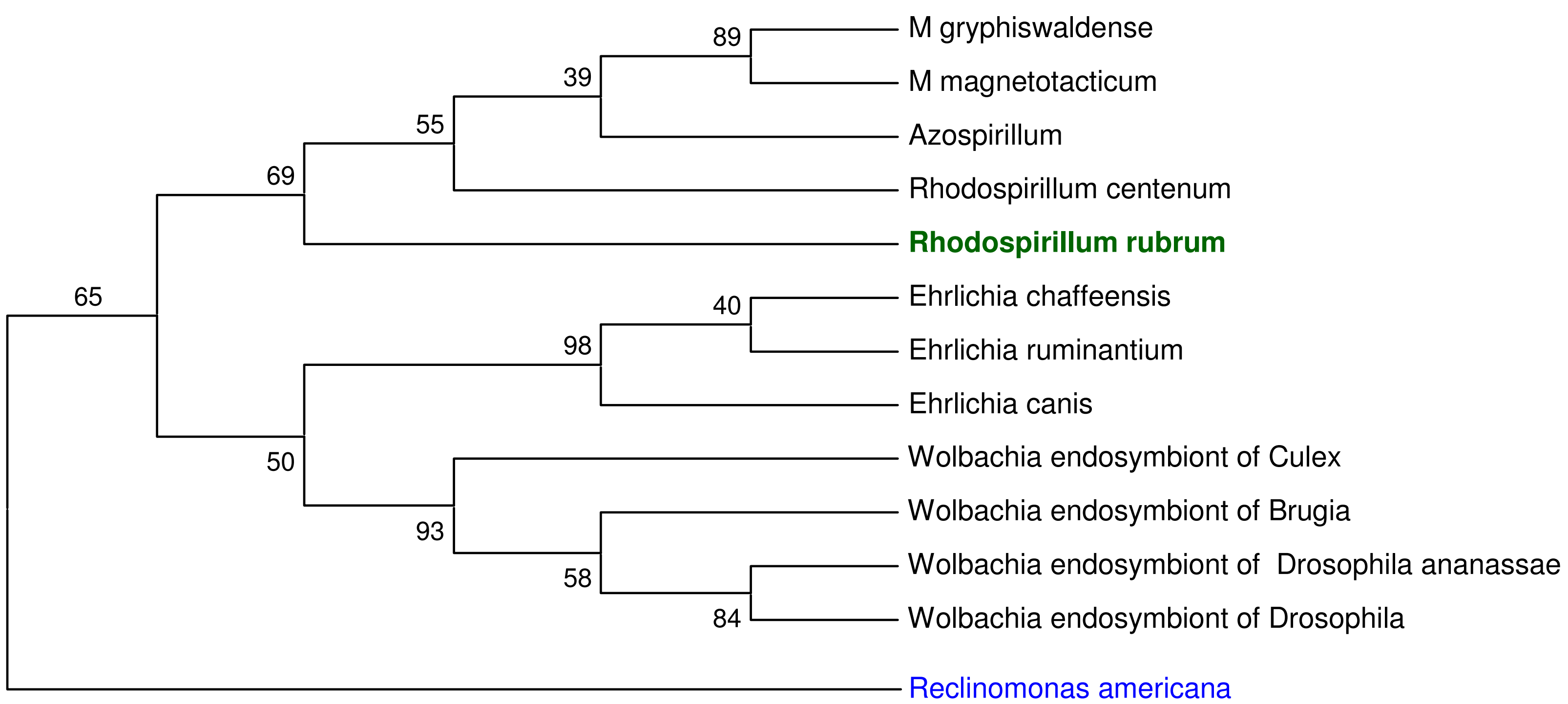

# Ribosomal protein L27

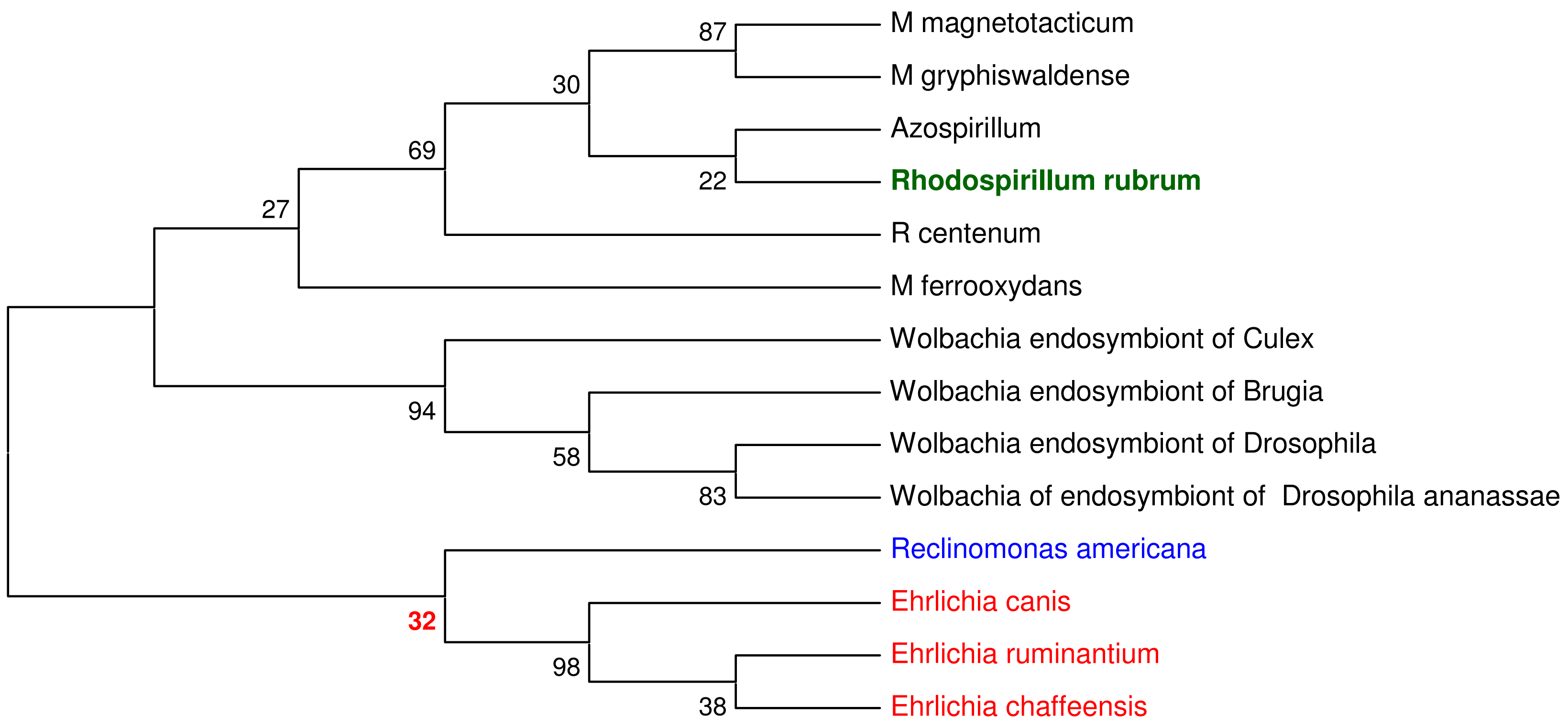

# Ribosomal protein L14

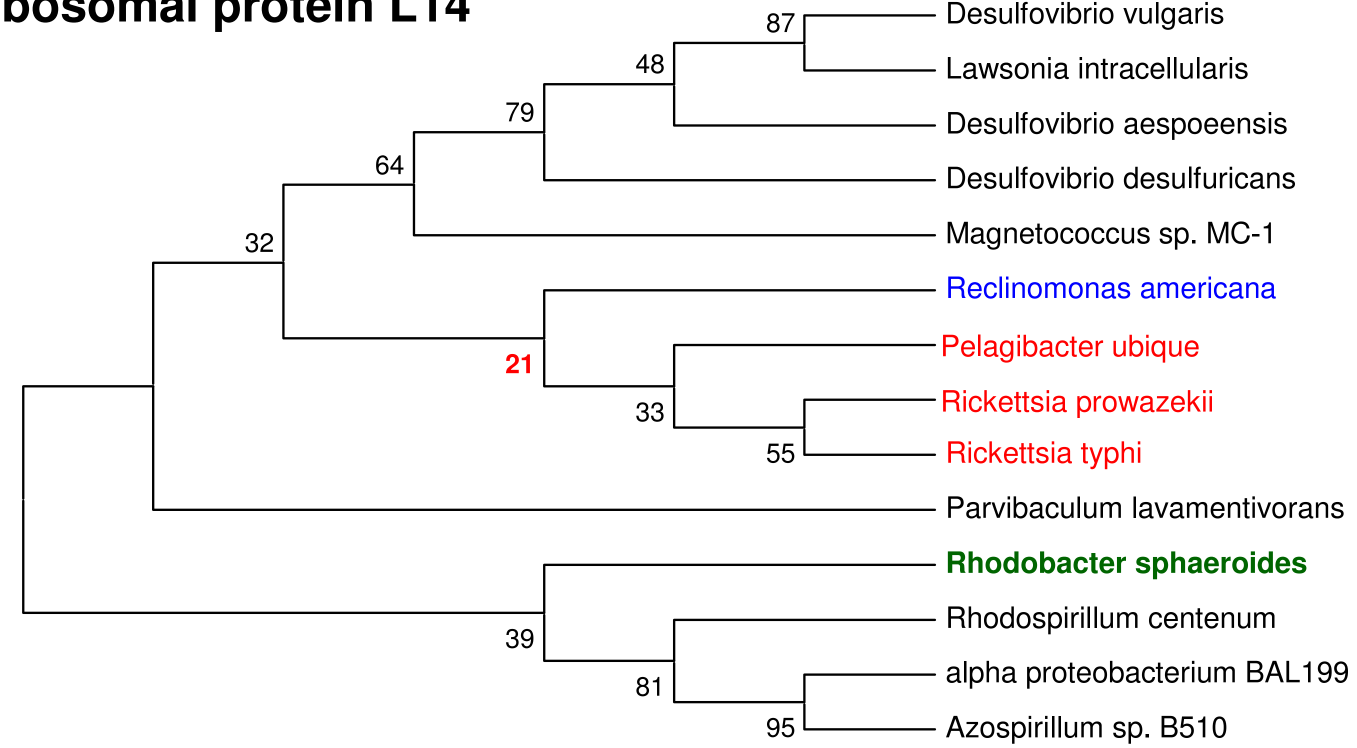

## TatC

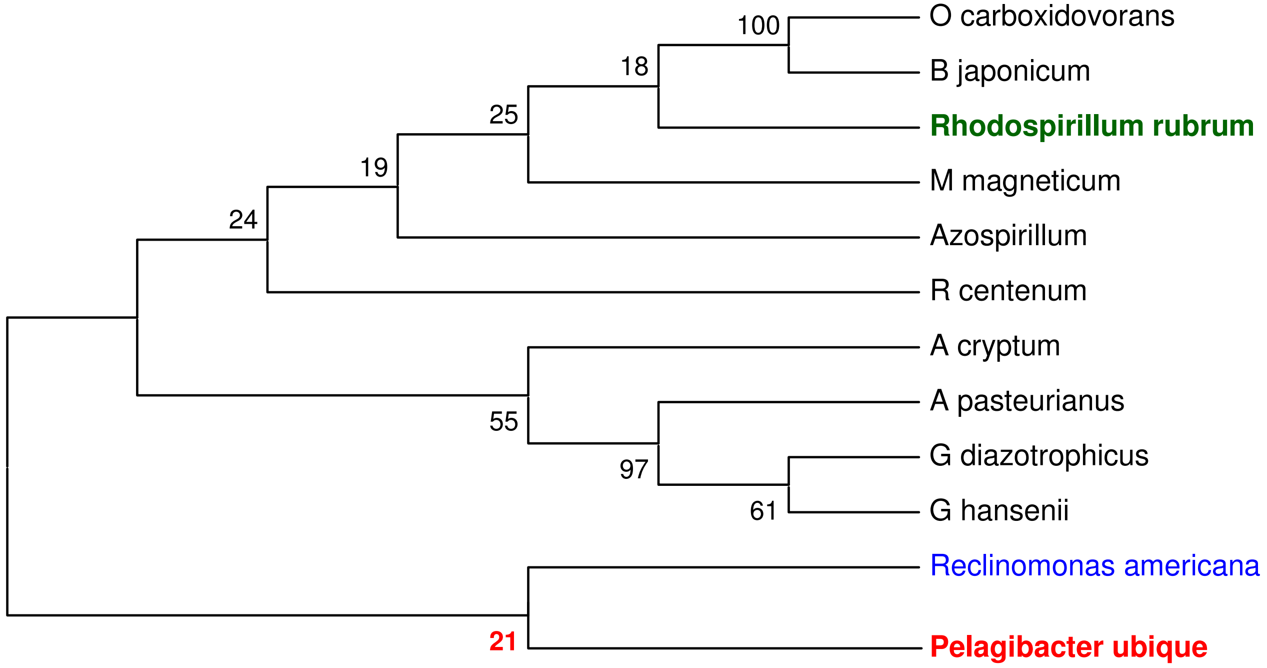
100
18
25
19
24
55
97
61
21
O carboxidovorans
B japonicum
Rhodospirillum rubrum
M magneticum
Azospirillum
R centenum
A cryptum
A pasteurianus
G diazotrophicus
G hansenii
Reclinomonas americana
Pelagibacter ubique

# Ribosomal protein S1

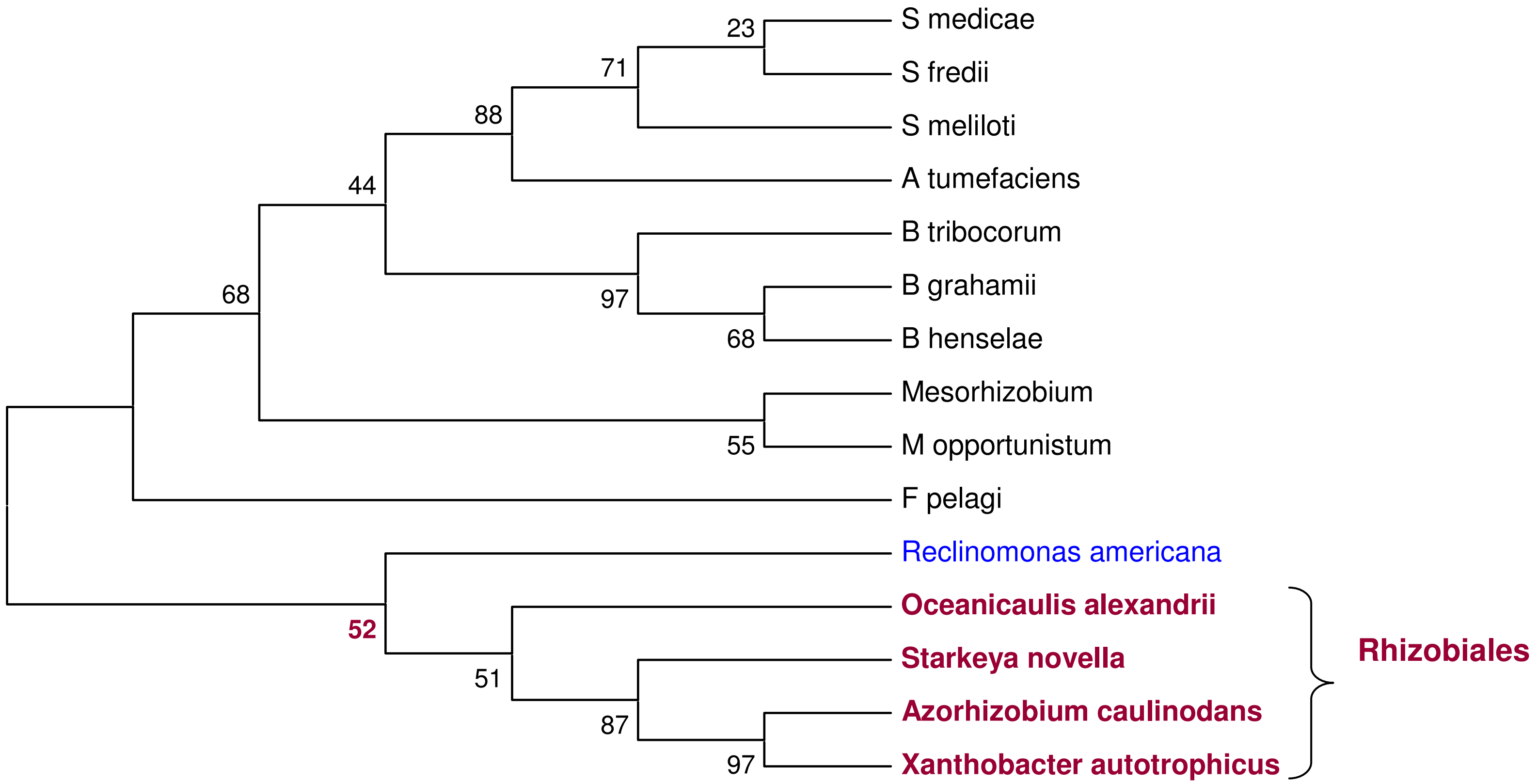

# Ribosomal protein S4

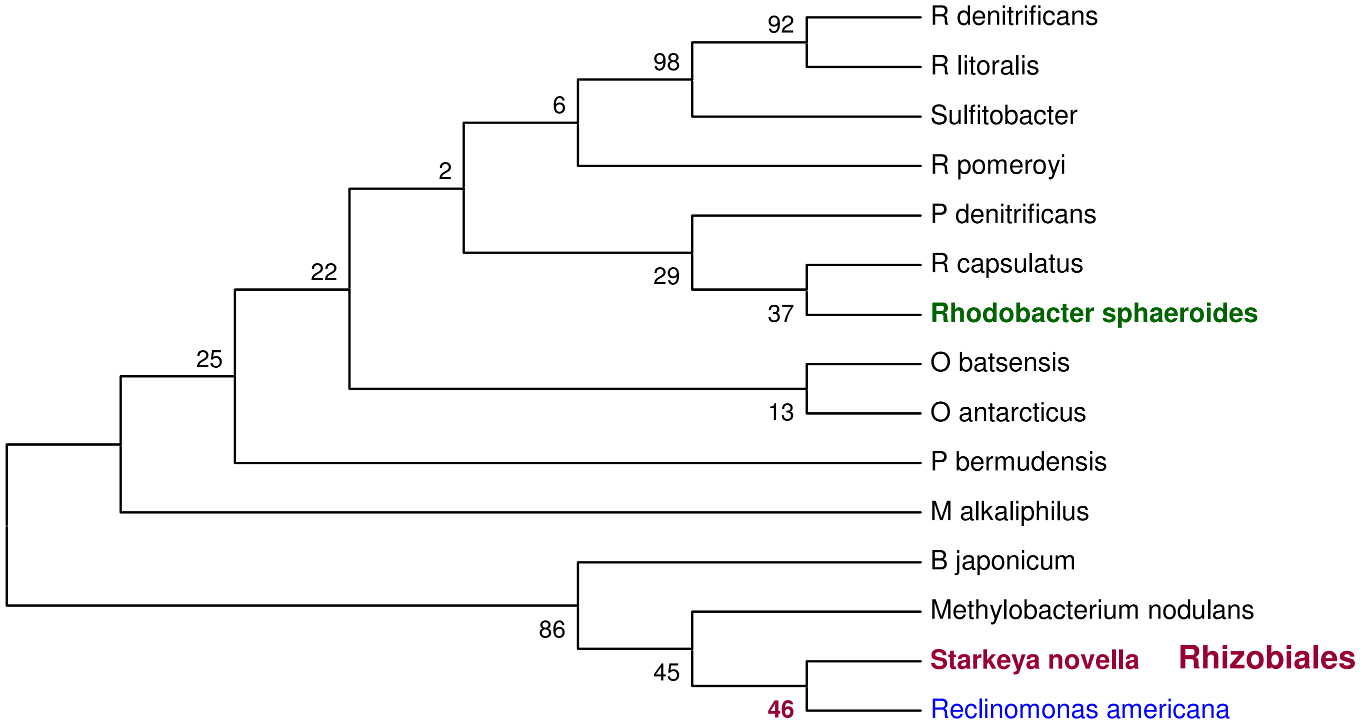

# Ribosomal protein S14

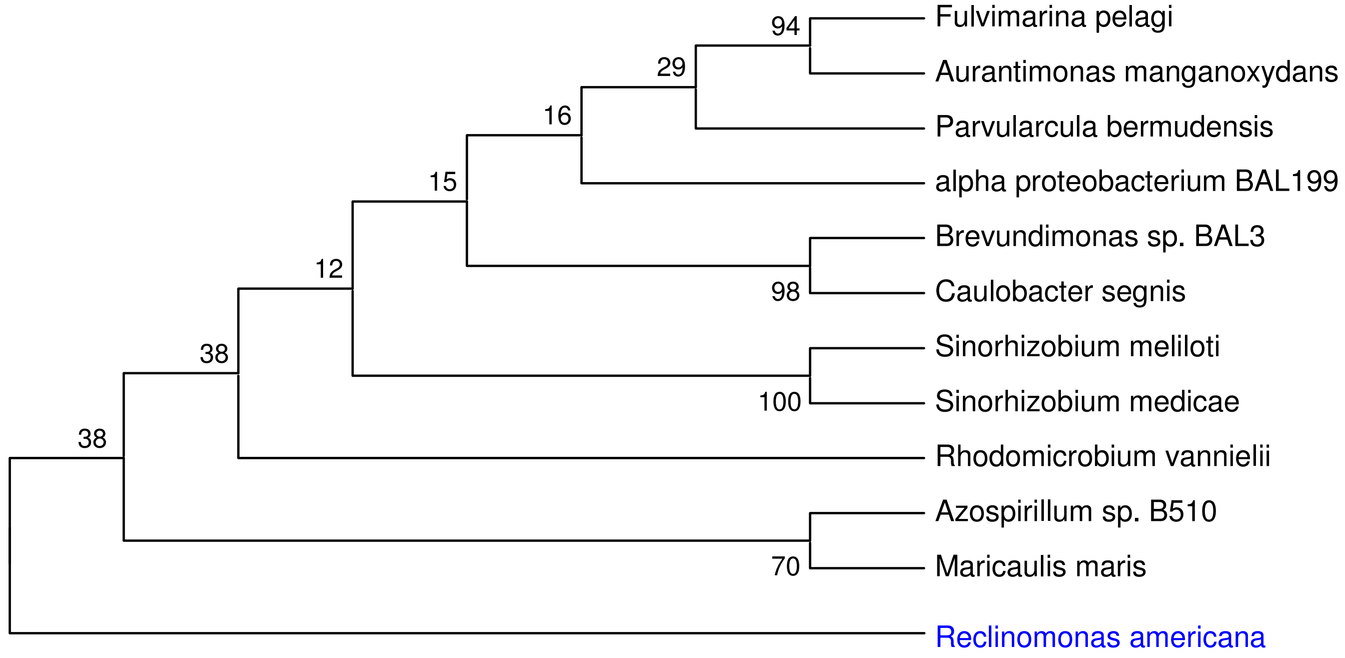

## NADH deshydrogenase su 1

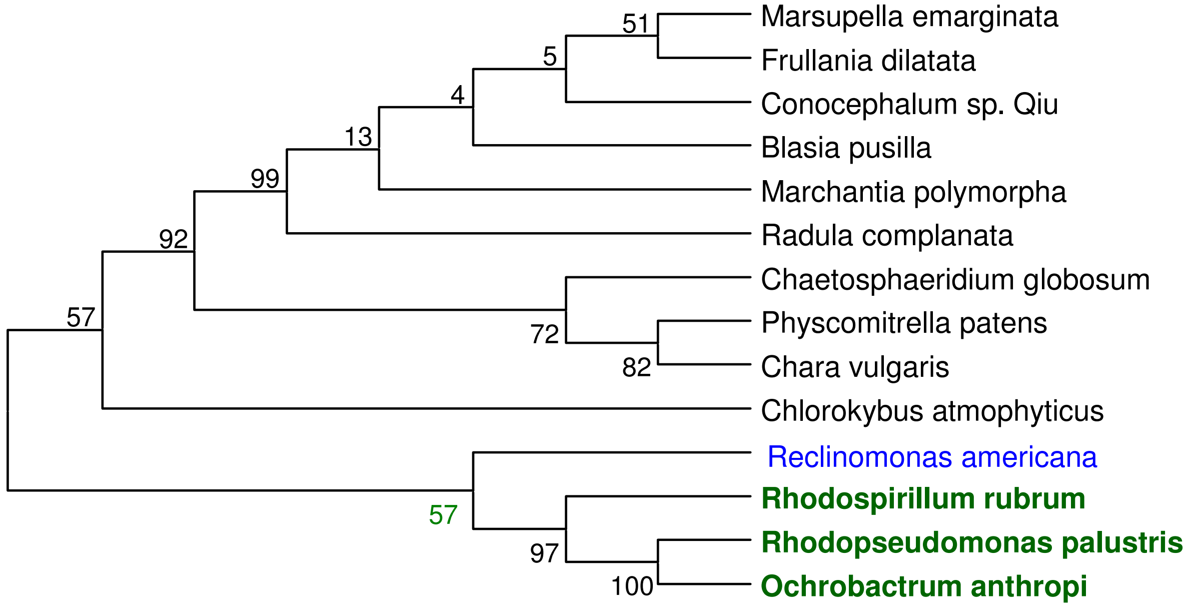


## NADH deshydrogenase su 8

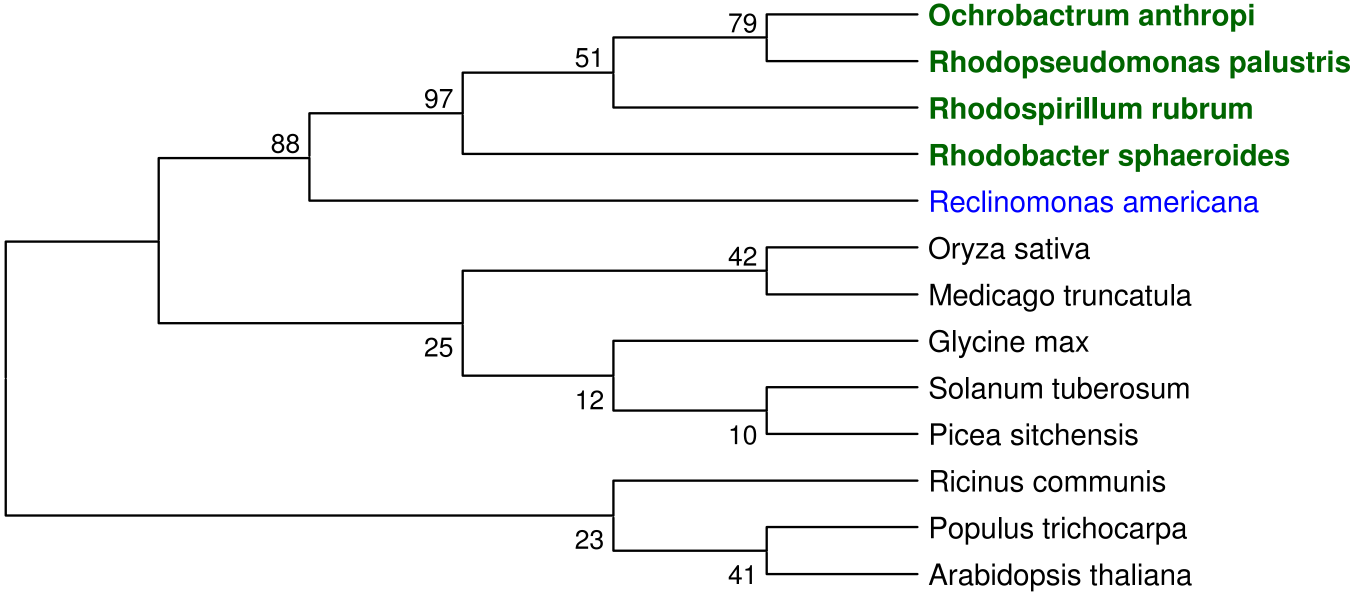

# Haem biosynthesis

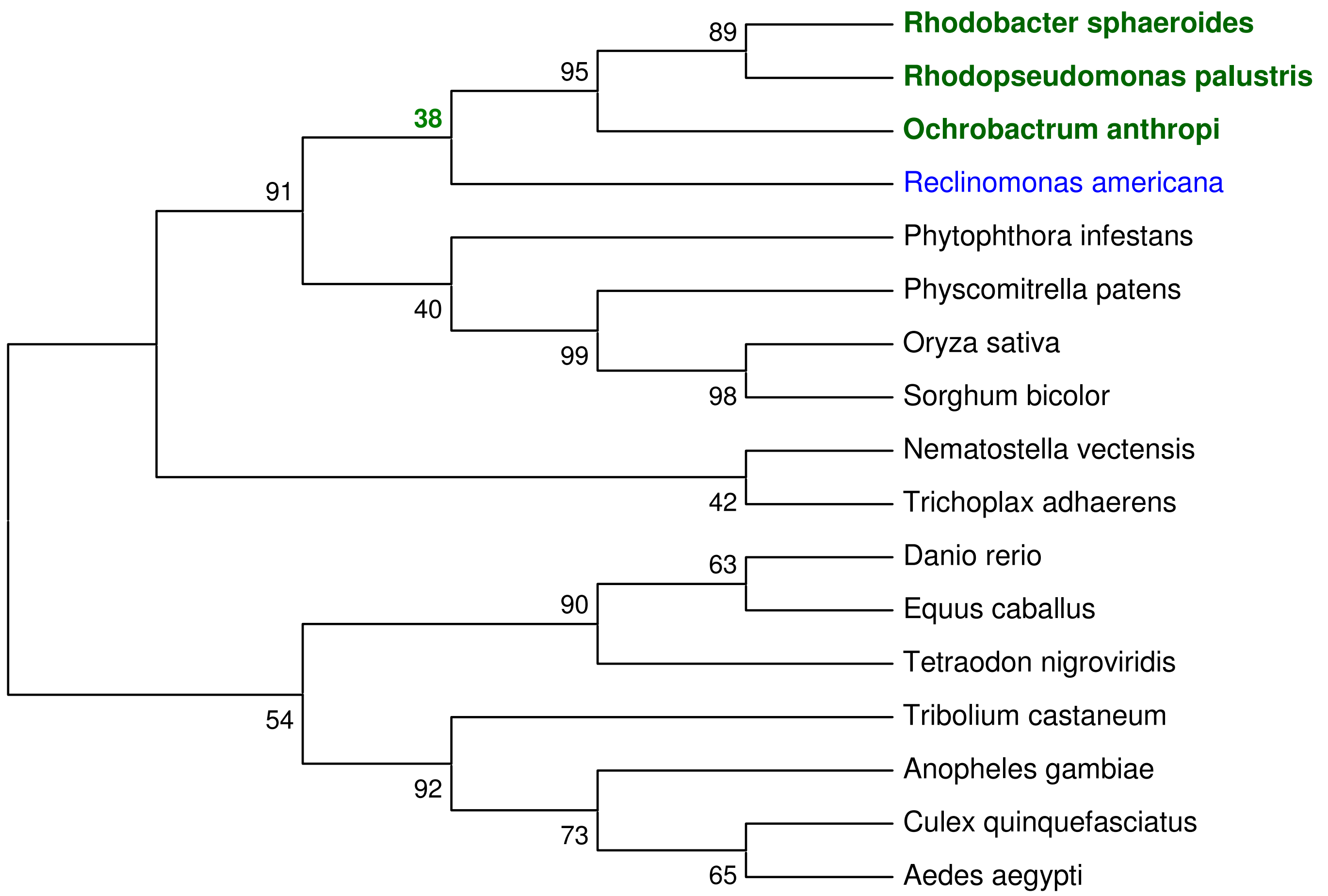

# Ribosomal protein S2

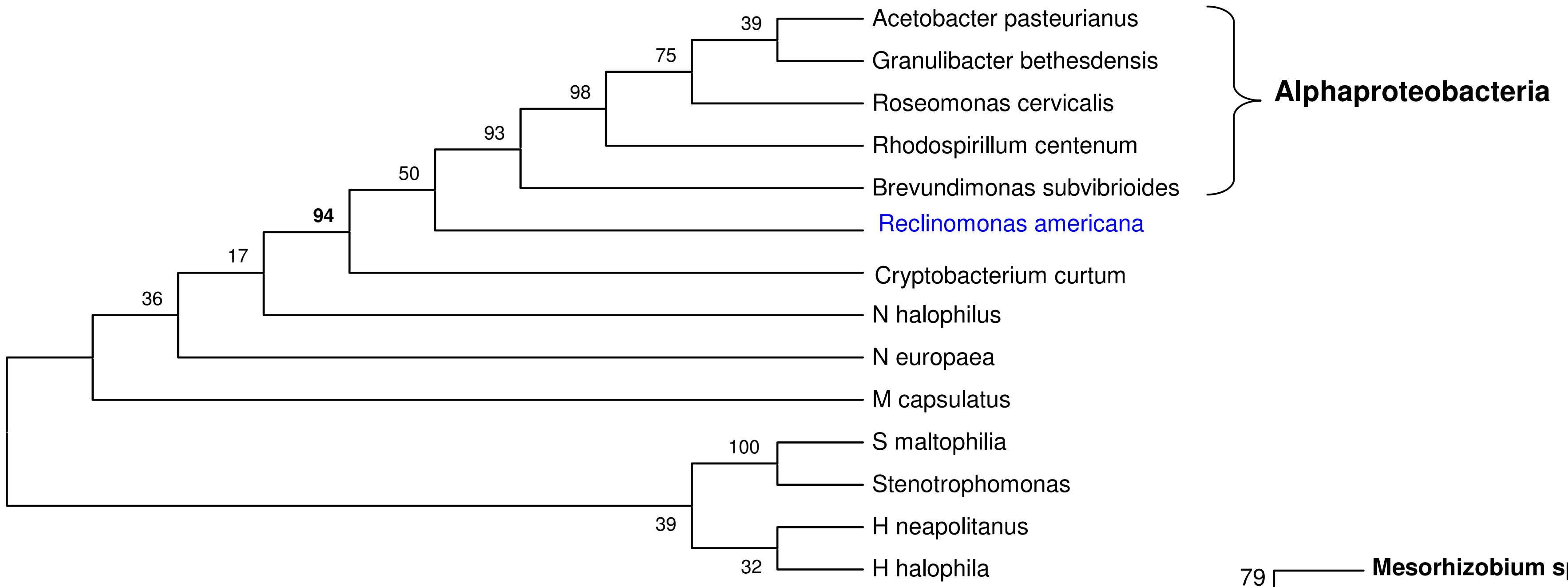


# Ribosomal protein S7

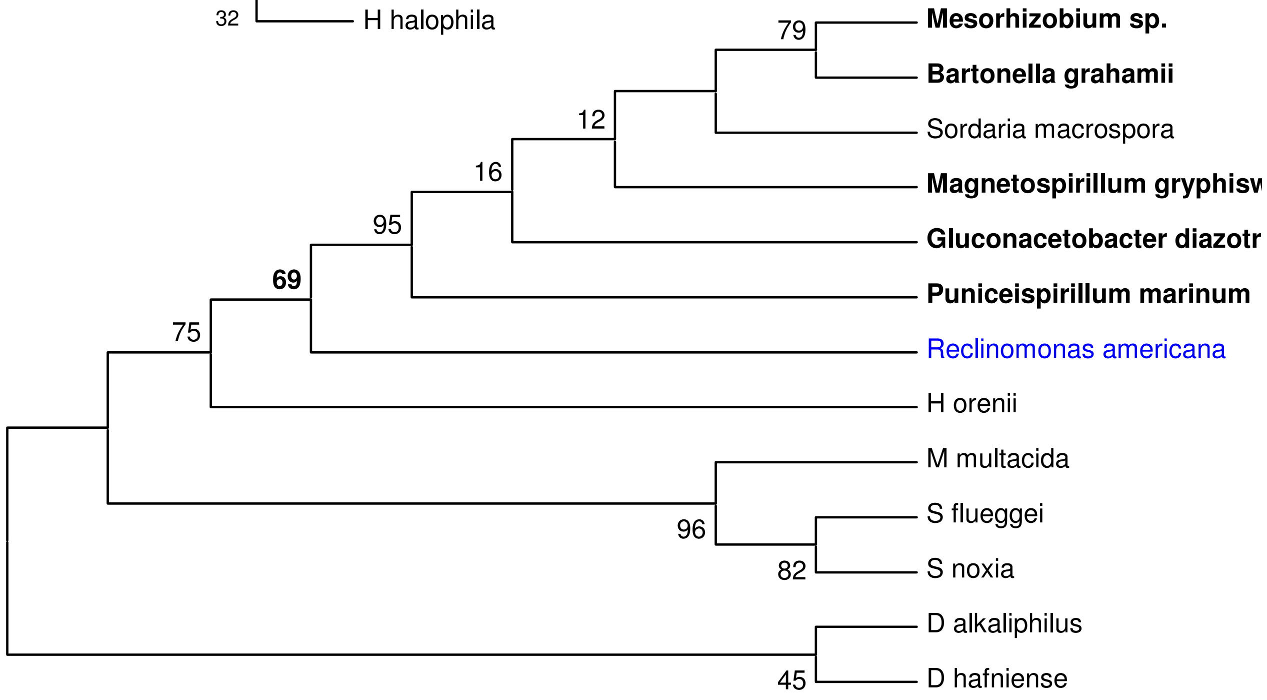

# ABC transporter C subunit

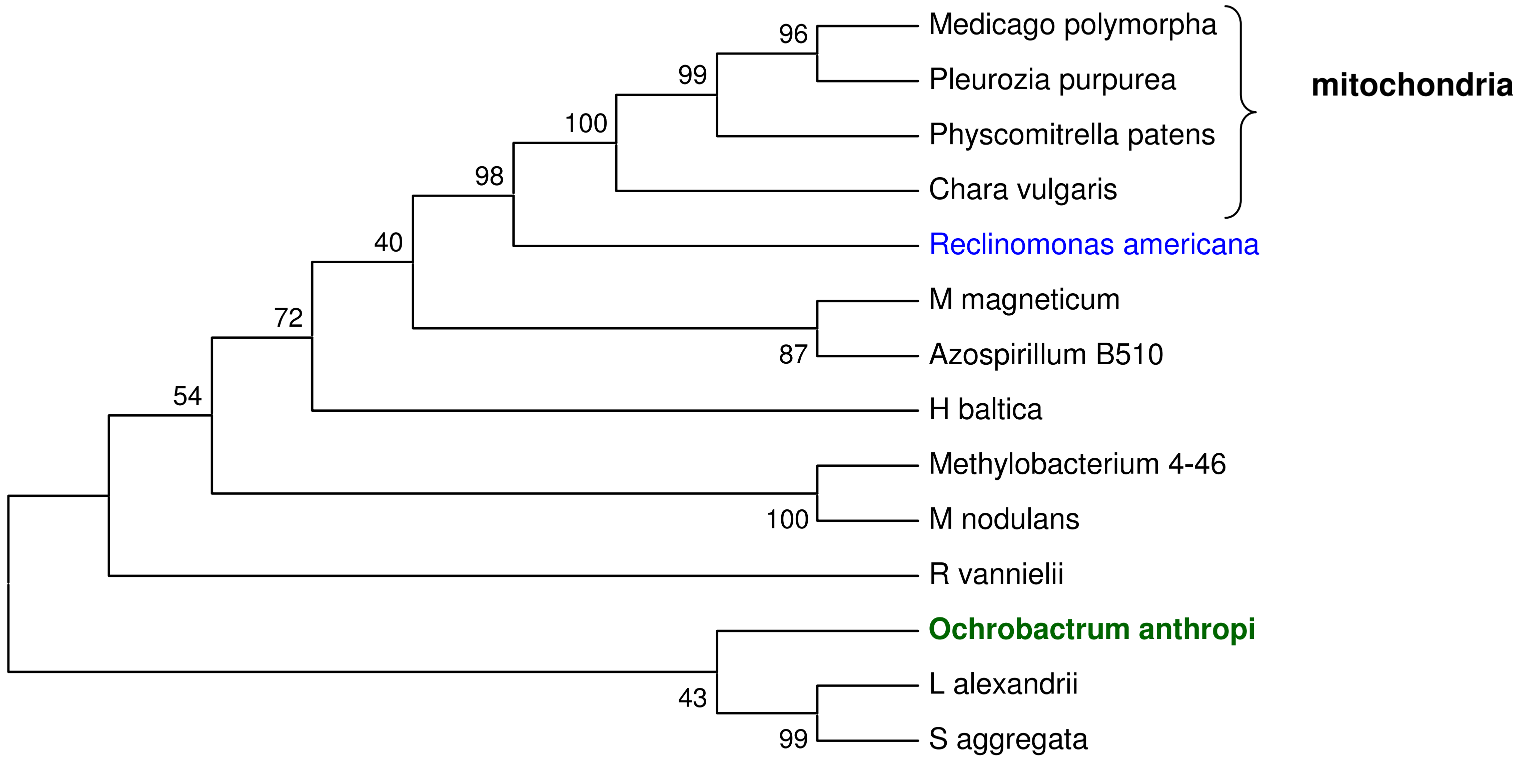

# Ribosomal protein L11

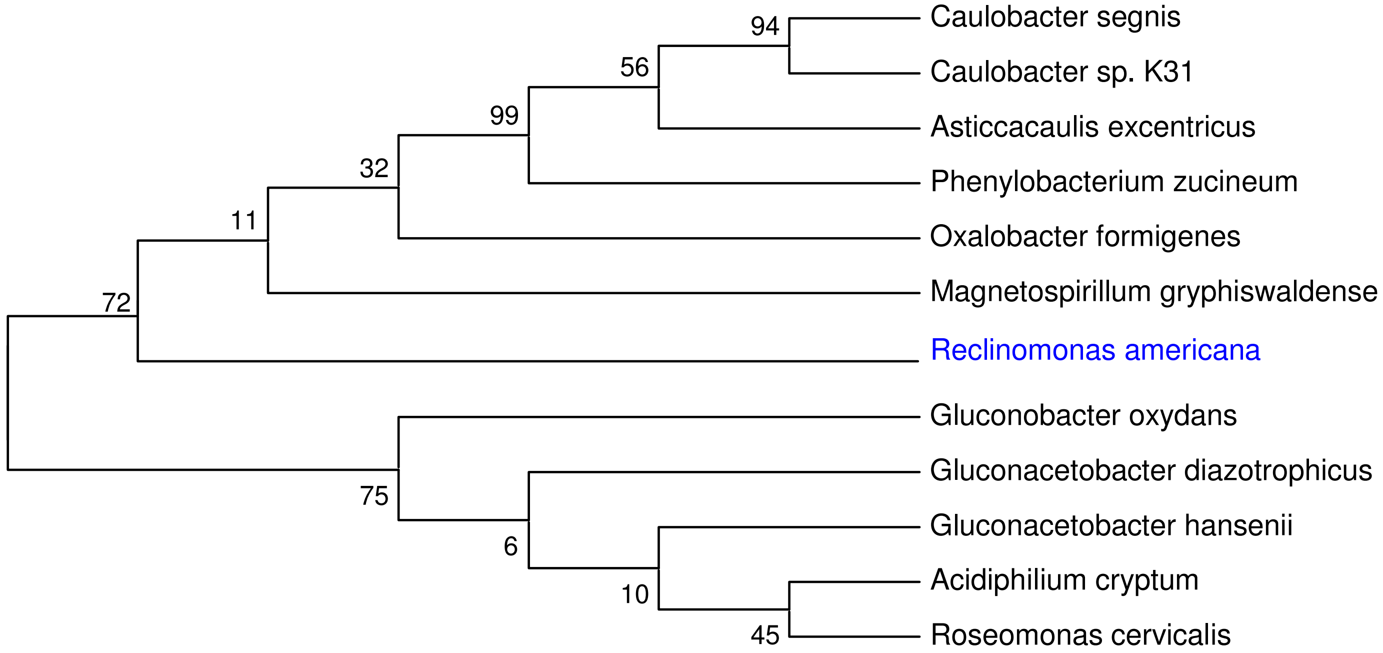

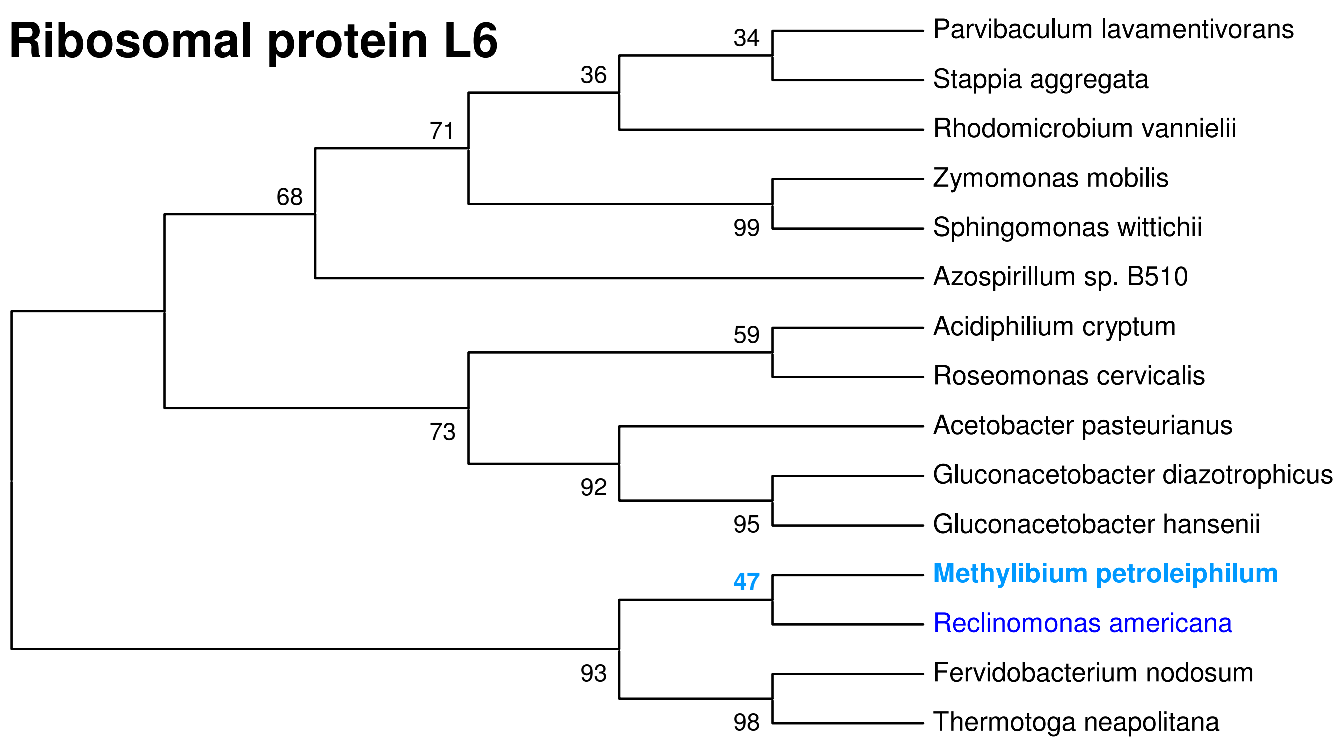
Ribosomal protein L6
34
36
71
68
99
59
73
92
95
47
93
98
Parvibaculum lavamentivorans
Stappia aggregata
Rhodomicrobium vannielii
Zymomonas mobilis
Sphingomonas wittichii
Azospirillum sp. B510
Acidiphilium cryptum
Roseomonas cervicalis
Acetobacter pasteurianus
Gluconacetobacter diazotrophicus
Gluconacetobacter hansenii
Methylibium petroleiphilum
Reclinomonas americana
Fervidobacterium nodosum
Thermotoga neapolitana

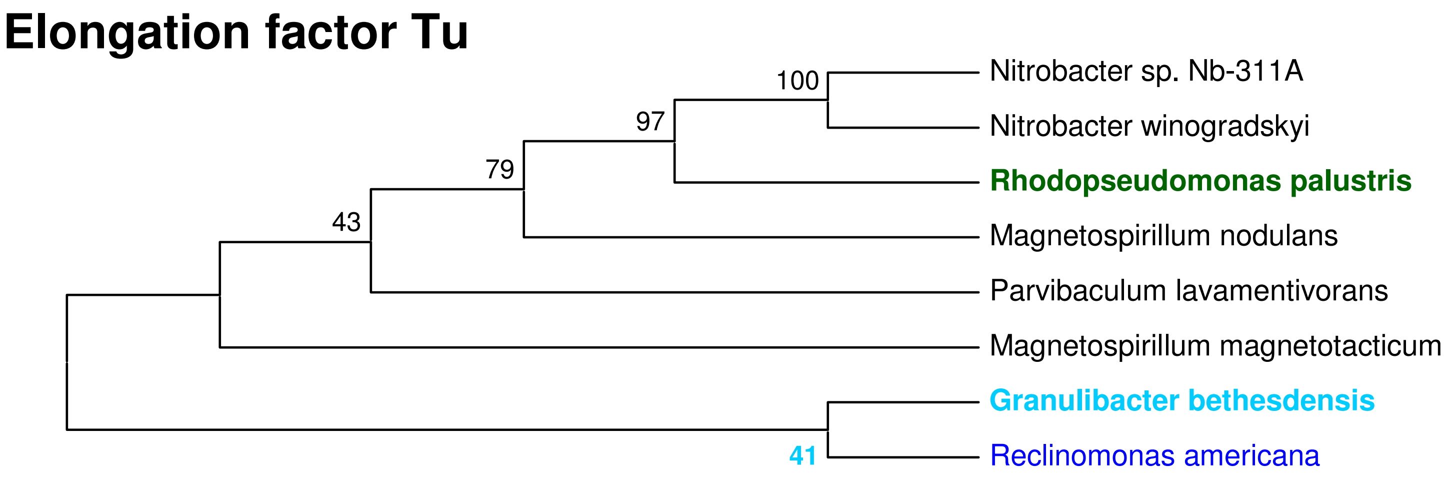
Elongation factor Tu
100
97
79
43
41
Nitrobacter sp. Nb-311A
Nitrobacter winogradskyi
Rhodopseudomonas palustris
Magnetospirillum nodulans
Parvibaculum lavamentivorans
Magnetospirillum magnetotacticum
Granulibacter bethesdensis
Reclinomonas americana

# Ribosomal protein L16

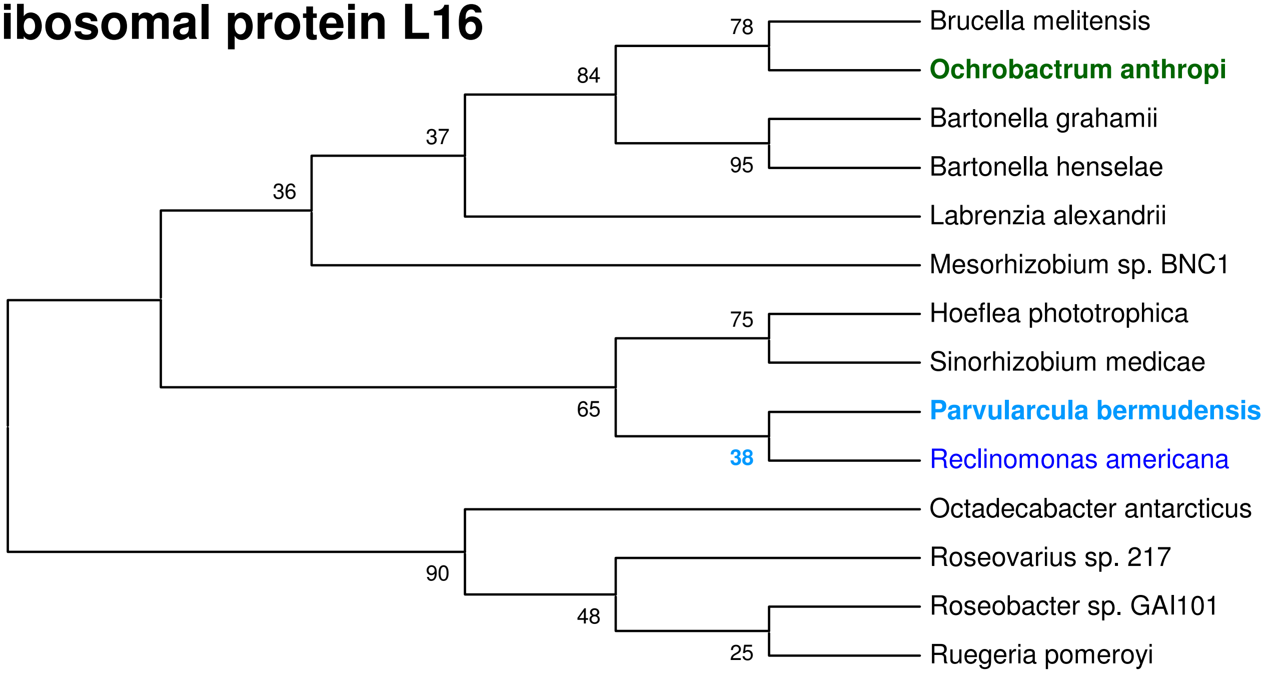

# Ribosomal protein S19

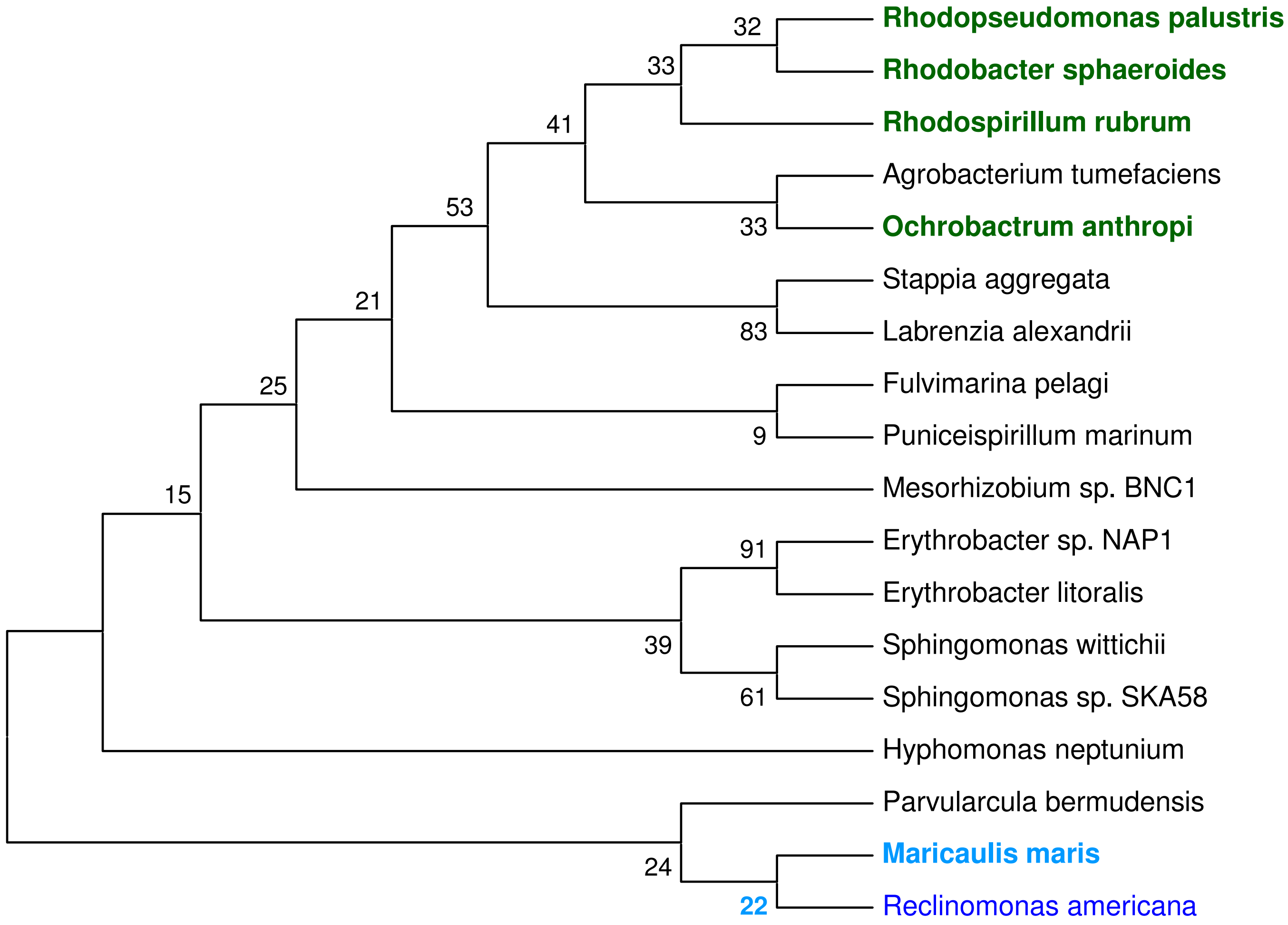

Supplement: Additional file 1 — Reclinomonas americana mitochondrial phylogenies. [file 1745-6150-6-55-S1.PDF]
