# Supplementary material for: The rhizome of Reclinomonas americana, Homo sapiens, Pediculus humanus and Saccharomyces cerevisiae mitochondria
Source: Biol Direct. 2011 Oct 20;6:55. doi: 10.1186/1745-6150-6-55 (PMC3214132; doi:10.1186/1745-6150-6-55)

NADH deshydrogenase su 6

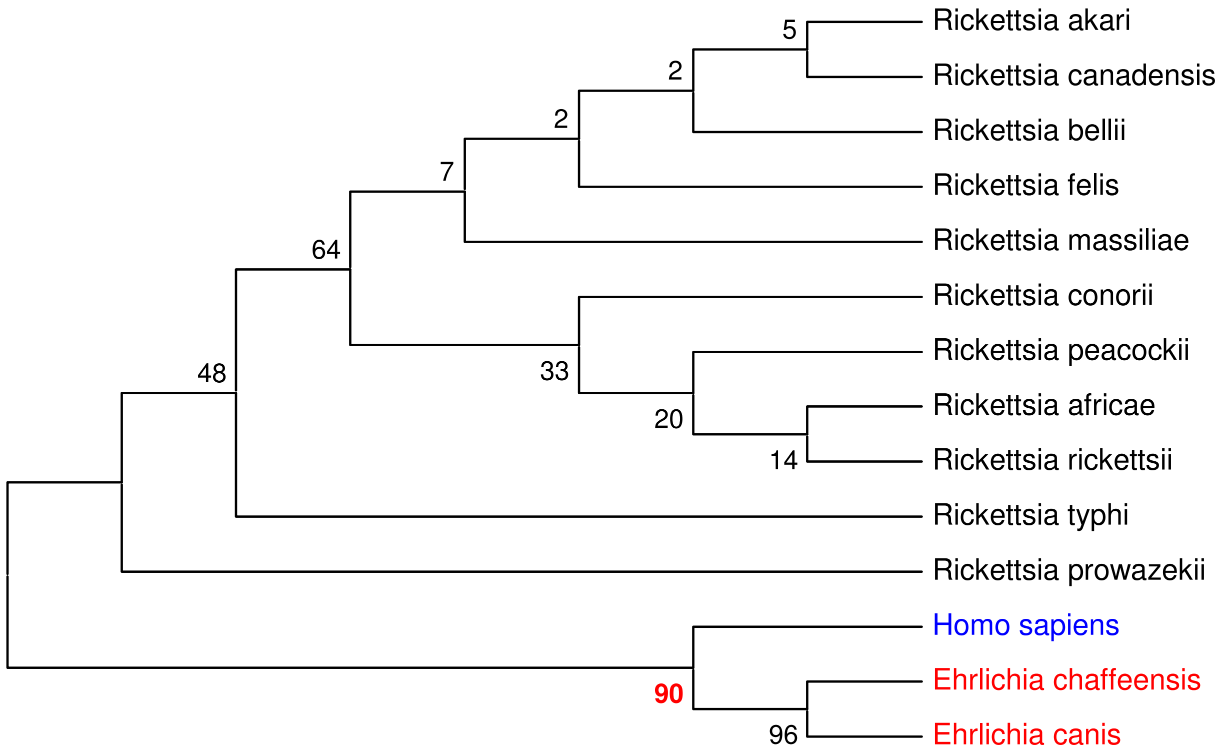

Cytochrome c oxidase I

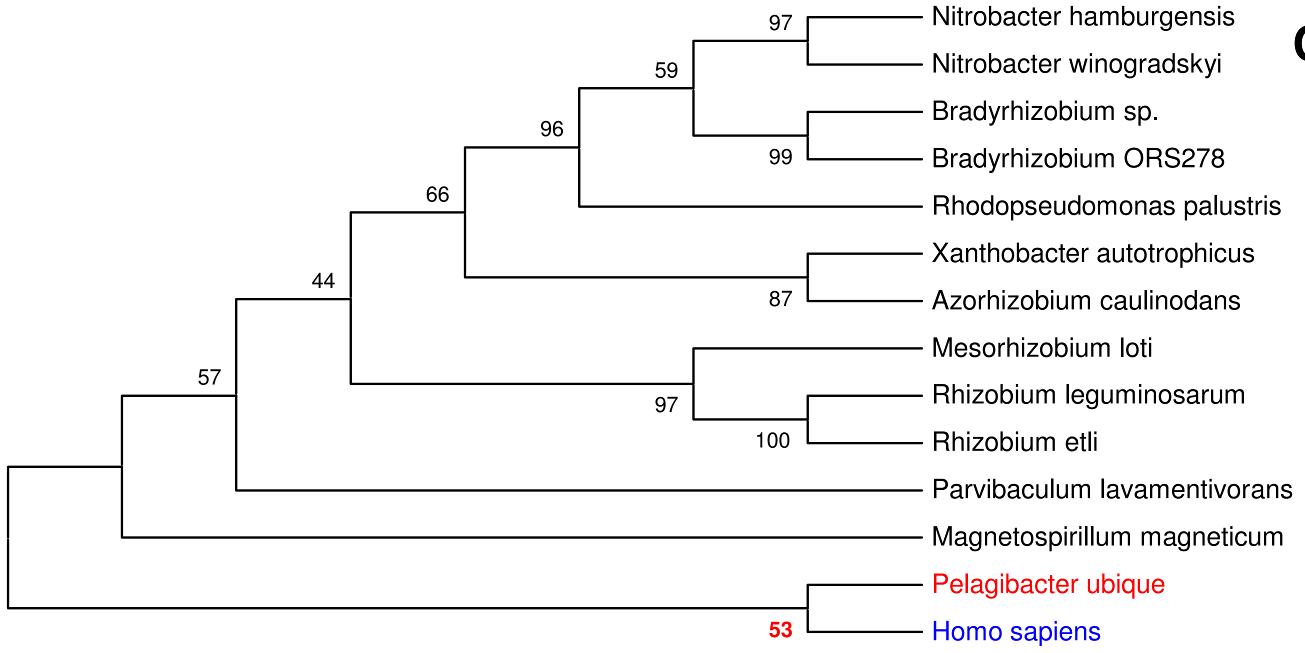

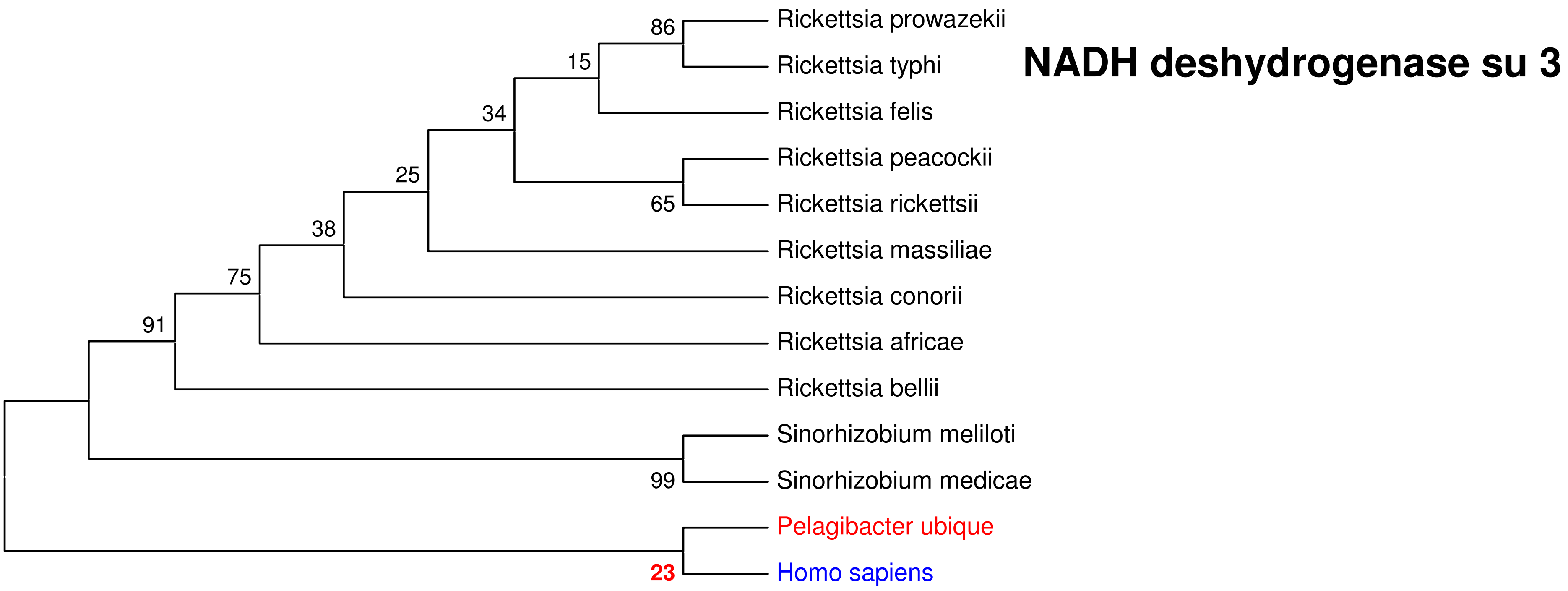
NADH deshydrogenase su 3
Rickettsia prowazekii
Rickettsia typhi
Rickettsia felis
Rickettsia peacockii
Rickettsia rickettsii
Rickettsia massiliae
Rickettsia conorii
Rickettsia africae
Rickettsia bellii
Sinorhizobium meliloti
Sinorhizobium medicae
Pelagibacter ubique
Homo sapiens
86
15
34
65
25
38
75
91
99
23

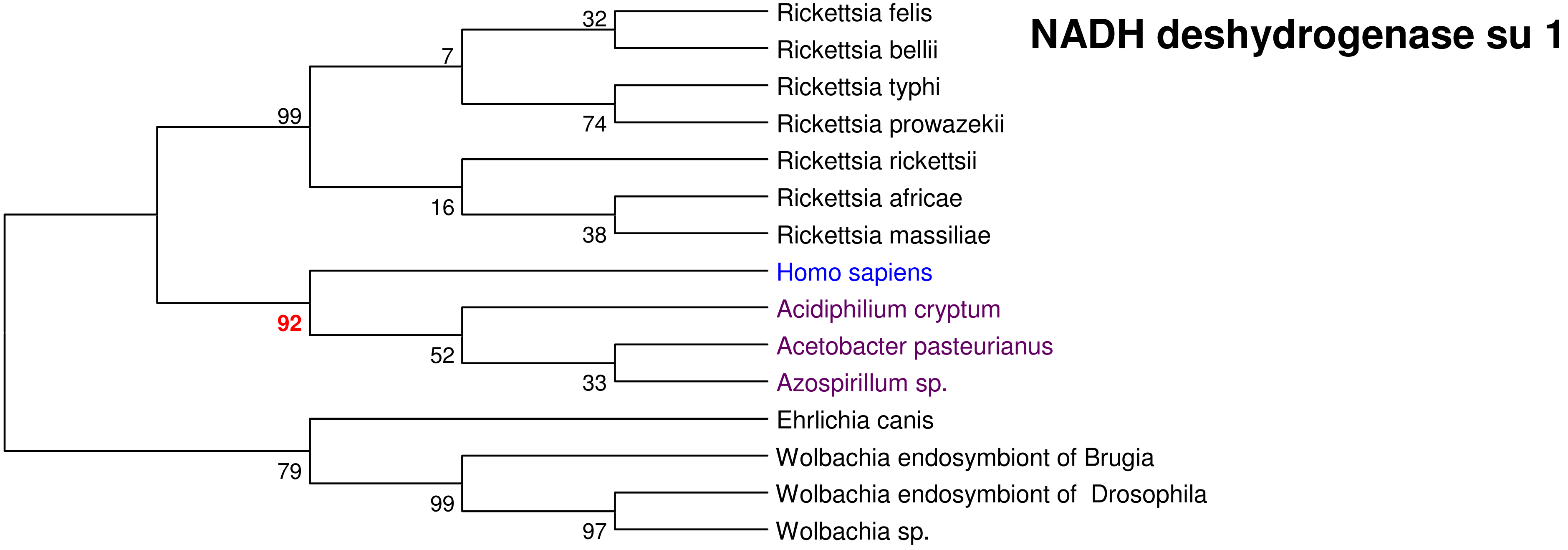
NADH deshydrogenase su 1
Rickettsia felis
Rickettsia bellii
Rickettsia typhi
Rickettsia prowazekii
Rickettsia rickettsii
Rickettsia africae
Rickettsia massiliae
Homo sapiens
Acidiphilium cryptum
Acetobacter pasteurianus
Azospirillum sp.
Ehrlichia canis
Wolbachia endosymbiont of Brugia
Wolbachia endosymbiont of Drosophila
Wolbachia sp.
32
7
99
74
16
38
92
52
33
79
99
97

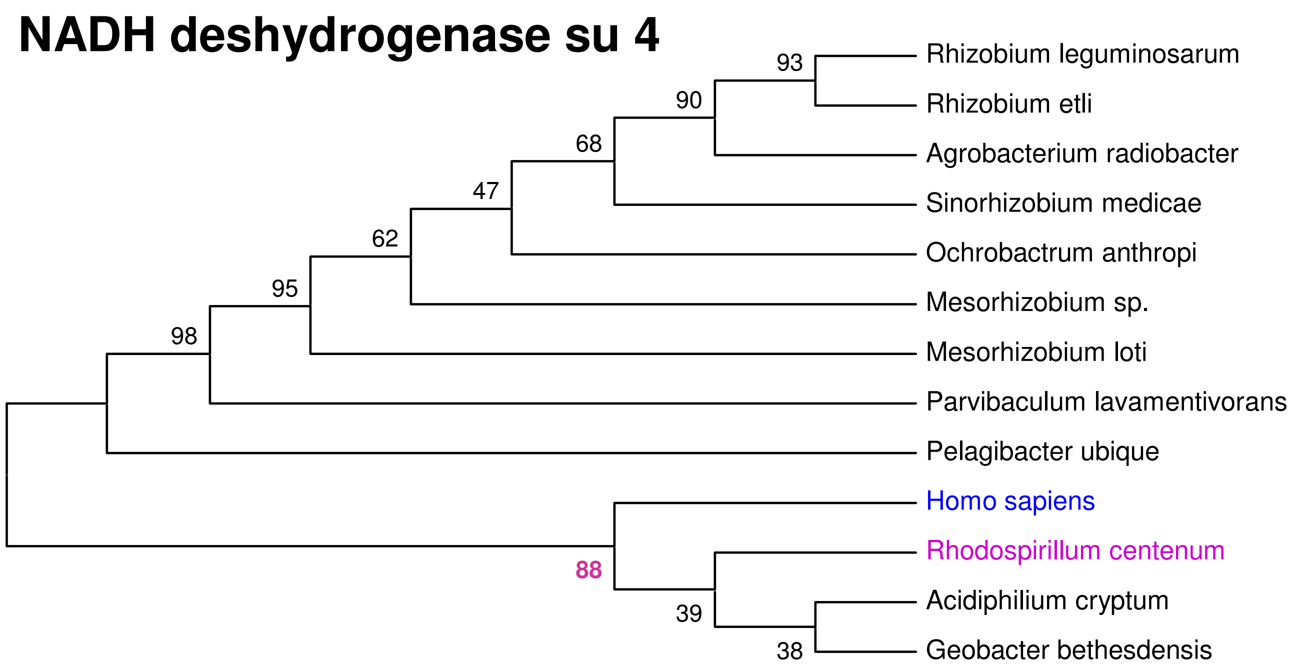
NADH deshydrogenase su 4
93
90
68
47
62
95
98
88
39
38
Rhizobium leguminosarum
Rhizobium etli
Agrobacterium radiobacter
Sinorhizobium medicae
Ochrobactrum anthropi
Mesorhizobium sp.
Mesorhizobium loti
Parvibaculum lavamentivorans
Pelagibacter ubique
Homo sapiens
Rhodospirillum centenum
Acidiphilium cryptum
Geobacter bethesdensis

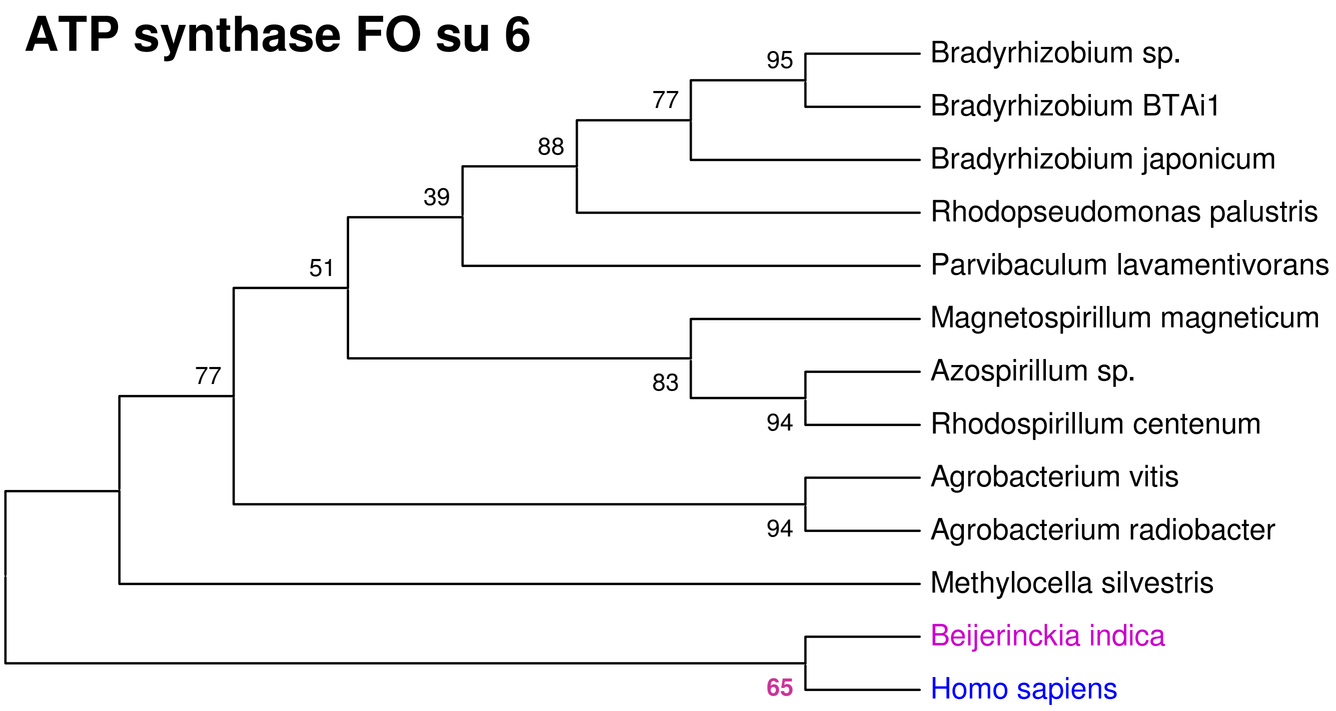
ATP synthase FO su 6
95
77
88
39
51
83
94
77
94
65
Bradyrhizobium sp.
Bradyrhizobium BTAi1
Bradyrhizobium japonicum
Rhodopseudomonas palustris
Parvibaculum lavamentivorans
Magnetospirillum magneticum
Azospirillum sp.
Rhodospirillum centenum
Agrobacterium vitis
Agrobacterium radiobacter
Methylocella silvestris
Beijerinckia indica
Homo sapiens

ATP synthase FO su 8

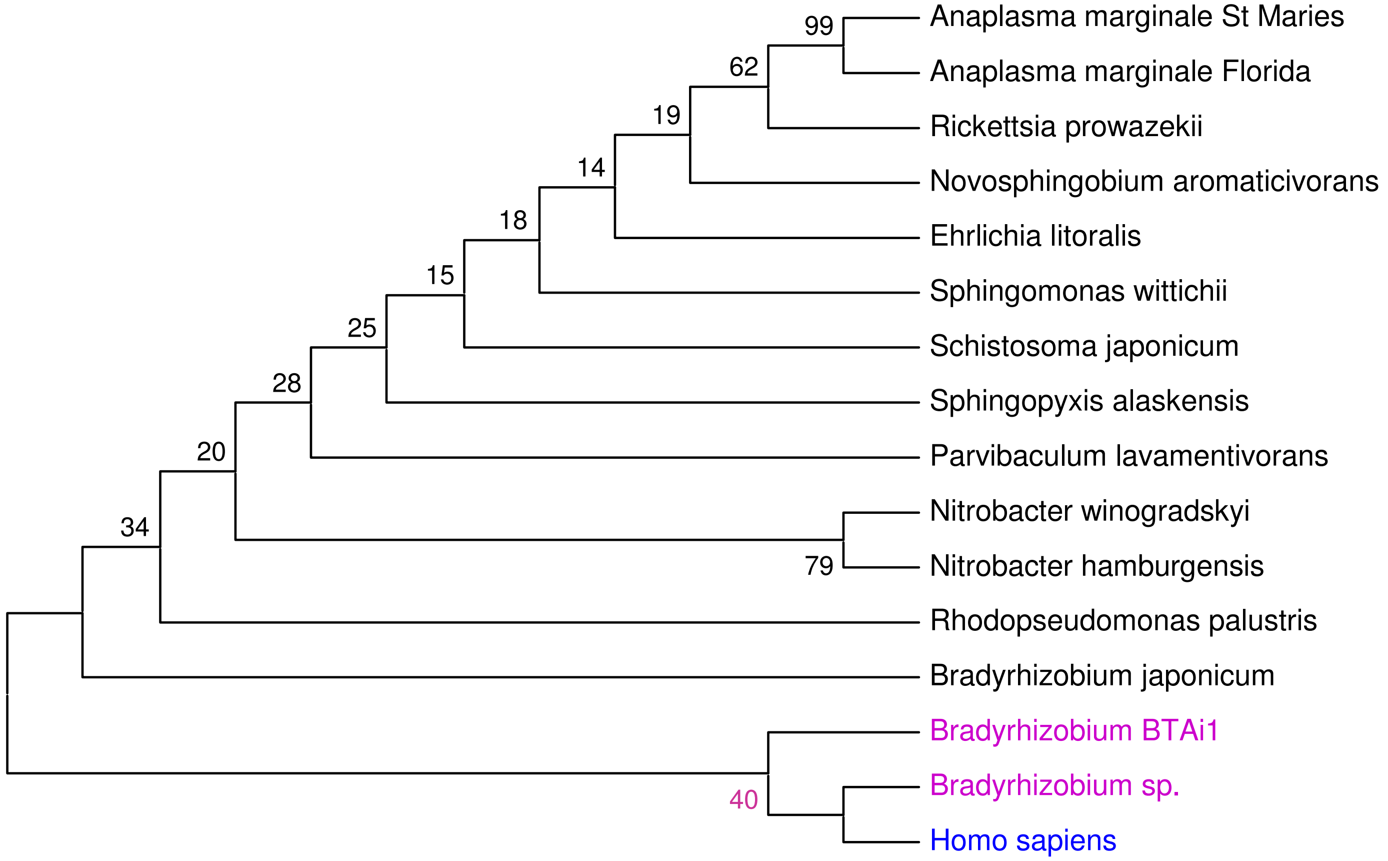

NADH deshydrogenase su 4L

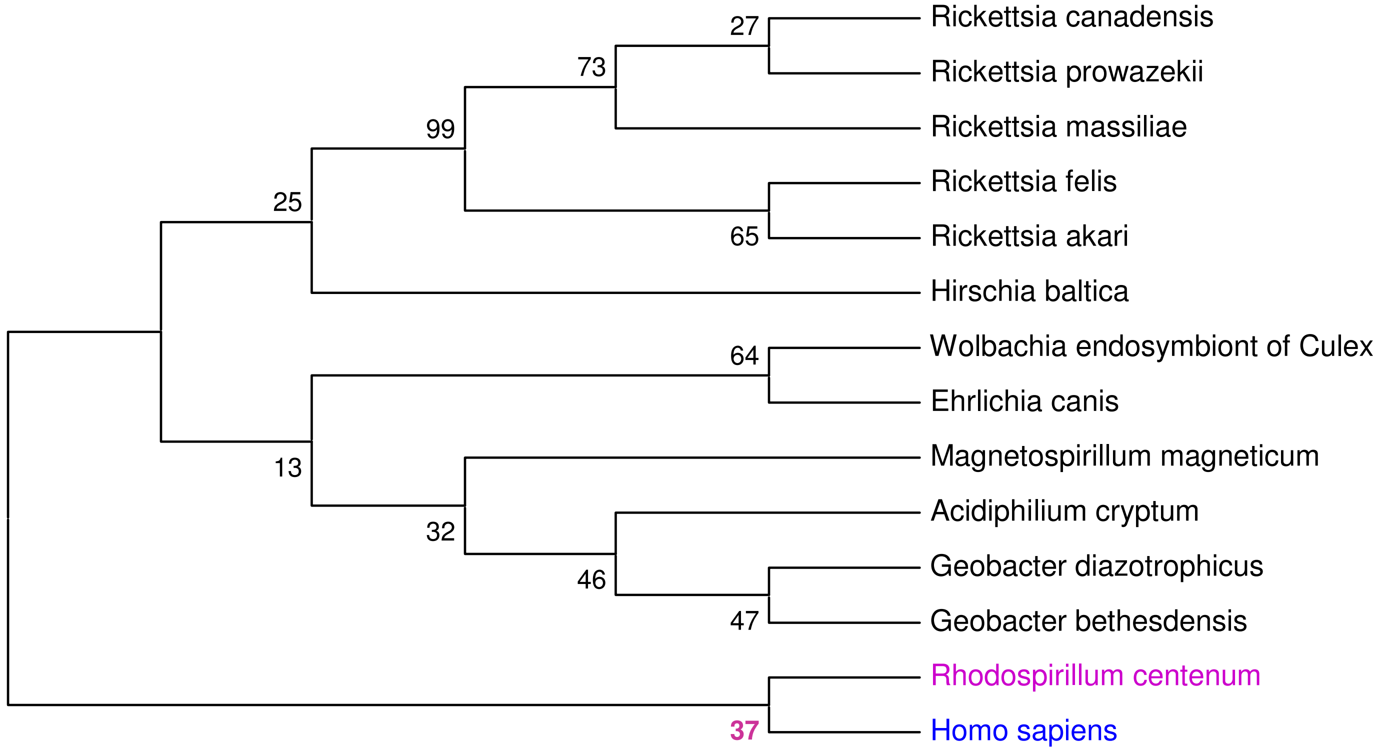

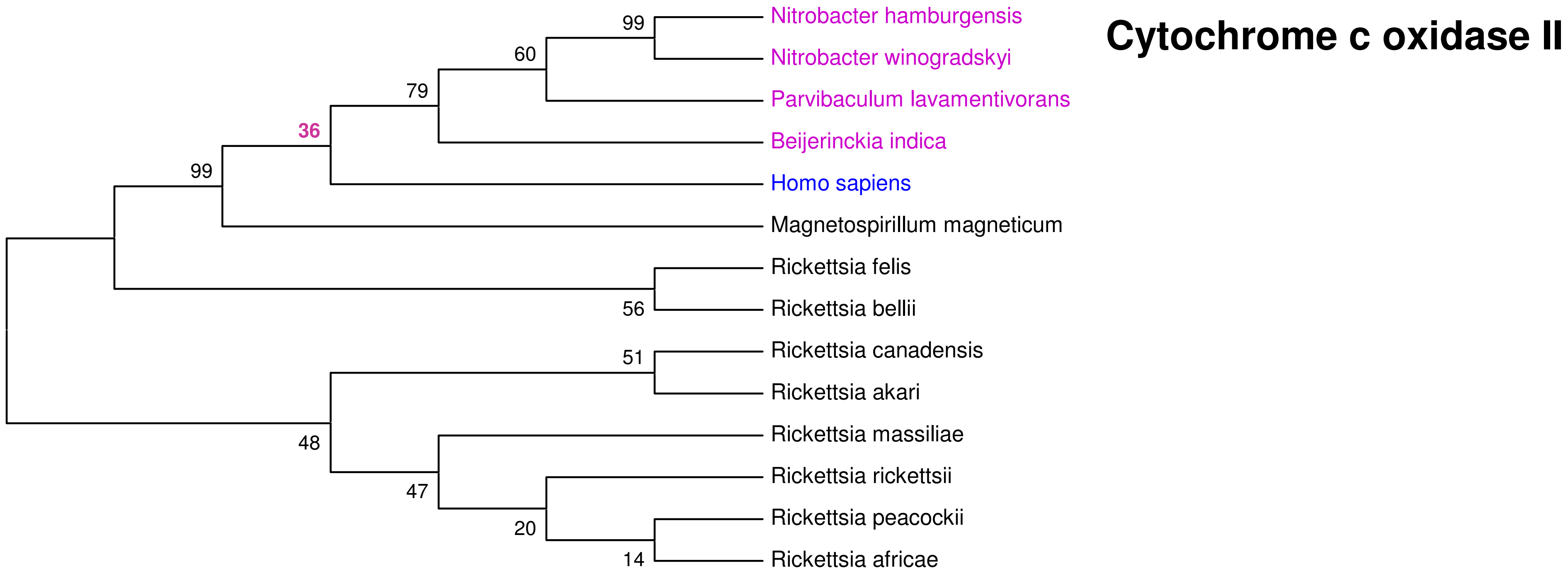
Cytochrome c oxidase II
99
60
79
36
99
56
51
48
47
20
14
Nitrobacter hamburgensis
Nitrobacter winogradskyi
Parvibaculum lavamentivorans
Beijerinckia indica
Homo sapiens
Magnetospirillum magneticum
Rickettsia felis
Rickettsia bellii
Rickettsia canadensis
Rickettsia akari
Rickettsia massiliae
Rickettsia rickettsii
Rickettsia peacockii
Rickettsia africae

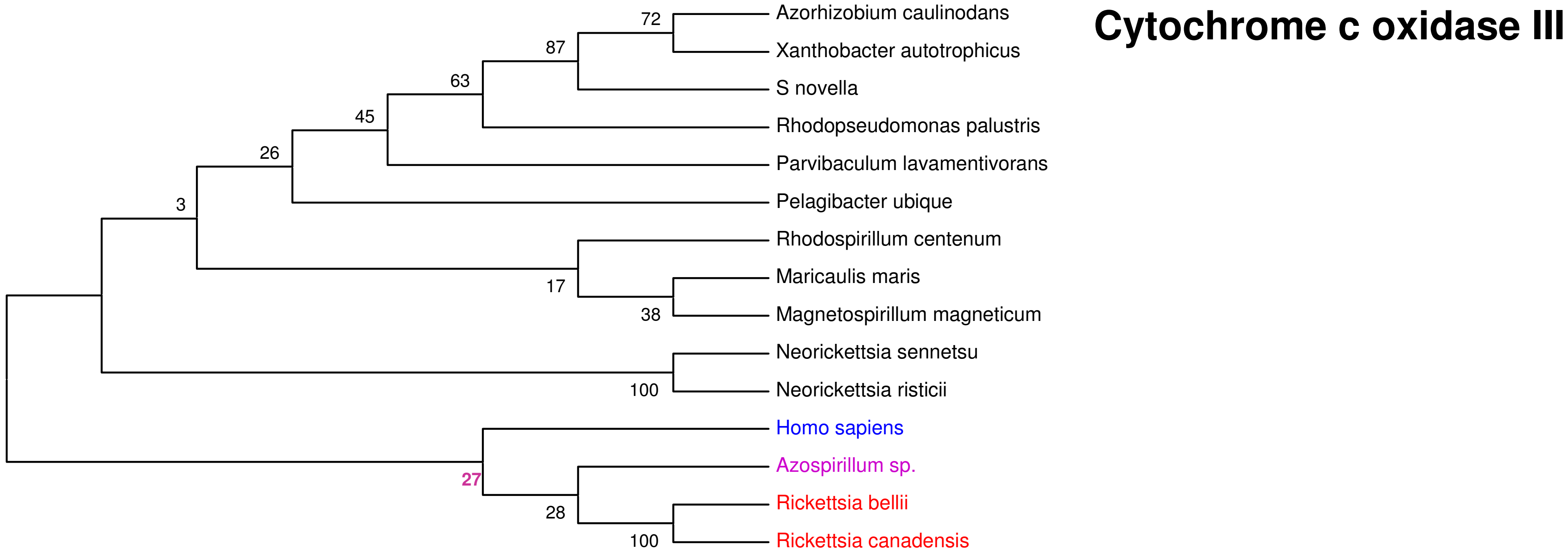
Cytochrome c oxidase III
72
87
63
45
26
3
17
38
100
27
28
100
Azorhizobium caulinodans
Xanthobacter autotrophicus
S novella
Rhodopseudomonas palustris
Parvibaculum lavamentivorans
Pelagibacter ubique
Rhodospirillum centenum
Maricaulis maris
Magnetospirillum magneticum
Neorickettsia sennetsu
Neorickettsia risticii
Homo sapiens
Azospirillum sp.
Rickettsia bellii
Rickettsia canadensis

NADH deshydrogenase su 2

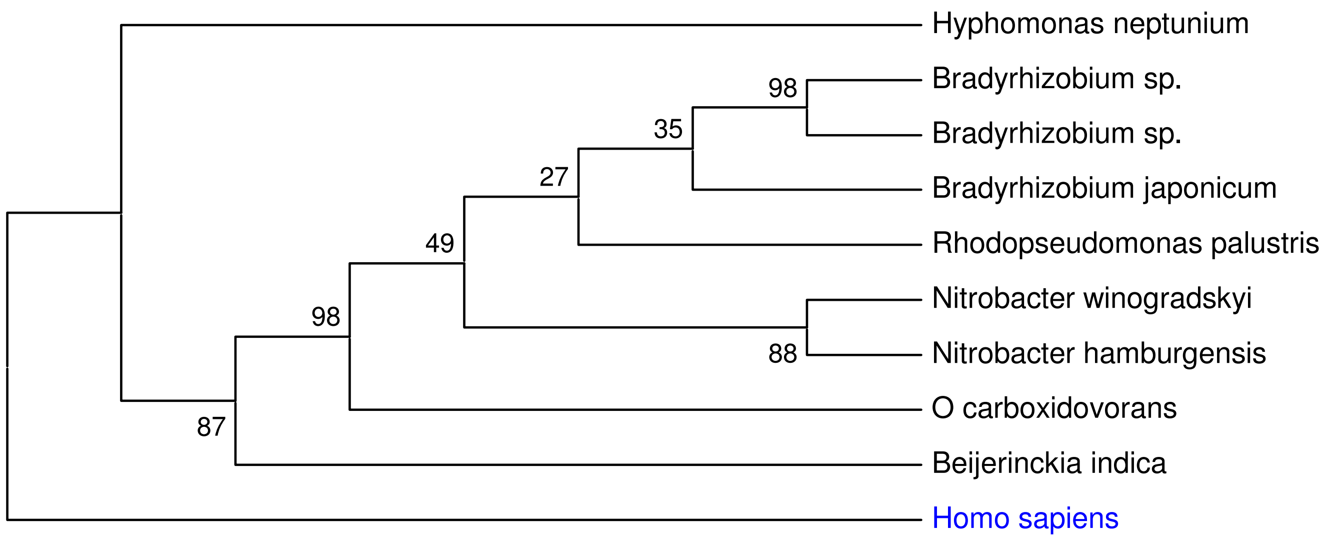

Supplement: Additional file 2 — Homo sapiens mitochondrial phylogenies. [file 1745-6150-6-55-S2.PDF]
