# Supplementary material for: The rhizome of Reclinomonas americana, Homo sapiens, Pediculus humanus and Saccharomyces cerevisiae mitochondria
Source: Biol Direct. 2011 Oct 20;6:55. doi: 10.1186/1745-6150-6-55 (PMC3214132; doi:10.1186/1745-6150-6-55)

Cytochrome beta

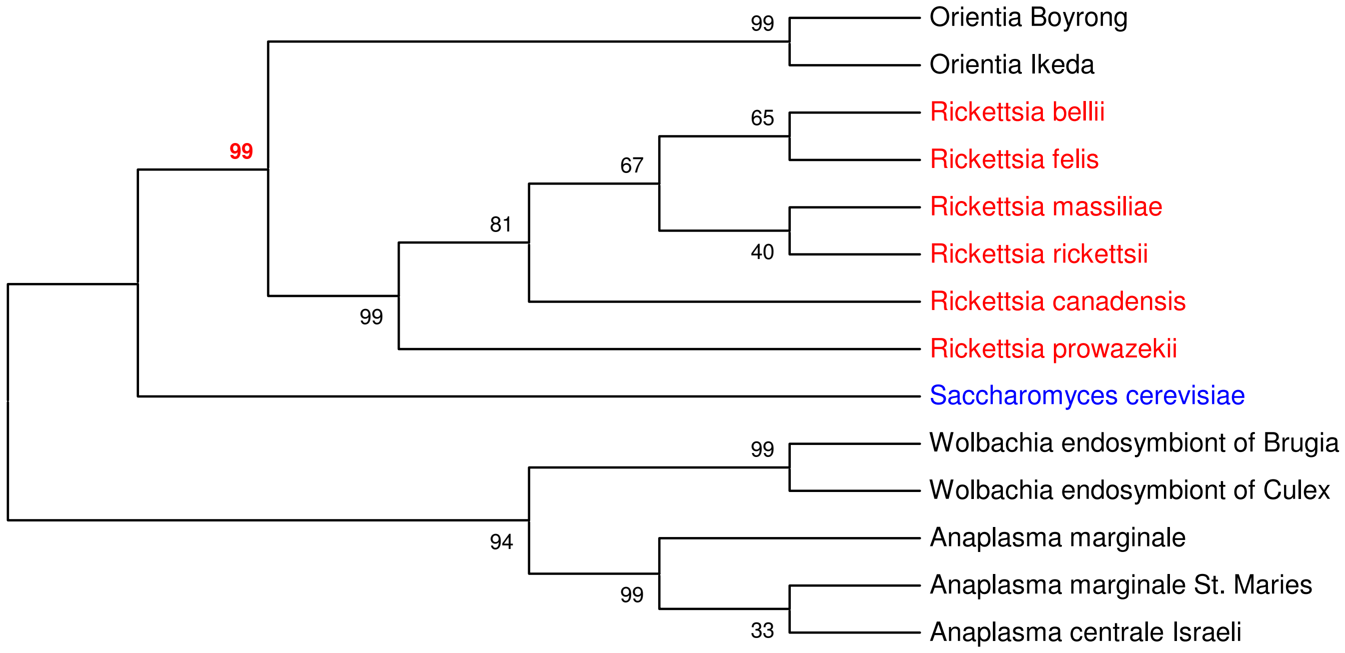

MutS

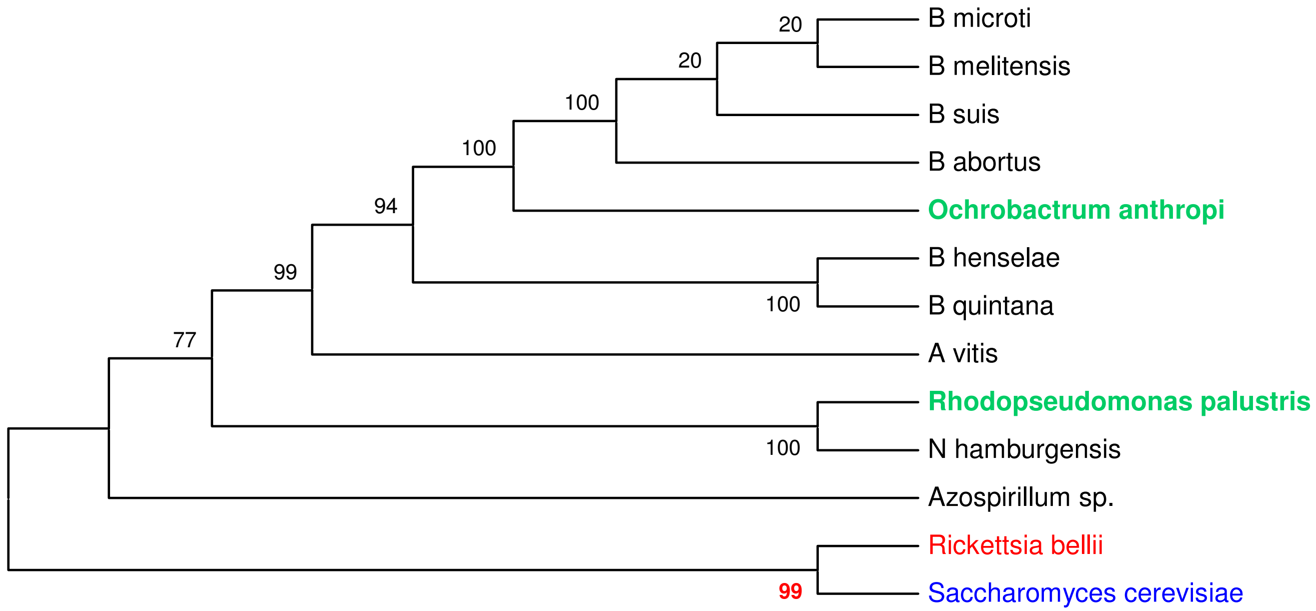

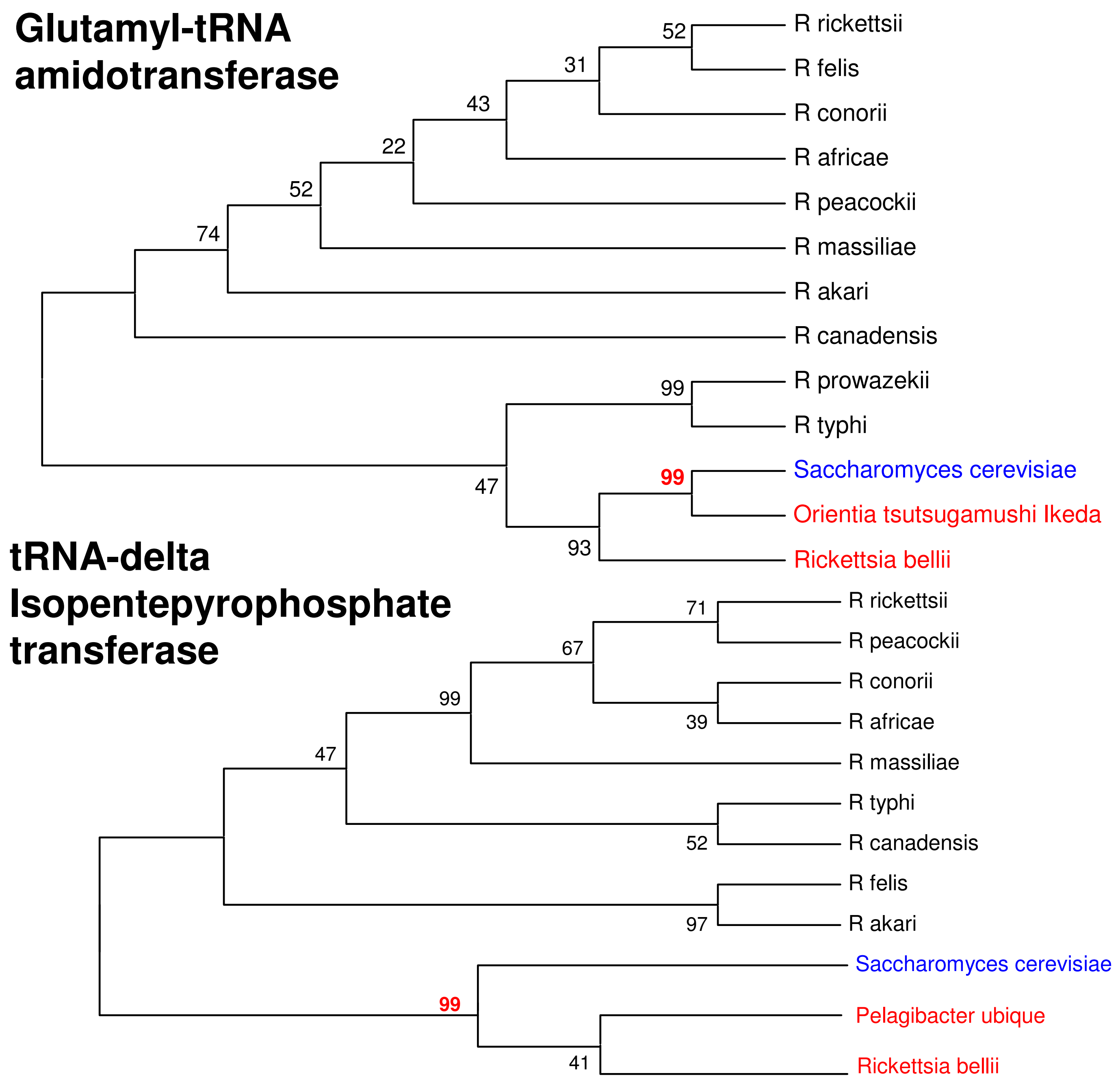
Glutamyl-tRNA amidotransferase
R rickettsii
R felis
R conorii
R africae
R peacockii
R massiliae
R akari
R canadensis
R prowazekii
R typhi
Saccharomyces cerevisiae
Orientia tsutsugamushi Ikeda
Rickettsia bellii
52
31
43
22
52
74
99
99
47
93
tRNA-delta Isopentepyrophosphate transferase
R rickettsii
R peacockii
R conorii
R africae
R massiliae
R typhi
R canadensis
R felis
R akari
Saccharomyces cerevisiae
Pelagibacter ubique
Rickettsia bellii
71
67
39
99
47
52
97
99
41

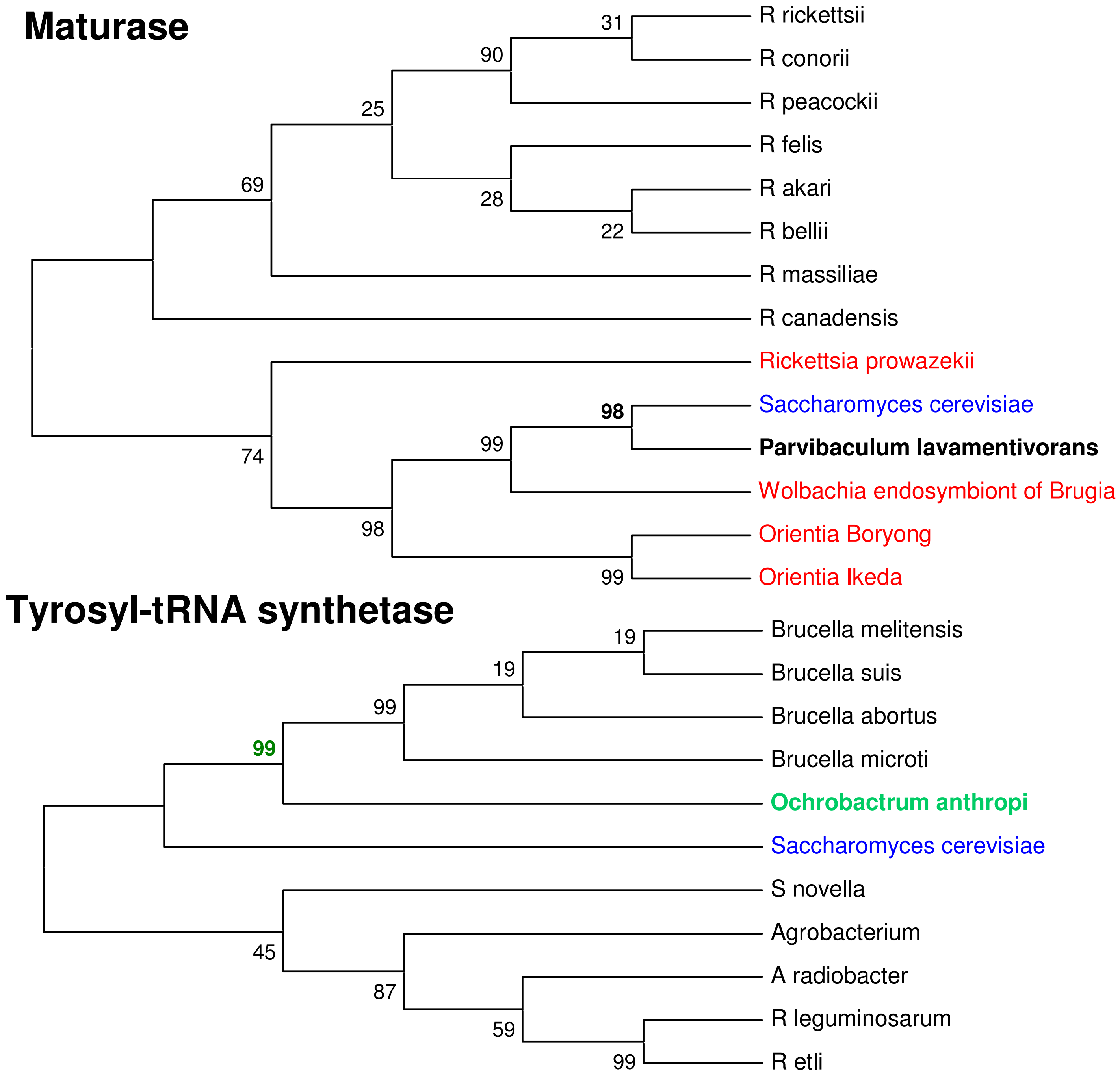
Maturase
R rickettsii
R conorii
R peacockii
R felis
R akari
R bellii
R massiliae
R canadensis
Rickettsia prowazekii
Saccharomyces cerevisiae
Parvibaculum lavamentivorans
Wolbachia endosymbiont of Brugia
Orientia Boryong
Orientia Ikeda
31
90
25
69
28
22
98
99
74
98
99
Tyrosyl-tRNA synthetase
Brucella melitensis
Brucella suis
Brucella abortus
Brucella microti
Ochrobactrum anthropi
Saccharomyces cerevisiae
S novella
Agrobacterium
A radiobacter
R leguminosarum
R etli
19
19
99
99
45
87
59
99

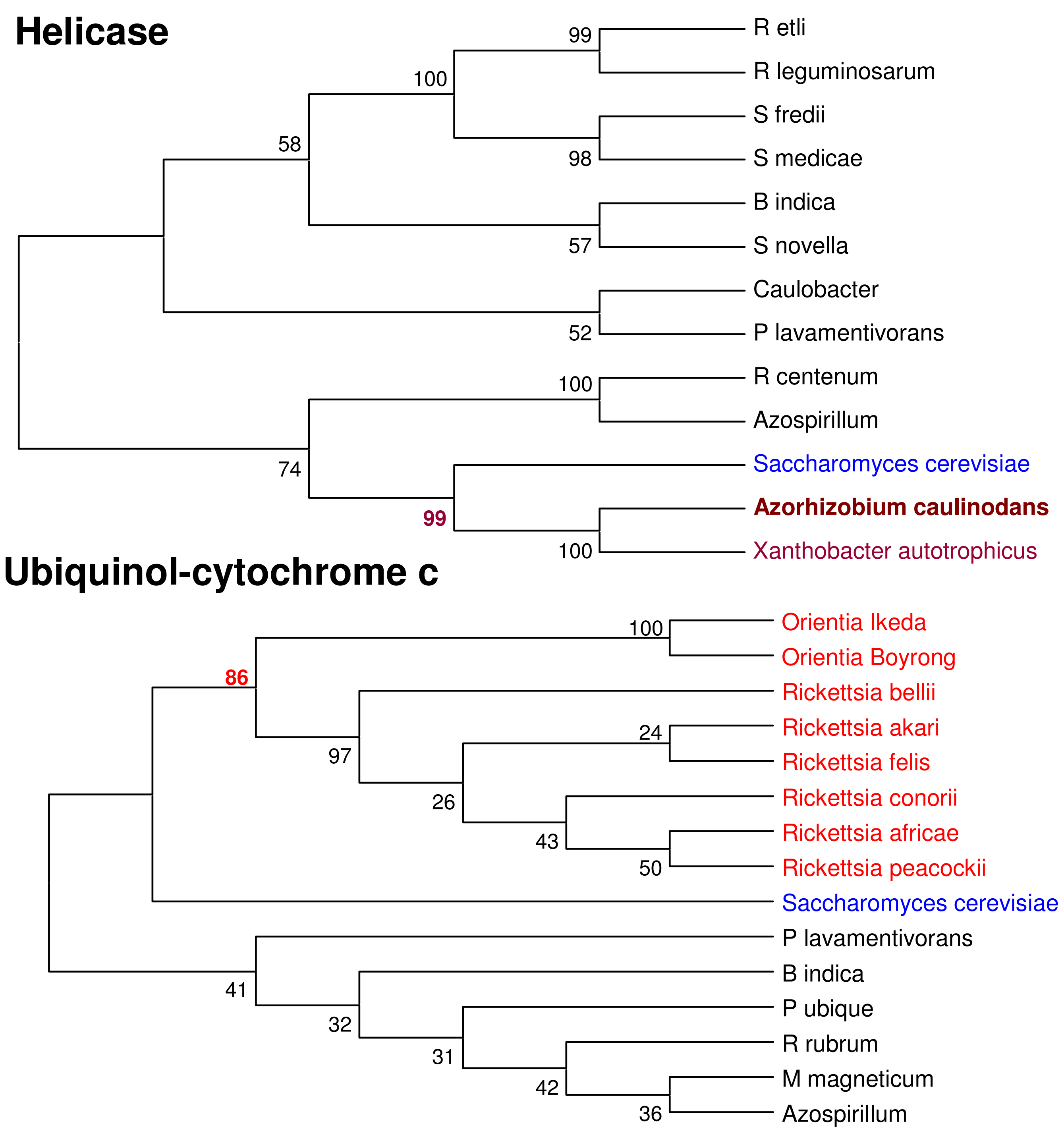
Helicase
R etli
R leguminosarum
S fredii
S medicae
B indica
S novella
Caulobacter
P lavamentivorans
R centenum
Azospirillum
Saccharomyces cerevisiae
Azorhizobium caulinodans
Xanthobacter autotrophicus
99
100
98
58
57
52
100
74
99
100
Ubiquinol-cytochrome c
Orientia Ikeda
Orientia Boyrong
Rickettsia bellii
Rickettsia akari
Rickettsia felis
Rickettsia conorii
Rickettsia africae
Rickettsia peacockii
Saccharomyces cerevisiae
P lavamentivorans
B indica
P ubique
R rubrum
M magneticum
Azospirillum
100
86
97
24
26
43
50
41
32
31
42
36

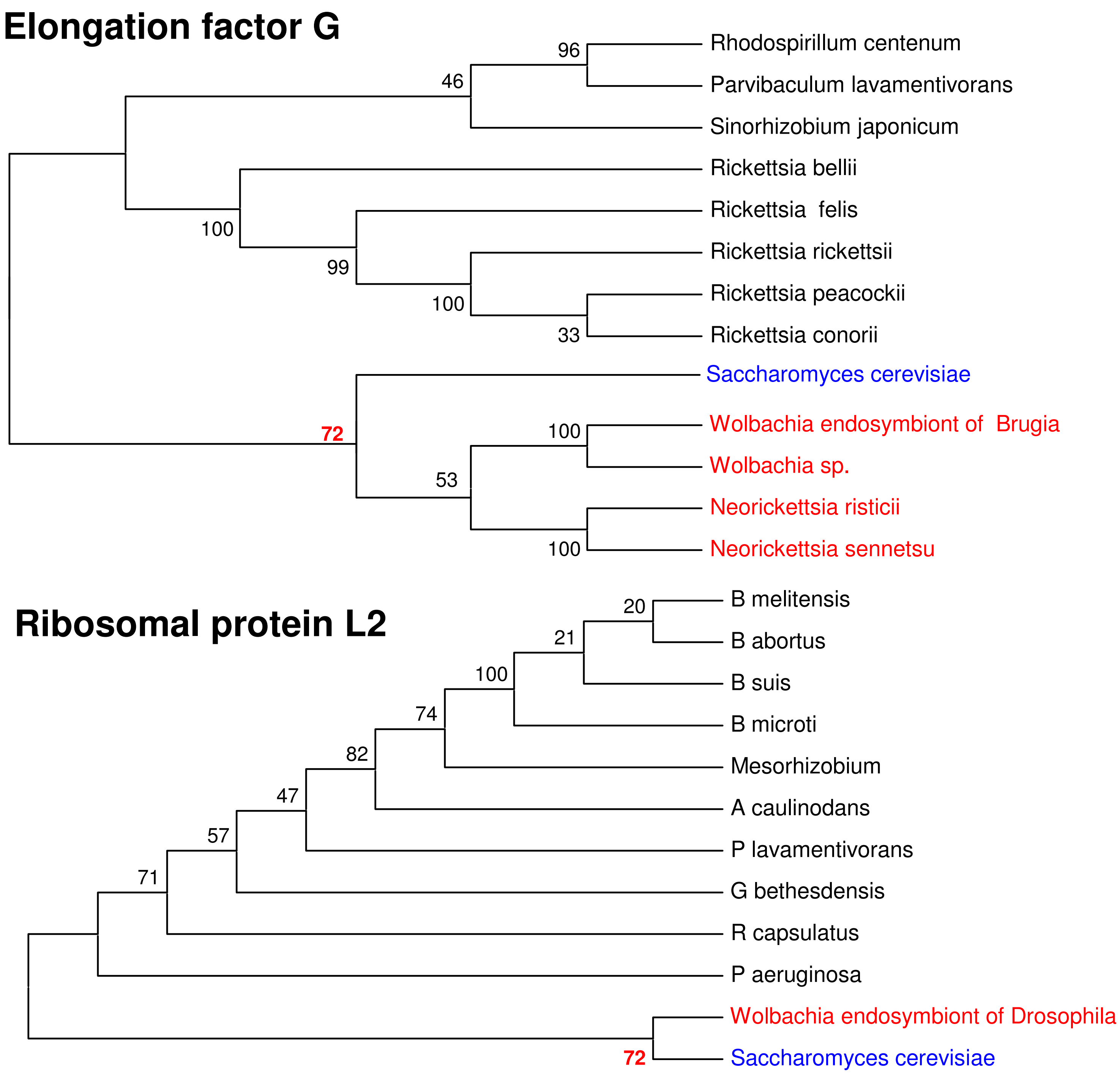

Elongation factor G
96
46
Rhodospirillum centenum
Parvibaculum lavamentivorans
Sinorhizobium japonicum
Rickettsia bellii
100
Rickettsia felis
99
Rickettsia rickettsii
100
Rickettsia peacockii
33
Rickettsia conorii
Saccharomyces cerevisiae
72
100
Wolbachia endosymbiont of Brugia
Wolbachia sp.
53
Neorickettsia risticii
100
Neorickettsia sennetsu
Ribosomal protein L2
20
B melitensis
21
B abortus
100
B suis
74
B microti
82
Mesorhizobium
47
A caulinodans
57
P lavamentivorans
71
G bethesdensis
R capsulatus
P aeruginosa
Wolbachia endosymbiont of Drosophila
72
Saccharomyces cerevisiae

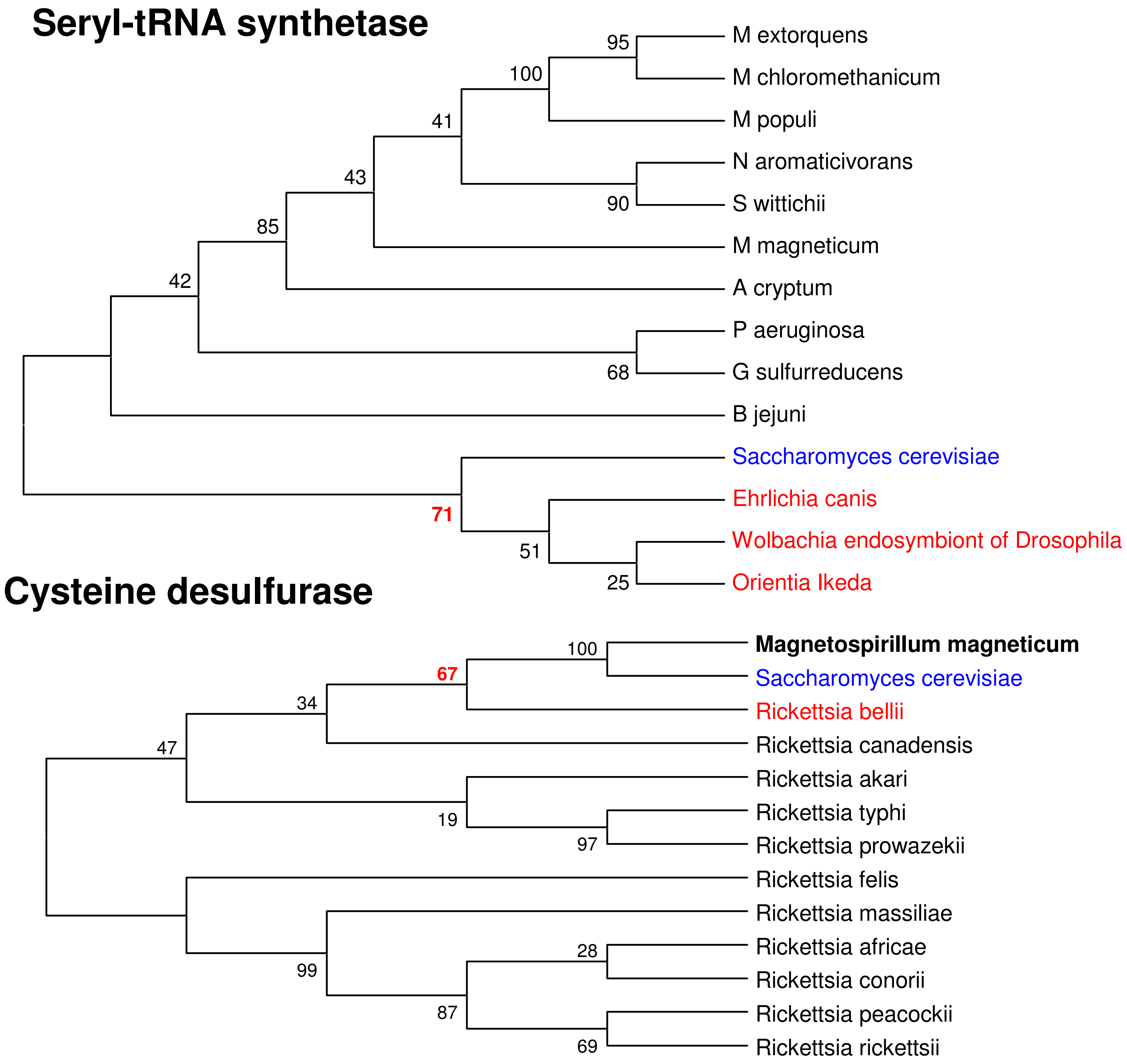
Seryl-tRNA synthetase
M extorquens
M chloromethanicum
M populi
N aromaticivorans
S wittichii
M magneticum
A cryptum
P aeruginosa
G sulfurreducens
B jejuni
Saccharomyces cerevisiae
Ehrlichia canis
Wolbachia endosymbiont of Drosophila
Orientia Ikeda
95
100
41
90
43
85
42
68
71
51
25
Cysteine desulfurase
Magnetospirillum magneticum
Saccharomyces cerevisiae
Rickettsia bellii
Rickettsia canadensis
Rickettsia akari
Rickettsia typhi
Rickettsia prowazekii
Rickettsia felis
Rickettsia massiliae
Rickettsia africae
Rickettsia conorii
Rickettsia peacockii
Rickettsia rickettsii
100
67
34
47
19
97
99
28
87
69

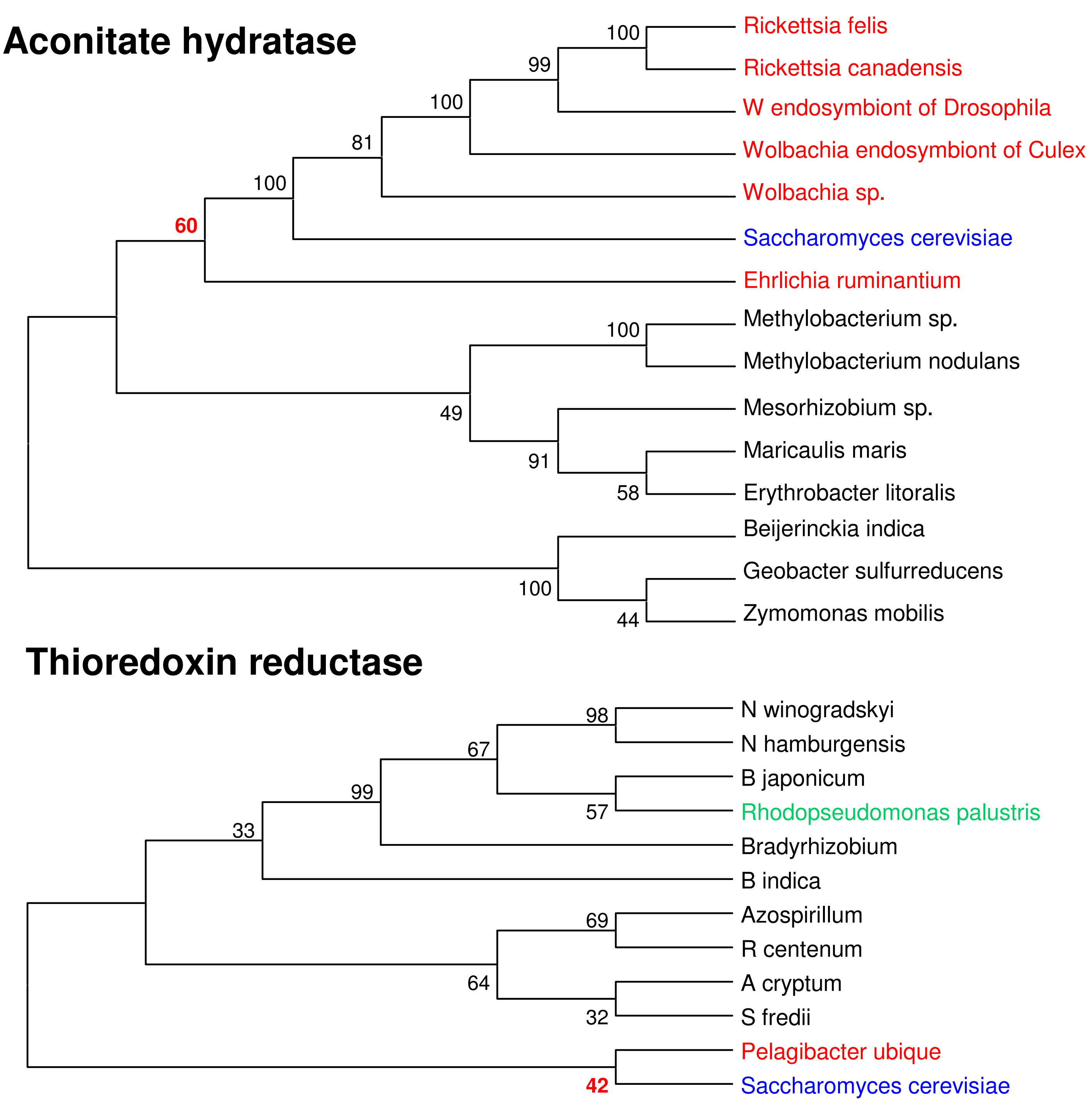
Aconitate hydratase
Rickettsia felis
Rickettsia canadensis
W endosymbiont of Drosophila
Wolbachia endosymbiont of Culex
Wolbachia sp.
Saccharomyces cerevisiae
Ehrlichia ruminantium
Methylobacterium sp.
Methylobacterium nodulans
Mesorhizobium sp.
Maricaulis maris
Erythrobacter litoralis
Beijerinckia indica
Geobacter sulfurreducens
Zymomonas mobilis
100
99
100
81
100
60
100
49
91
58
100
44
Thioredoxin reductase
N winogradskyi
N hamburgensis
B japonicum
Rhodopseudomonas palustris
Bradyrhizobium
B indica
Azospirillum
R centenum
A cryptum
S fredii
Pelagibacter ubique
Saccharomyces cerevisiae
98
67
57
99
33
69
64
32
42

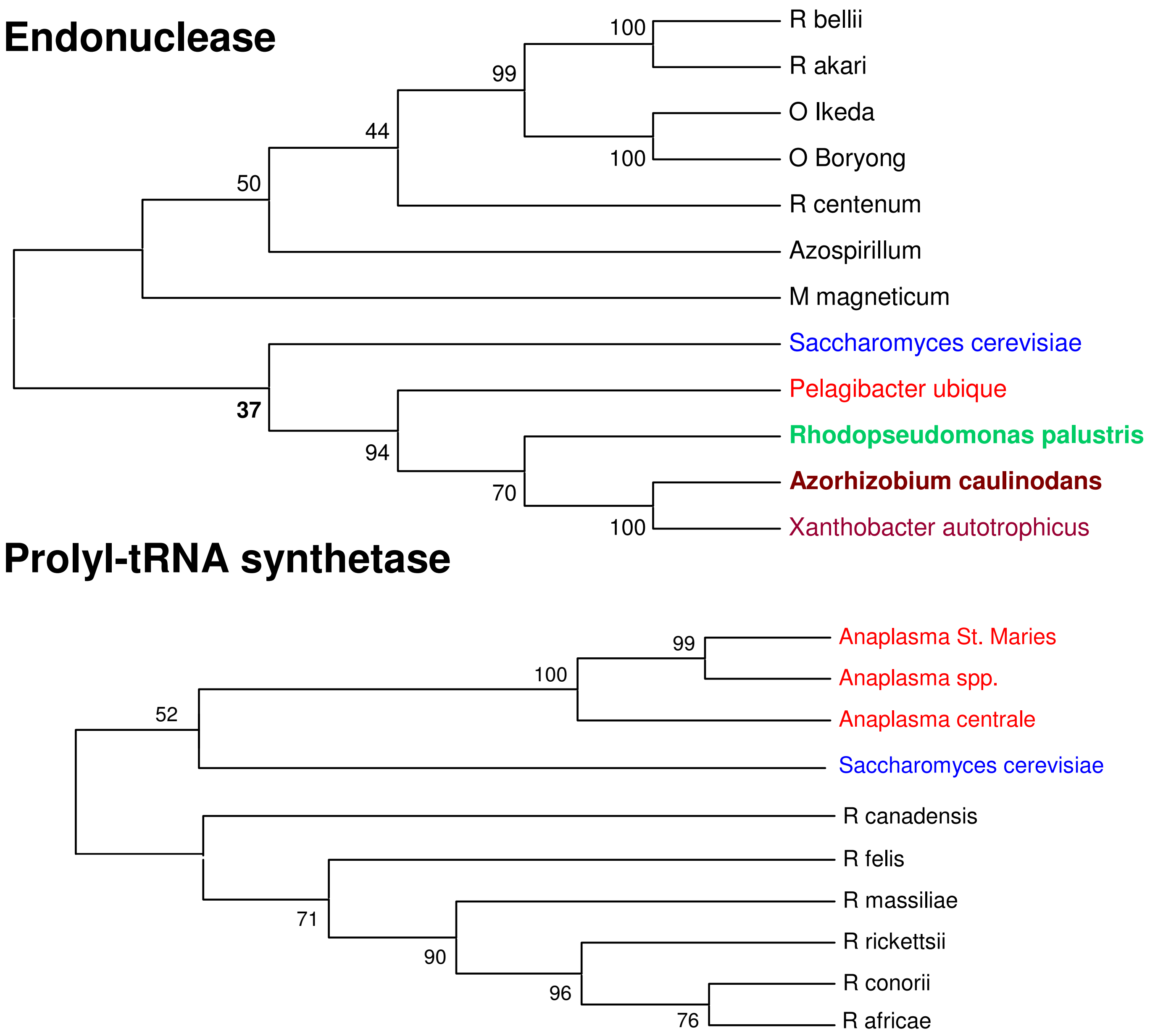
Endonuclease
R bellii
R akari
O Ikeda
O Boryong
R centenum
Azospirillum
M magneticum
Saccharomyces cerevisiae
Pelagibacter ubique
Rhodopseudomonas palustris
Azorhizobium caulinodans
Xanthobacter autotrophicus
100
99
44
100
50
37
94
70
100
Prolyl-tRNA synthetase
Anaplasma St. Maries
Anaplasma spp.
Anaplasma centrale
Saccharomyces cerevisiae
R canadensis
R felis
R massiliae
R rickettsii
R conorii
R africae
99
100
52
71
90
96
76

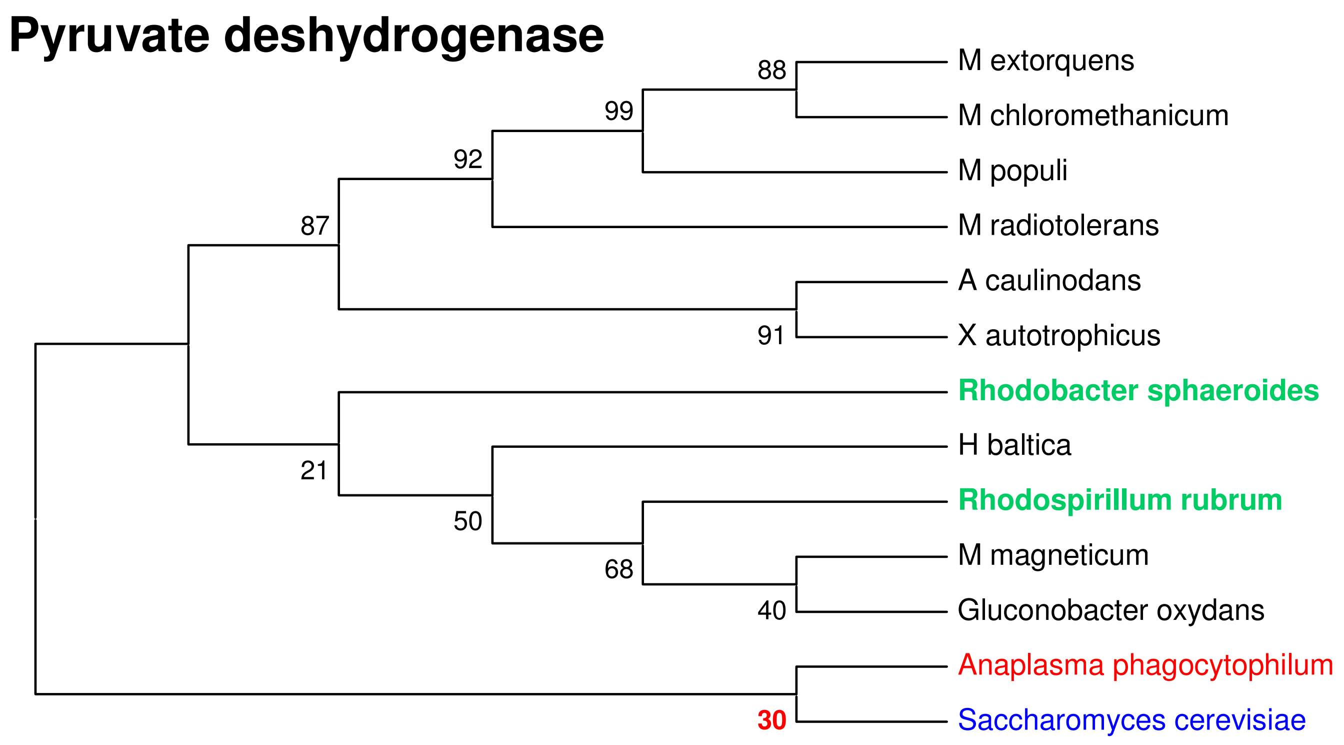
Pyruvate deshydrogenase
M extorquens
M chloromethanicum
M populi
M radiotolerans
A caulinodans
X autotrophicus
Rhodobacter sphaeroides
H baltica
Rhodospirillum rubrum
M magneticum
Gluconobacter oxydans
Anaplasma phagocytophilum
Saccharomyces cerevisiae
88
99
92
87
91
21
50
68
40
30

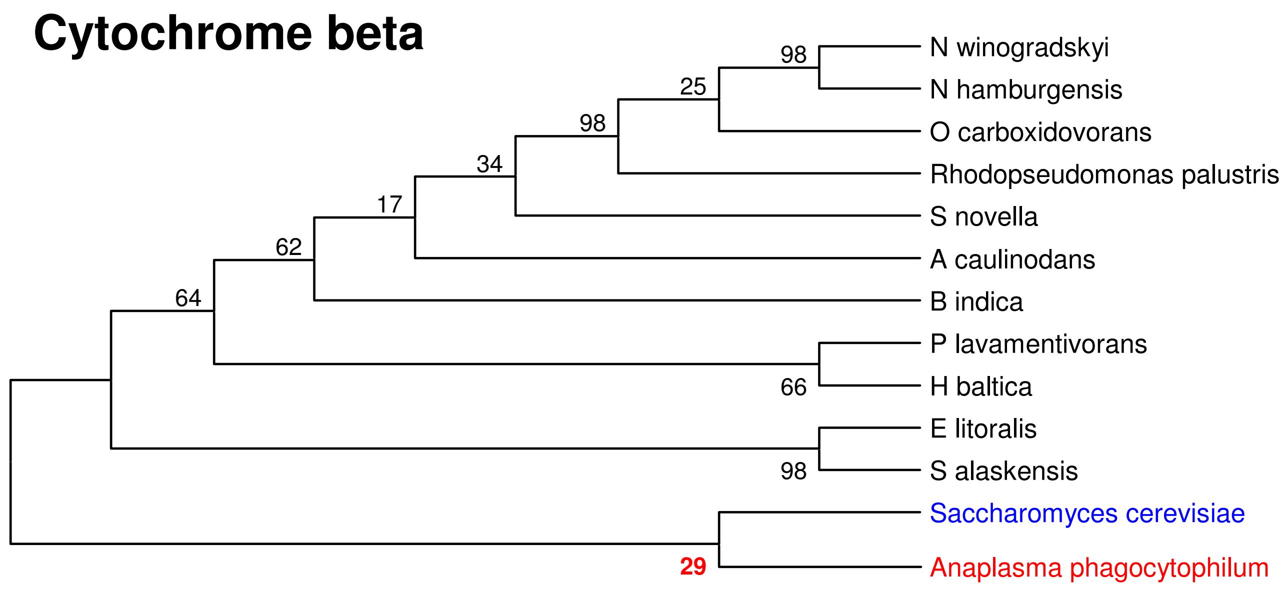
Cytochrome beta
N winogradskyi
N hamburgensis
O carboxidovorans
Rhodopseudomonas palustris
S novella
A caulinodans
B indica
P lavamentivorans
H baltica
E litoralis
S alaskensis
Saccharomyces cerevisiae
Anaplasma phagocytophilum
98
25
98
34
17
62
64
66
98
29

Ribosomal protein L3

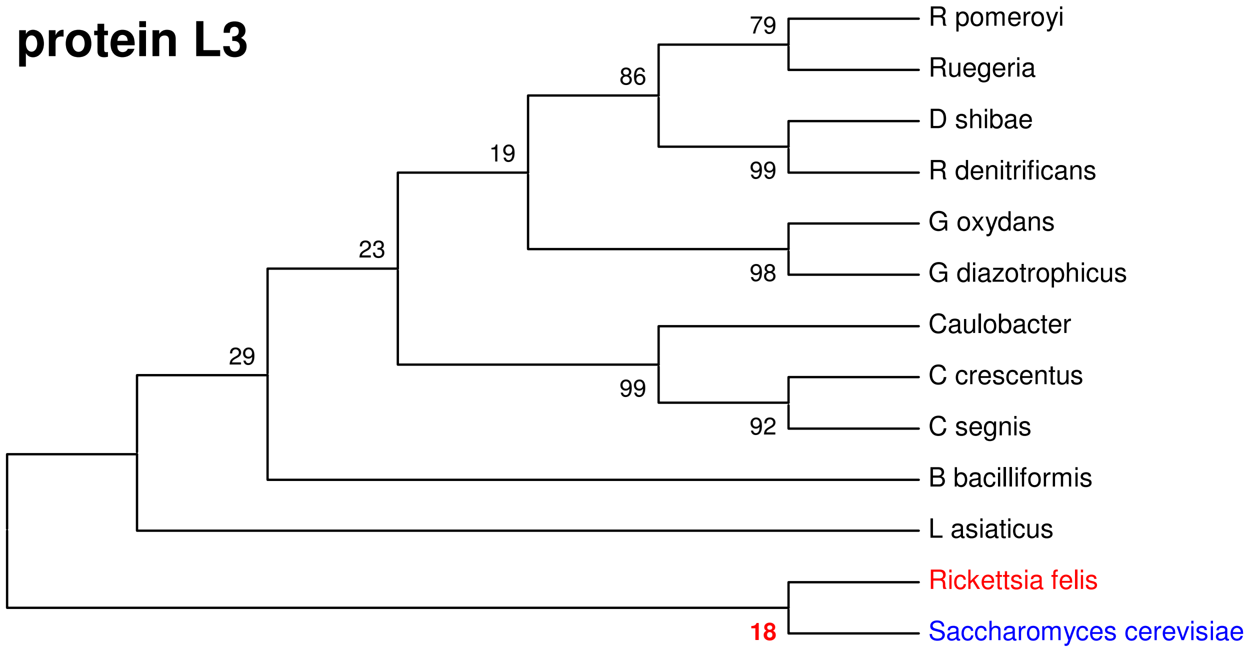

Arginyl-tRNA synthetase

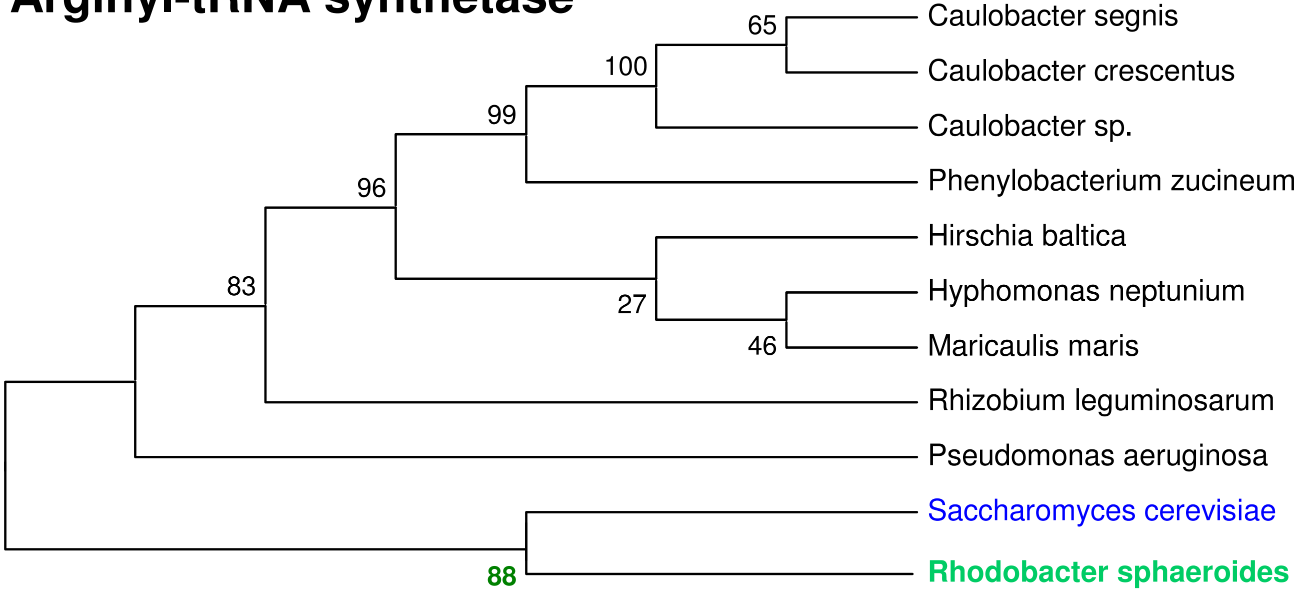

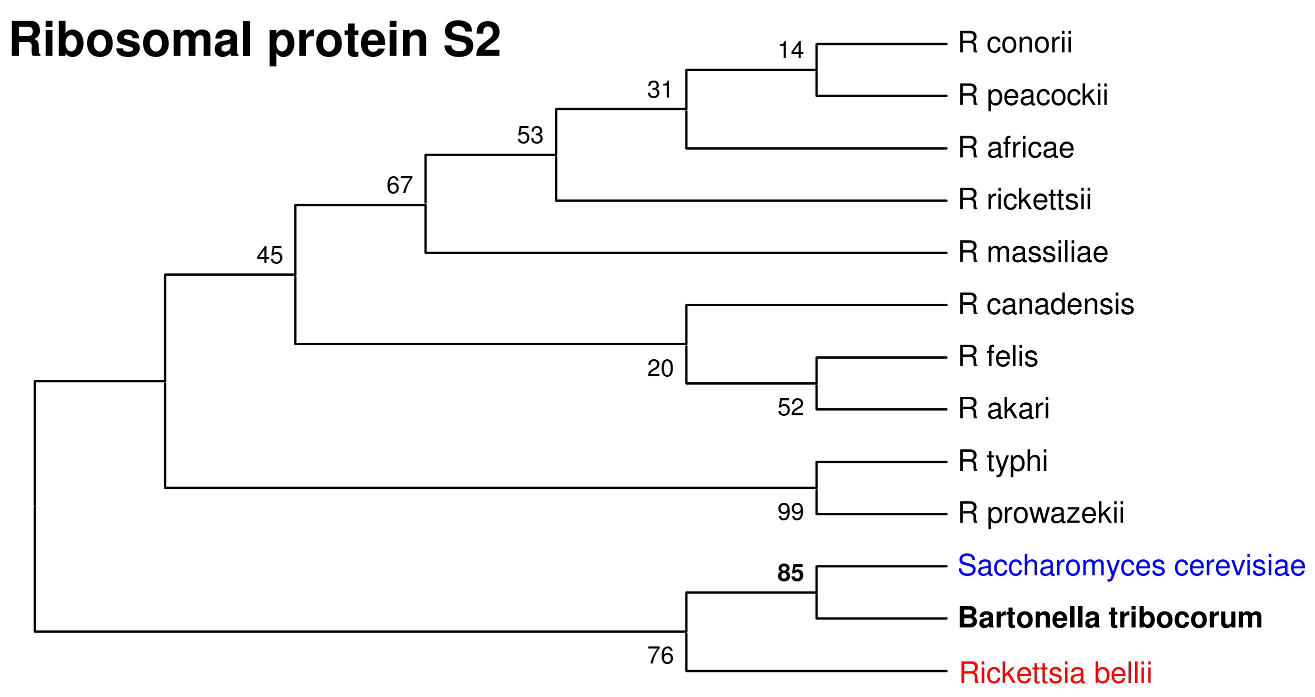
Ribosomal protein S2
14
31
53
67
45
20
52
99
85
76
R conorii
R peacockii
R africae
R rickettsii
R massiliae
R canadensis
R felis
R akari
R typhi
R prowazekii
Saccharomyces cerevisiae
Bartonella tribocorum
Rickettsia bellii

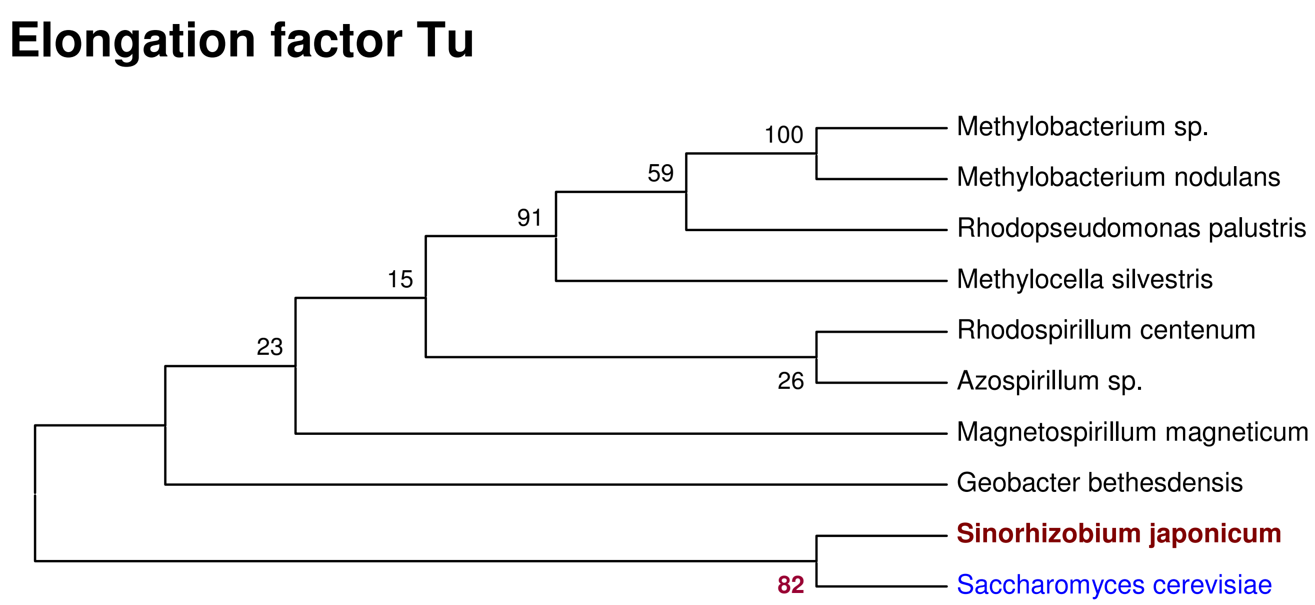
Elongation factor Tu
100
59
91
15
23
26
82
Methylobacterium sp.
Methylobacterium nodulans
Rhodopseudomonas palustris
Methylocella silvestris
Rhodospirillum centenum
Azospirillum sp.
Magnetospirillum magneticum
Geobacter bethesdensis
Sinorhizobium japonicum
Saccharomyces cerevisiae

Ribosomal protein S12

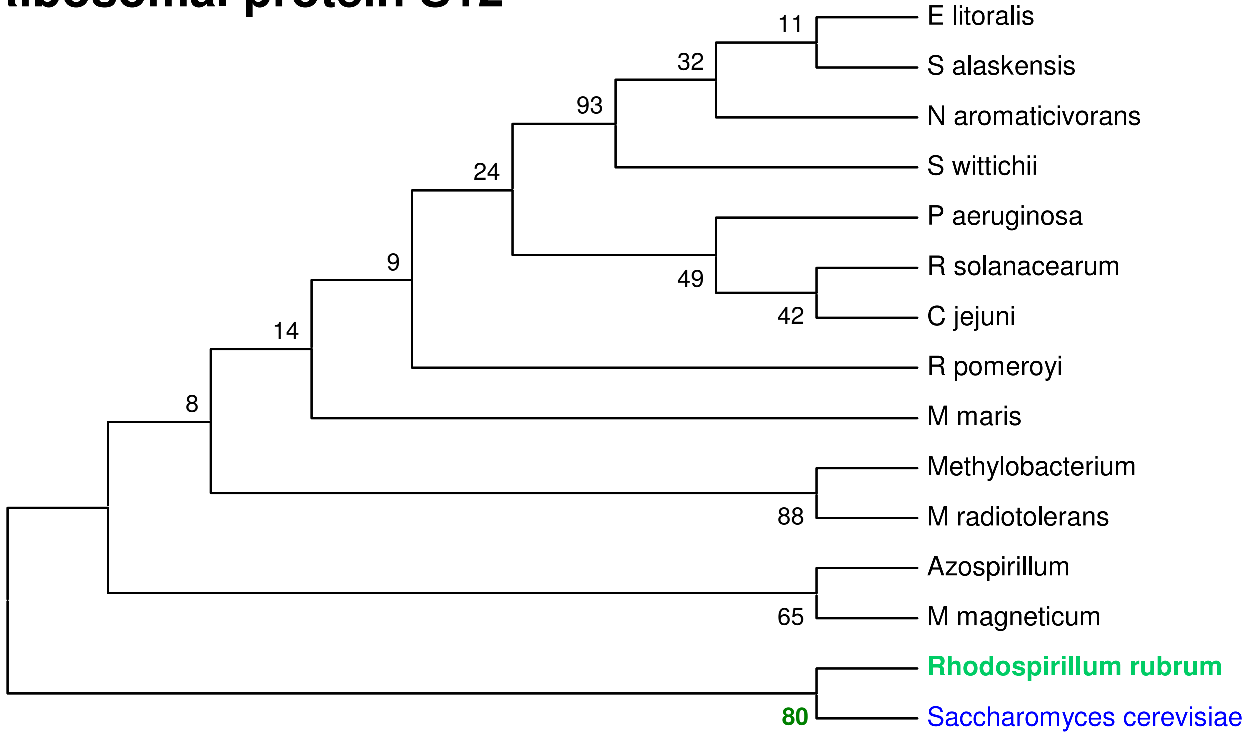

DnaK

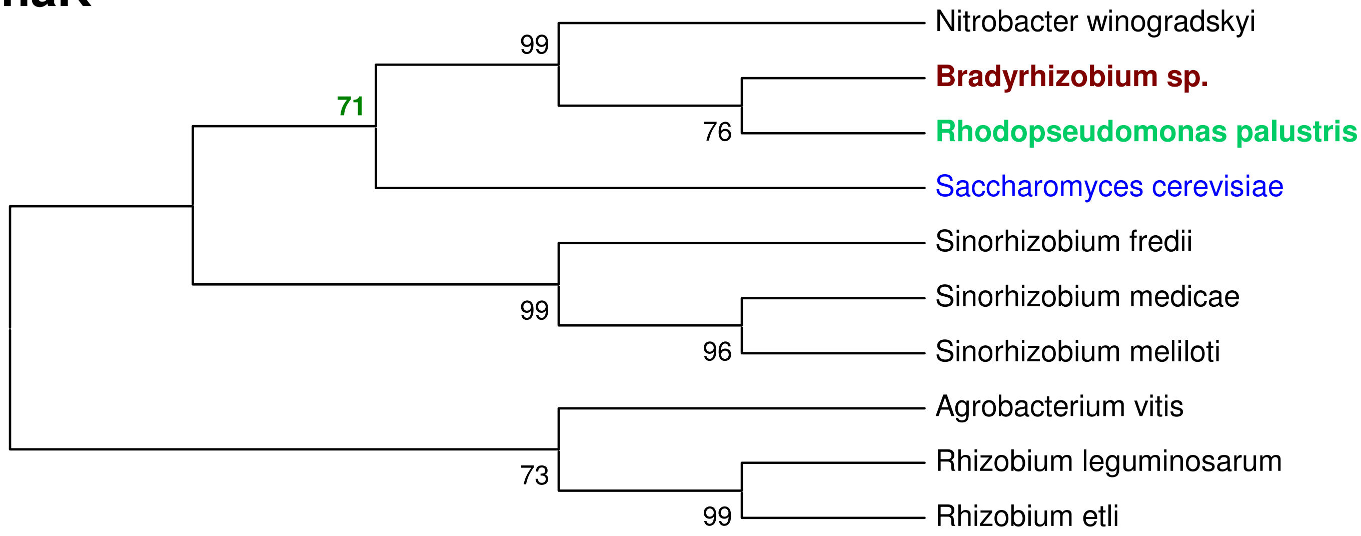

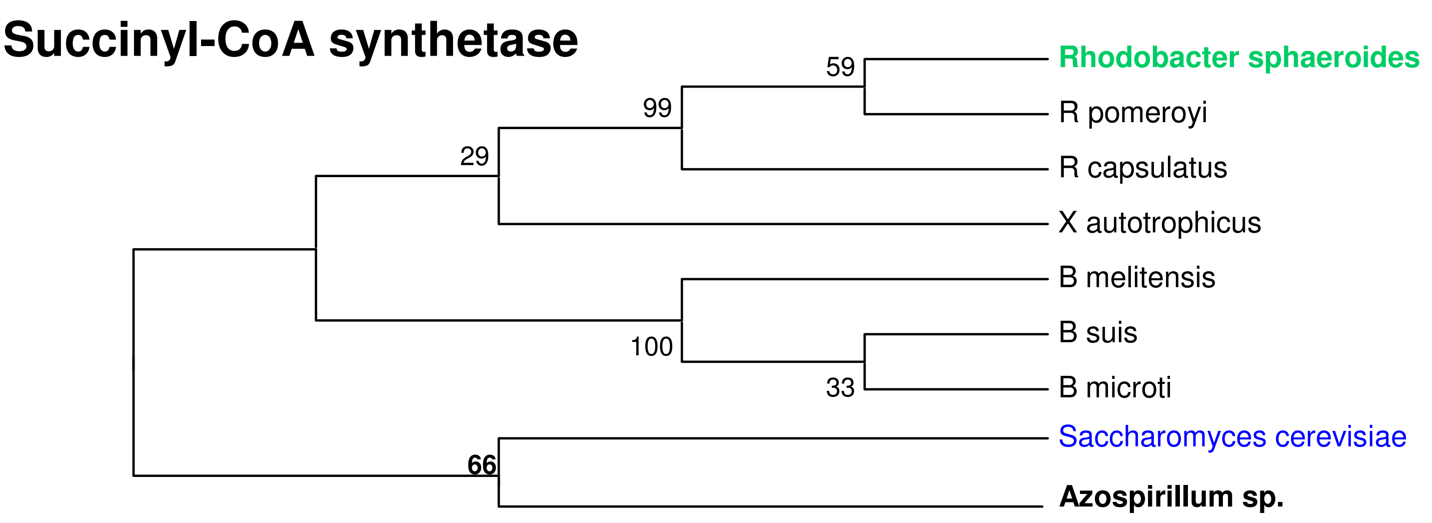

Succinyl-CoA synthetase
59
99
29
100
33
66
Rhodobacter sphaeroides
R pomeroyi
R capsulatus
X autotrophicus
B melitensis
B suis
B microti
Saccharomyces cerevisiae
Azospirillum sp.

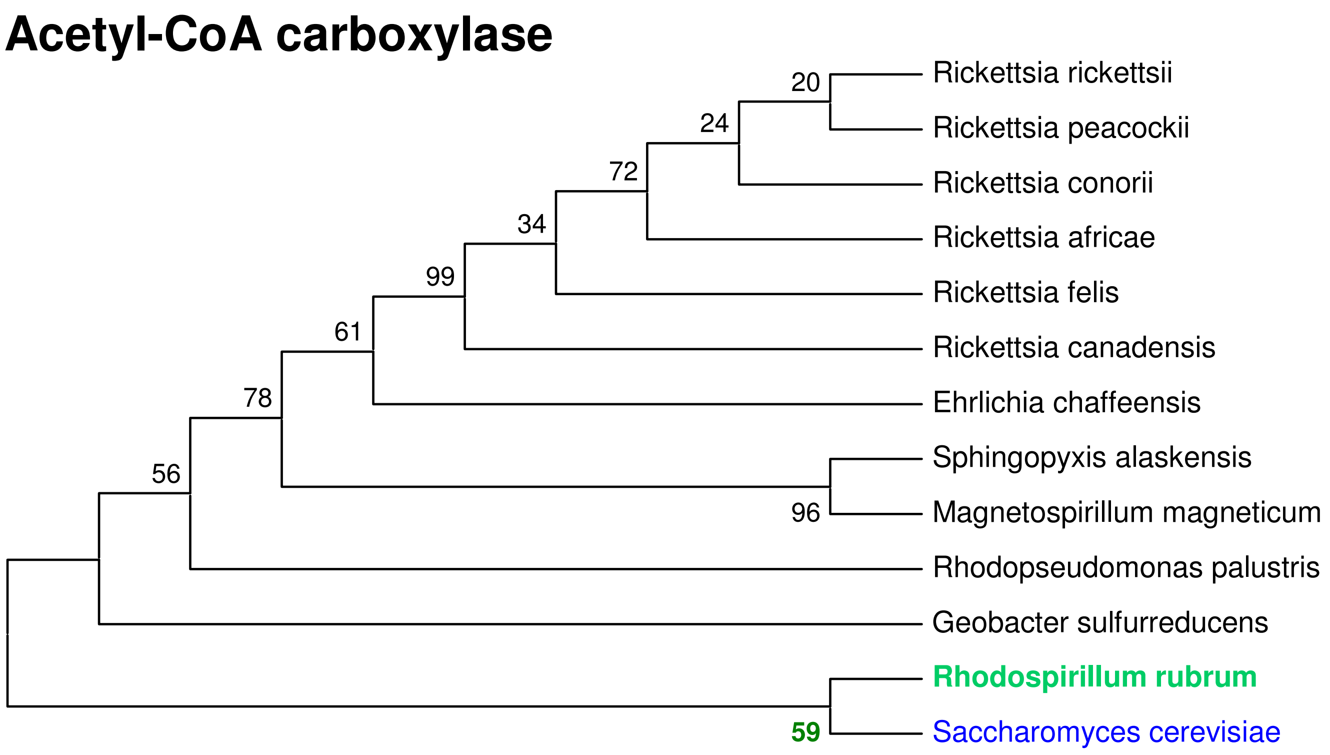

Acetyl-CoA carboxylase
20
24
72
34
99
61
78
56
96
59
Rickettsia rickettsii
Rickettsia peacockii
Rickettsia conorii
Rickettsia africae
Rickettsia felis
Rickettsia canadensis
Ehrlichia chaffeensis
Sphingopyxis alaskensis
Magnetospirillum magneticum
Rhodopseudomonas palustris
Geobacter sulfurreducens
Rhodospirillum rubrum
Saccharomyces cerevisiae

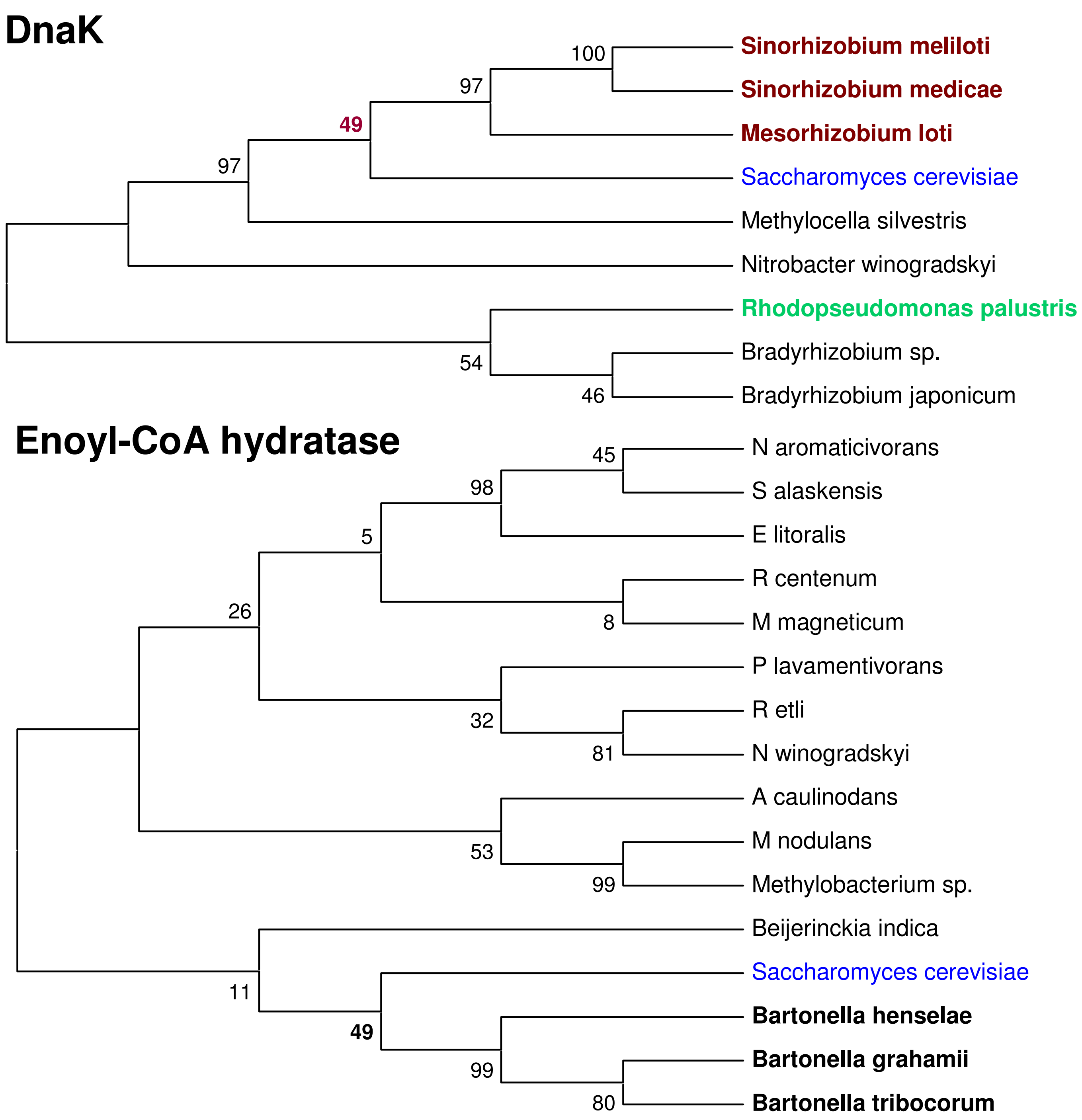
DnaK
100
97
49
97
54
46
Sinorhizobium meliloti
Sinorhizobium medicae
Mesorhizobium loti
Saccharomyces cerevisiae
Methylocella silvestris
Nitrobacter winogradskyi
Rhodopseudomonas palustris
Bradyrhizobium sp.
Bradyrhizobium japonicum
Enoyl-CoA hydratase
45
98
5
26
8
32
81
53
99
11
49
99
80
N aromaticivorans
S alaskensis
E litoralis
R centenum
M magneticum
P lavamentivorans
R etli
N winogradskyi
A caulinodans
M nodulans
Methylobacterium sp.
Beijerinckia indica
Saccharomyces cerevisiae
Bartonella henselae
Bartonella grahamii
Bartonella tribocorum

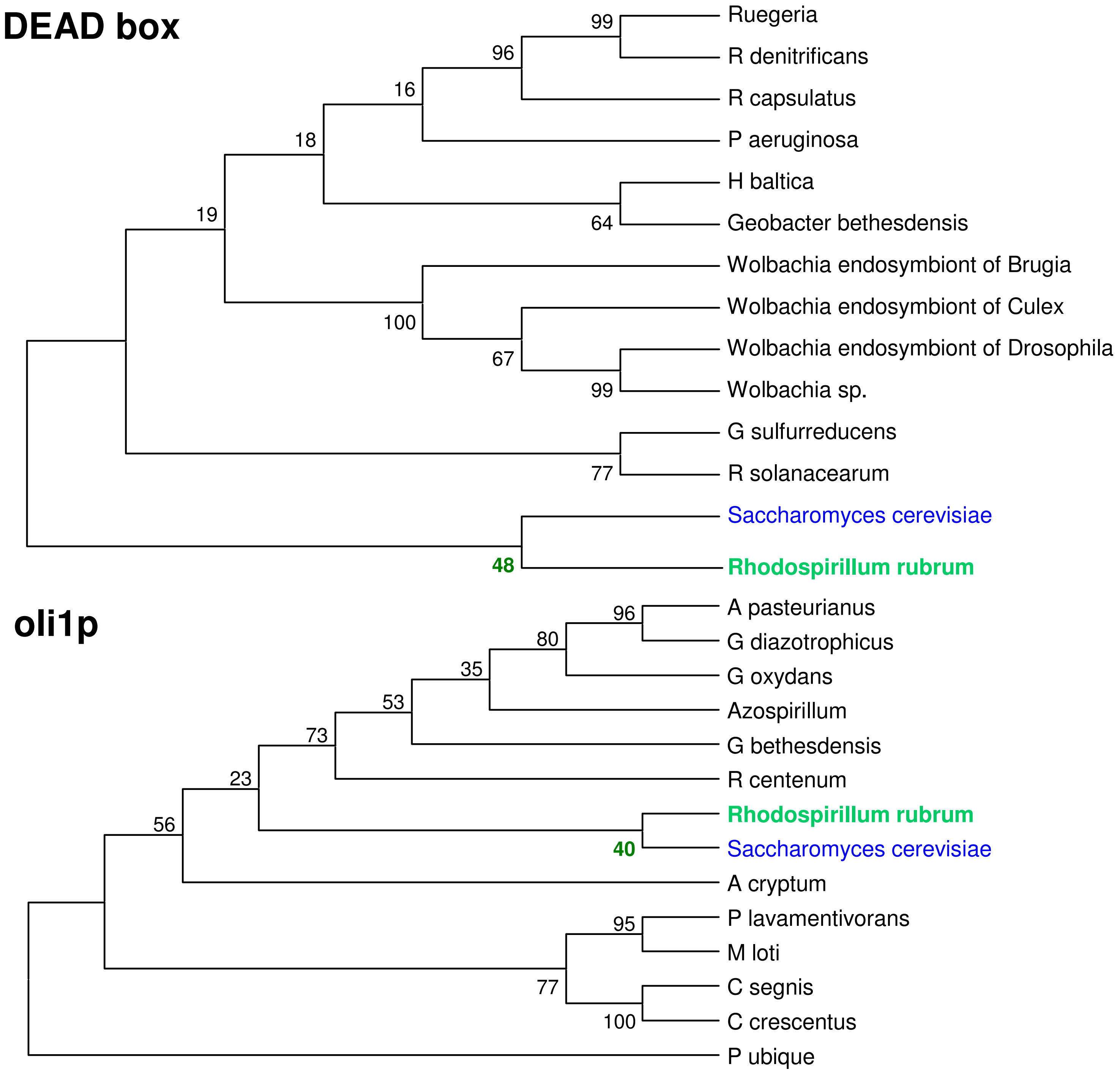
DEAD box
Ruegeria
R denitrificans
R capsulatus
P aeruginosa
H baltica
Geobacter bethesdensis
Wolbachia endosymbiont of Brugia
Wolbachia endosymbiont of Culex
Wolbachia endosymbiont of Drosophila
Wolbachia sp.
G sulfurreducens
R solanacearum
Saccharomyces cerevisiae
Rhodospirillum rubrum
99
96
16
18
19
64
100
67
99
77
48
oli1p
A pasteurianus
G diazotrophicus
G oxydans
Azospirillum
G bethesdensis
R centenum
Rhodospirillum rubrum
Saccharomyces cerevisiae
A cryptum
P lavamentivorans
M loti
C segnis
C crescentus
P ubique
96
80
35
53
73
23
56
40
95
77
100

Glutathione oxidoreductase

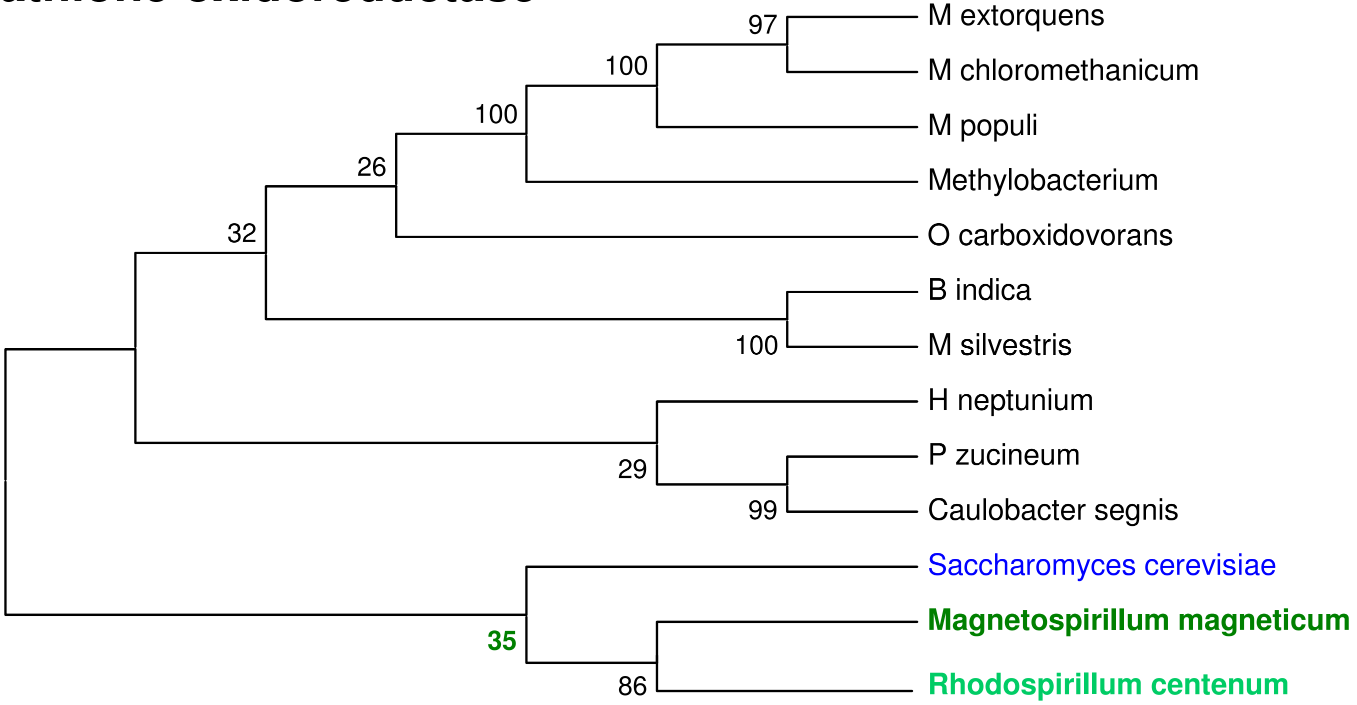

GroEL

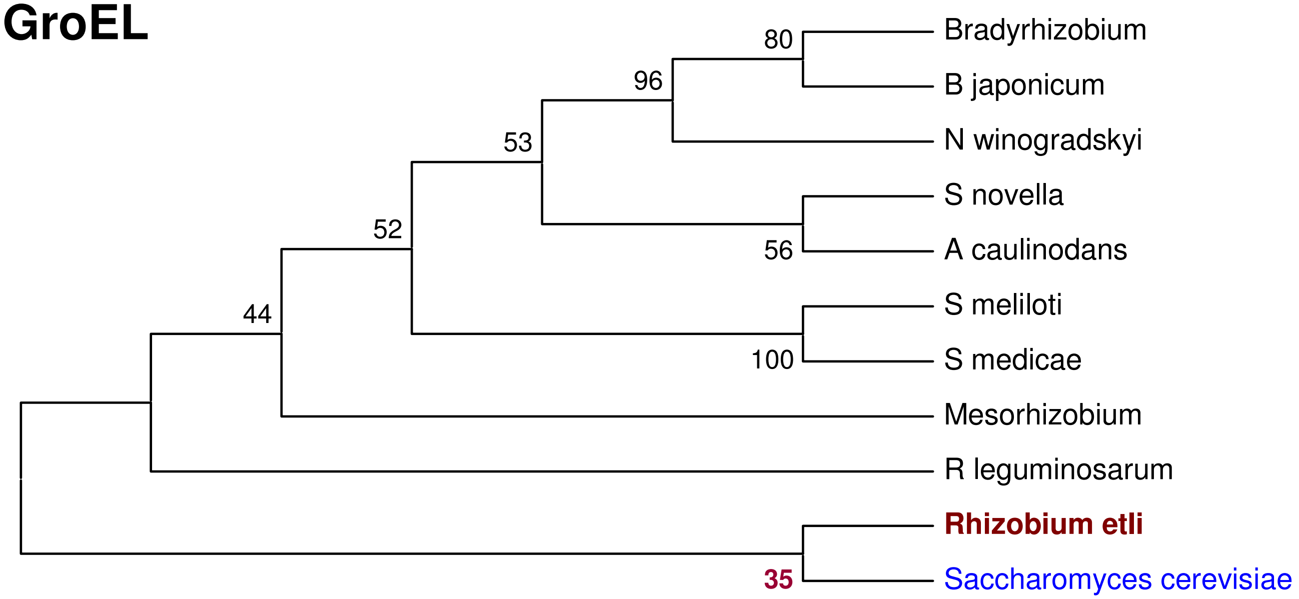

Formate tetrahydrofolate ligase

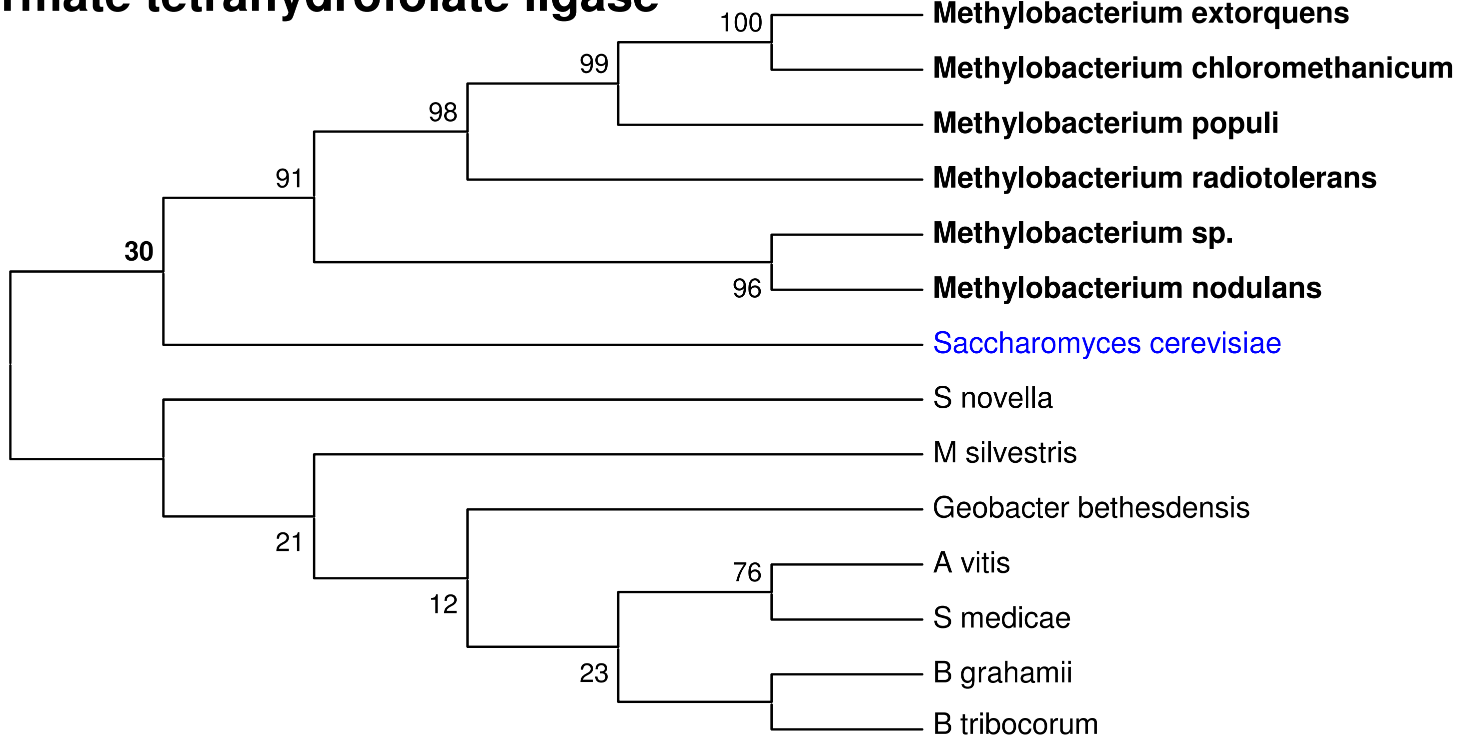

Dihydrolipoamide

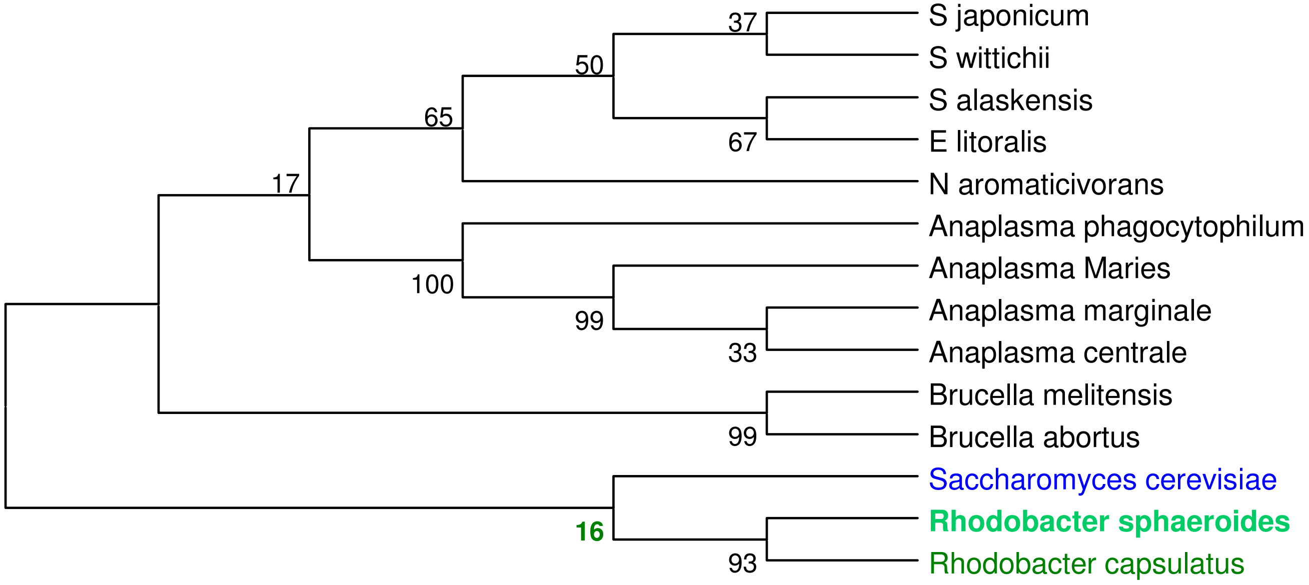

Fumarate hydratase

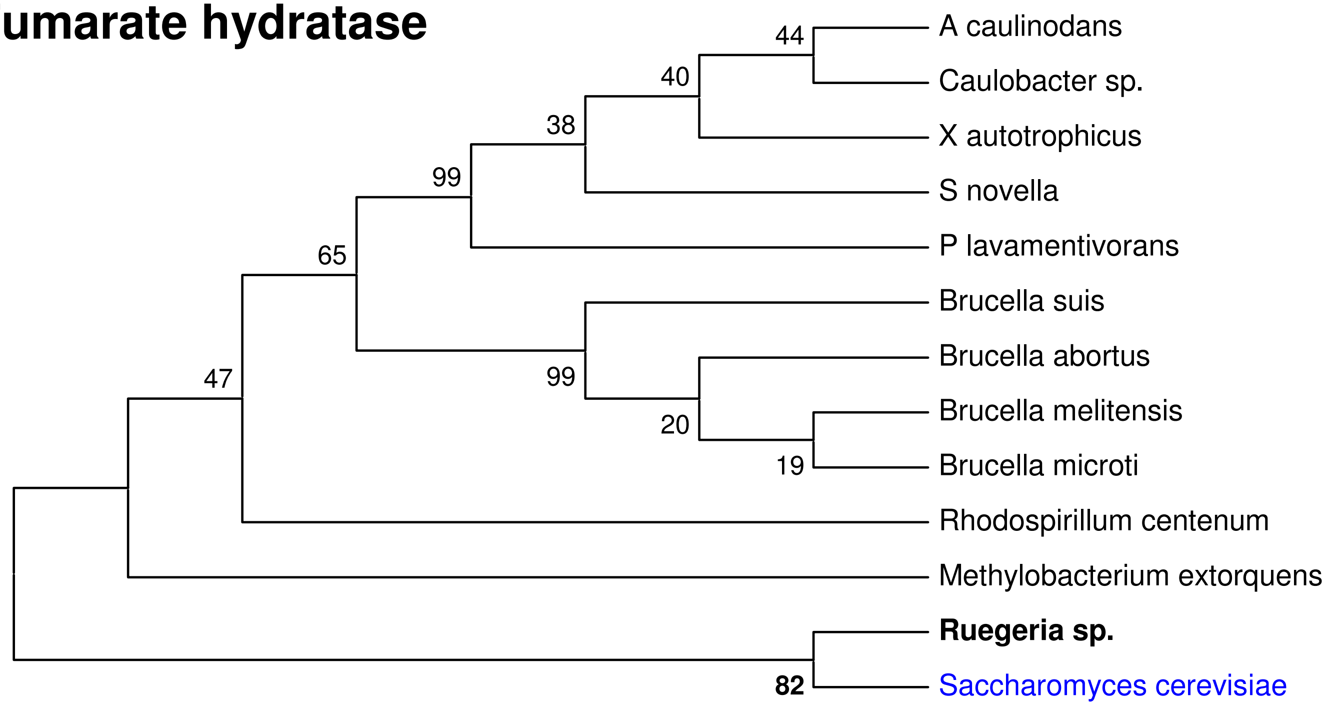

Deshydrogenase

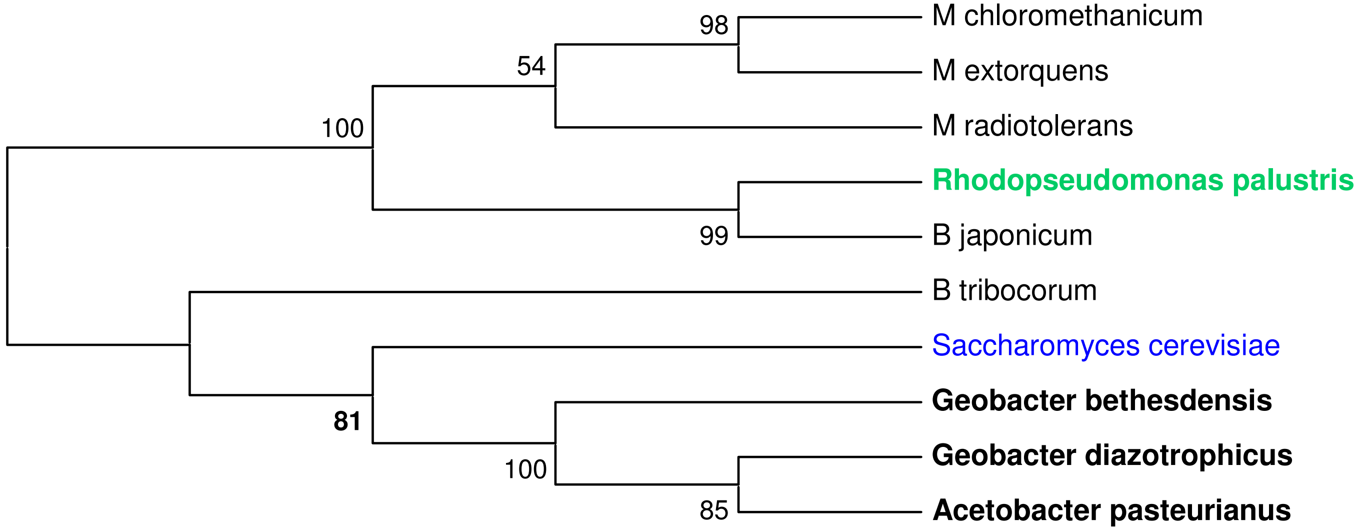

Reverse transcriptase

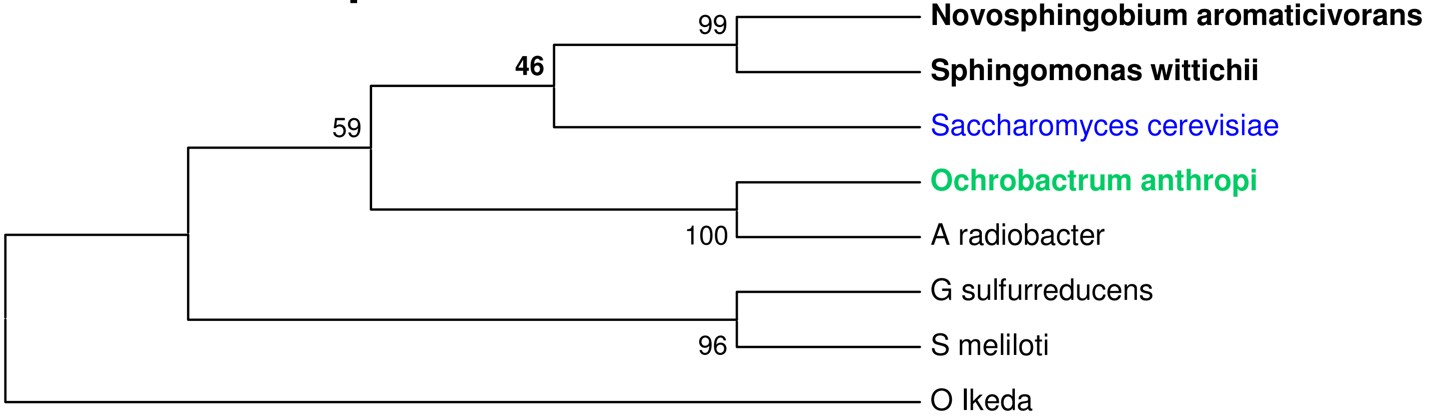

Tryptophanyl-tRNA synthetase

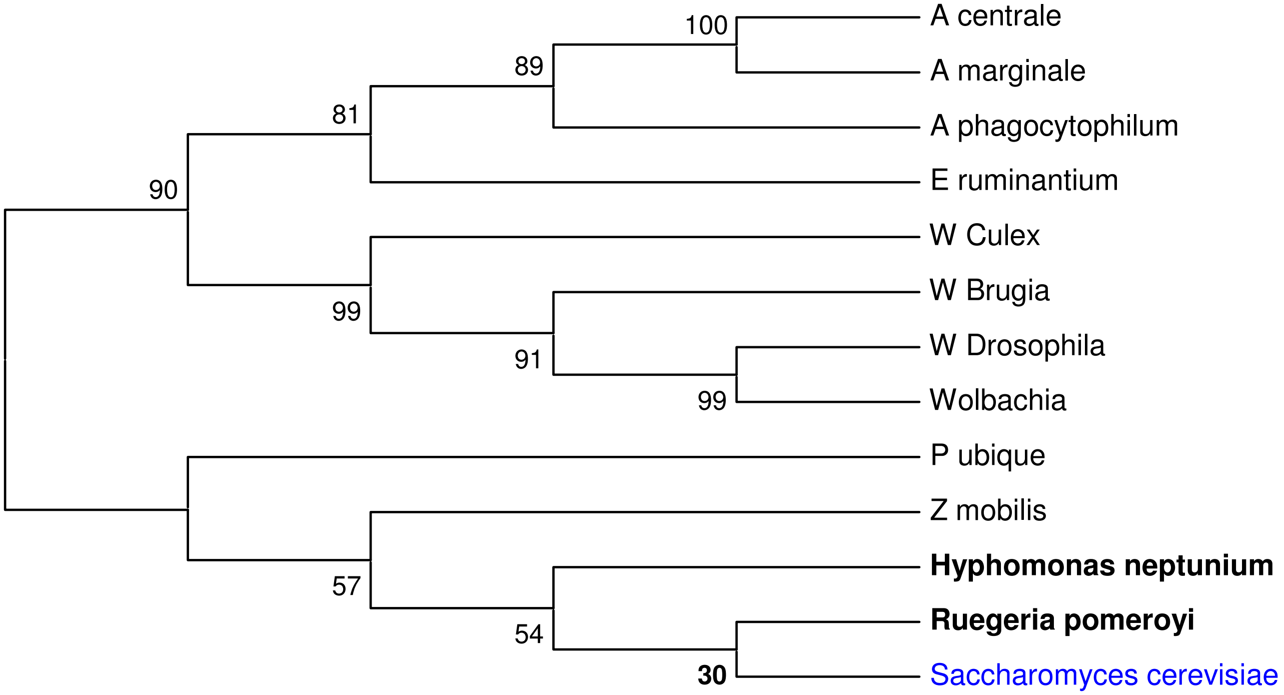

5- aminolevulinate

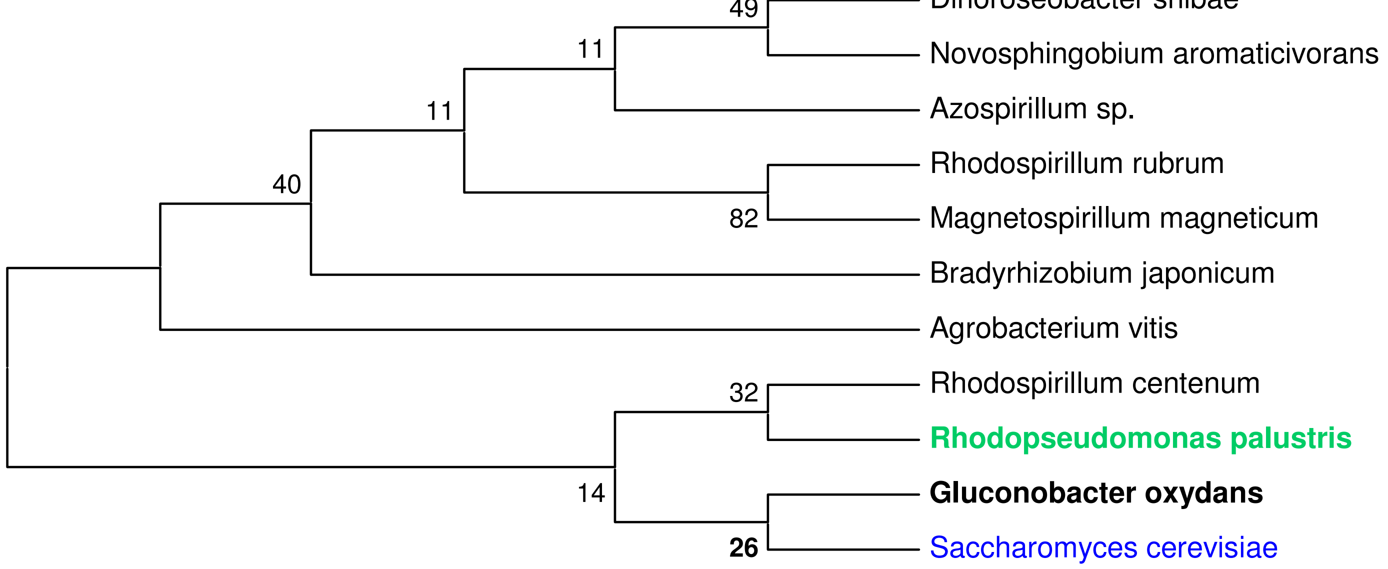

Sco1

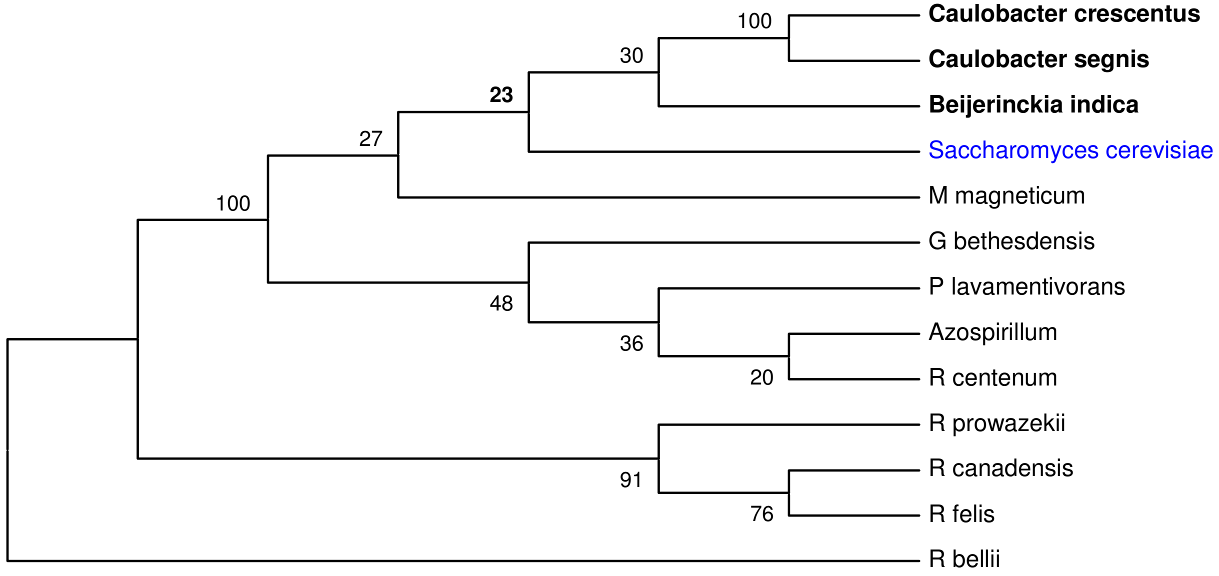

Supplement: Additional file 3 — Saccharomyces cerevisiae mitochondrial phylogenies. [file 1745-6150-6-55-S3.PDF]
