# Supplementary material for: The rhizome of Reclinomonas americana, Homo sapiens, Pediculus humanus and Saccharomyces cerevisiae mitochondria
Source: Biol Direct. 2011 Oct 20;6:55. doi: 10.1186/1745-6150-6-55 (PMC3214132; doi:10.1186/1745-6150-6-55)
Supplement: Additional file 4 — Pediculus humanus mitochondrial phylogenies. [file 1745-6150-6-55-S4.PDF]

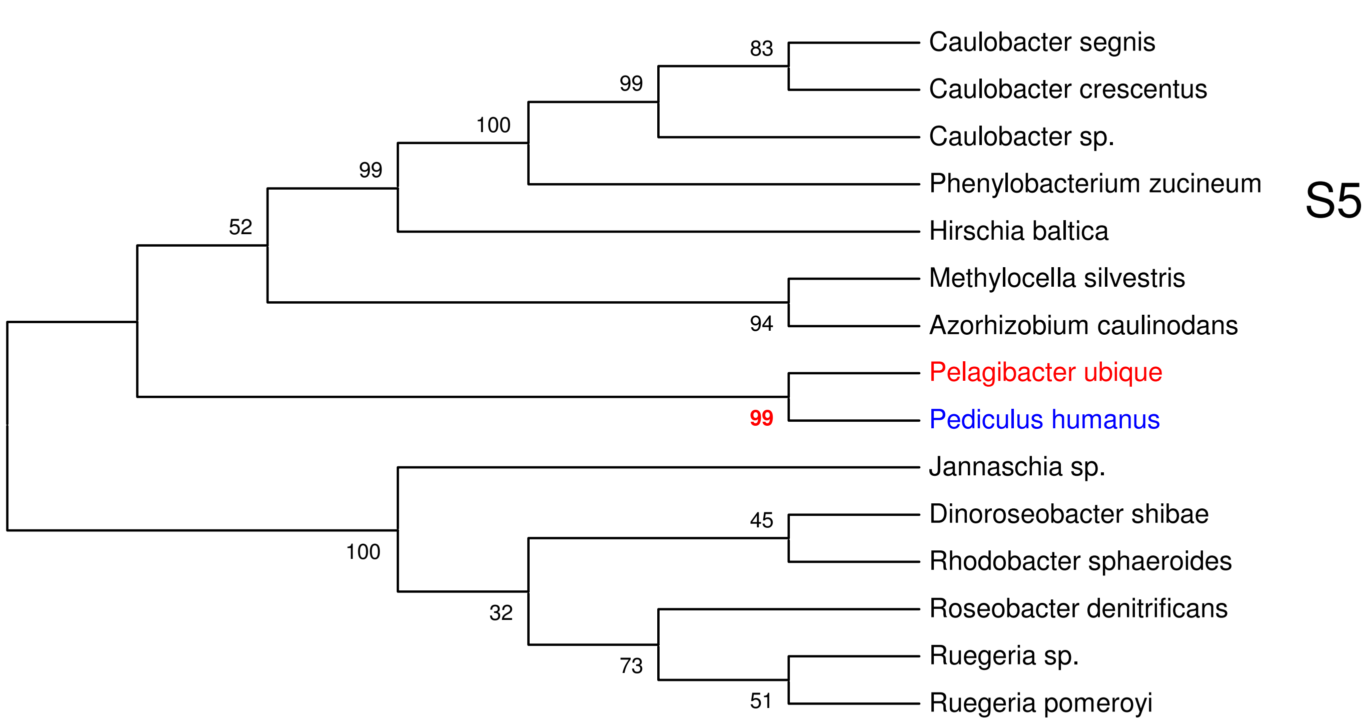
S5
83
99
100
99
52
94
99
100
45
32
73
51
Caulobacter segnis
Caulobacter crescentus
Caulobacter sp.
Phenylobacterium zucineum
Hirschia baltica
Methylocella silvestris
Azorhizobium caulinodans
Pelagibacter ubique
Pediculus humanus
Jannaschia sp.
Dinoroseobacter shibae
Rhodobacter sphaeroides
Roseobacter denitrificans
Ruegeria sp.
Ruegeria pomeroyi

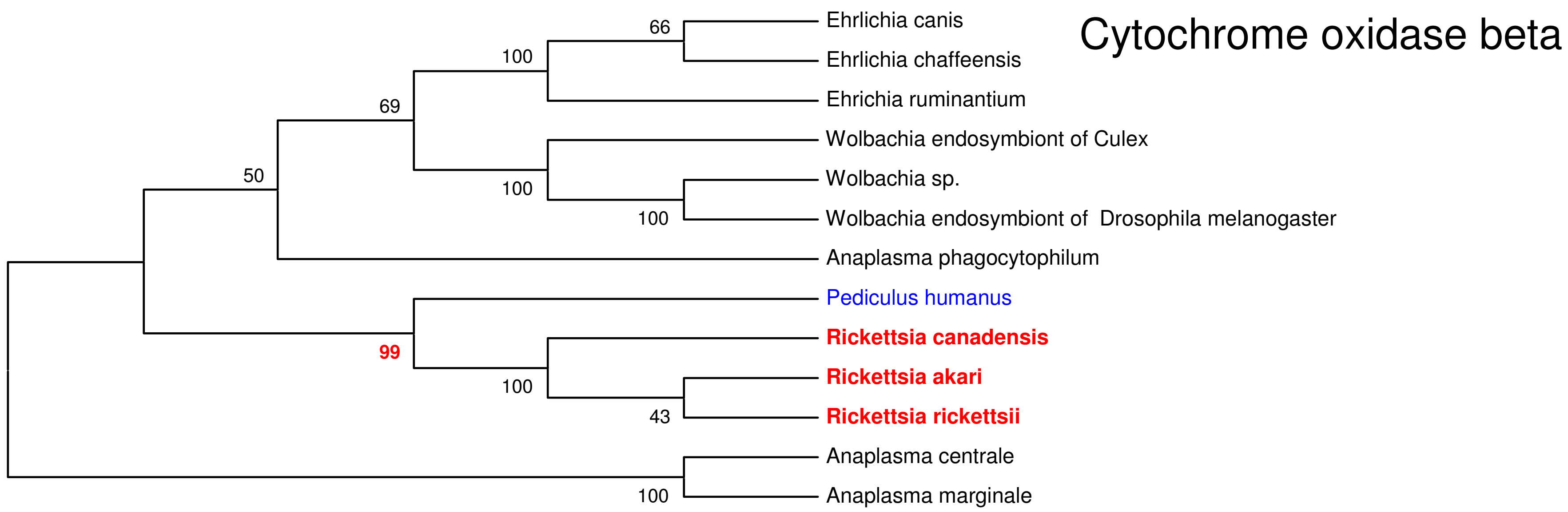
Cytochrome oxidase beta
66
100
69
100
100
50
99
100
43
100
Ehrlichia canis
Ehrlichia chaffeensis
Ehrichia ruminantium
Wolbachia endosymbiont of Culex
Wolbachia sp.
Wolbachia endosymbiont of Drosophila melanogaster
Anaplasma phagocytophilum
Pediculus humanus
Rickettsia canadensis
Rickettsia akari
Rickettsia rickettsii
Anaplasma centrale
Anaplasma marginale

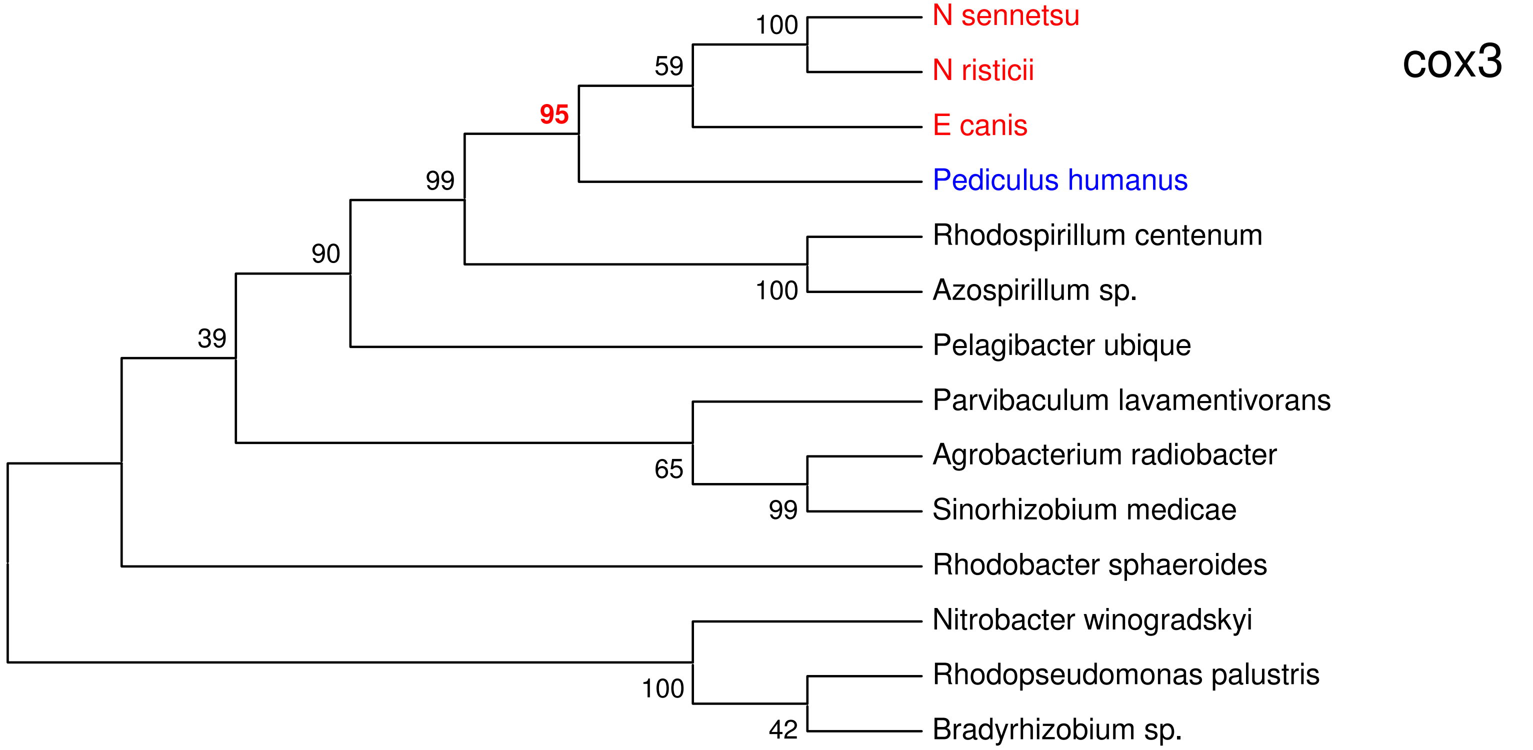
cox3
N sennetsu
N risticii
E canis
Pediculus humanus
Rhodospirillum centenum
Azospirillum sp.
Pelagibacter ubique
Parvibaculum lavamentivorans
Agrobacterium radiobacter
Sinorhizobium medicae
Rhodobacter sphaeroides
Nitrobacter winogradskyi
Rhodopseudomonas palustris
Bradyrhizobium sp.
100
59
95
99
90
39
100
65
99
100
42

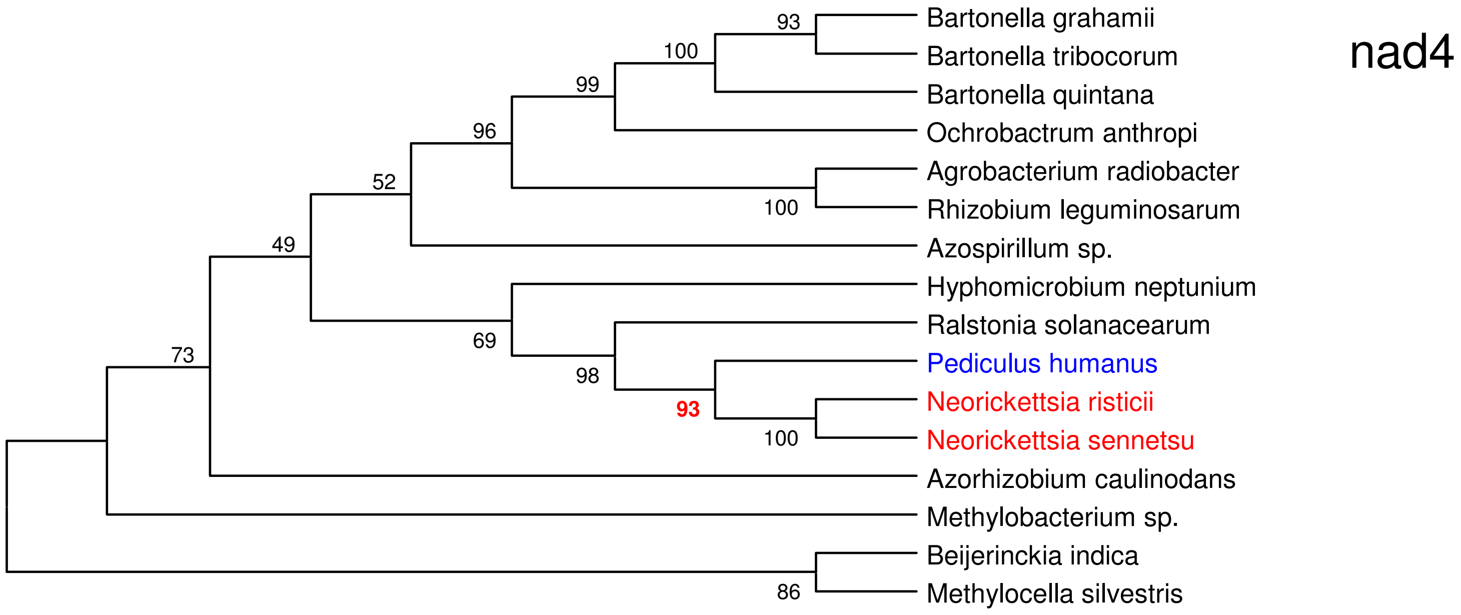
nad4
Bartonella grahamii
Bartonella tribocorum
Bartonella quintana
Ochrobactrum anthropi
Agrobacterium radiobacter
Rhizobium leguminosarum
Azospirillum sp.
Hyphomicrobium neptunium
Ralstonia solanacearum
Pediculus humanus
Neorickettsia risticii
Neorickettsia sennetsu
Azorhizobium caulinodans
Methylobacterium sp.
Beijerinckia indica
Methylocella silvestris
93
100
99
96
52
100
49
69
98
73
93
100
86

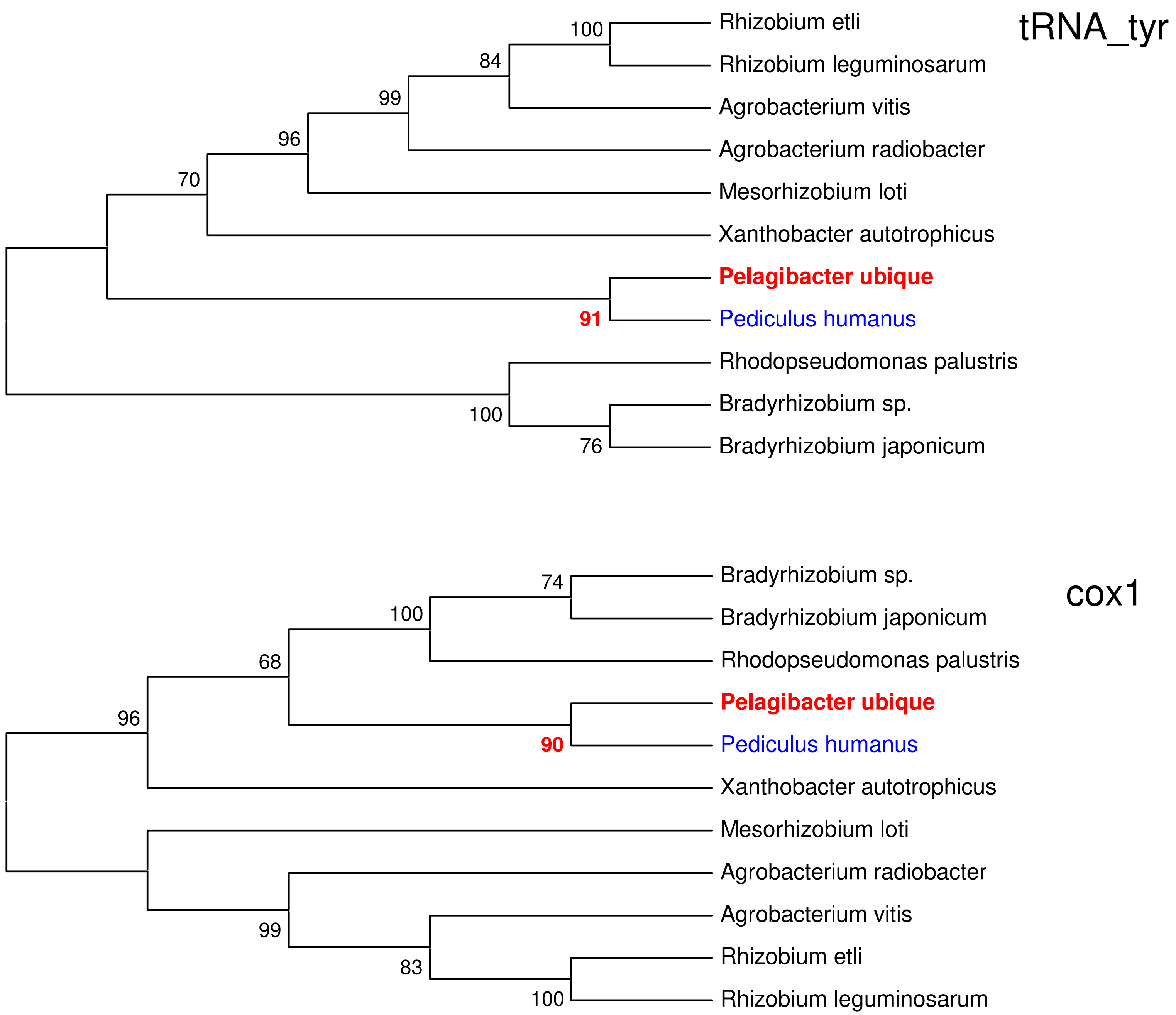

tRNA_tyr
Rhizobium etli
Rhizobium leguminosarum
Agrobacterium vitis
Agrobacterium radiobacter
Mesorhizobium loti
Xanthobacter autotrophicus
Pelagibacter ubique
Pediculus humanus
Rhodopseudomonas palustris
Bradyrhizobium sp.
Bradyrhizobium japonicum
100
84
99
96
70
91
100
76
cox1
Bradyrhizobium sp.
Bradyrhizobium japonicum
Rhodopseudomonas palustris
Pelagibacter ubique
Pediculus humanus
Xanthobacter autotrophicus
Mesorhizobium loti
Agrobacterium radiobacter
Agrobacterium vitis
Rhizobium etli
Rhizobium leguminosarum
74
100
68
96
90
99
83
100

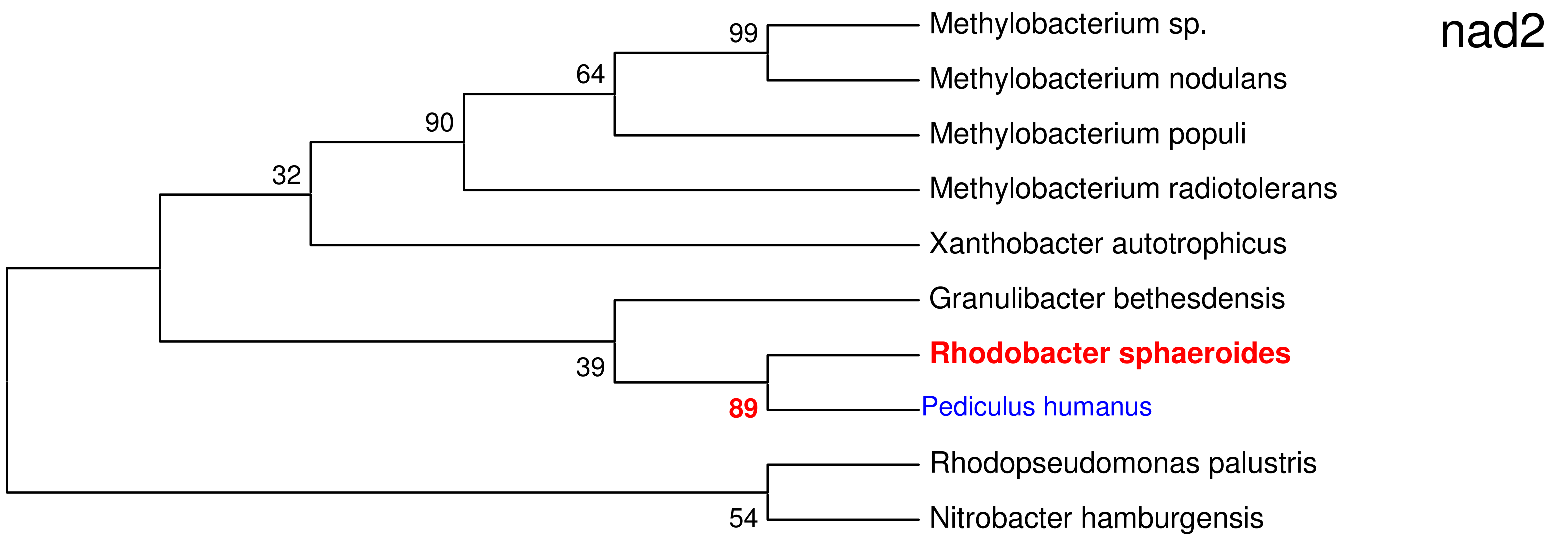
nad2
99
64
90
32
39
89
54
Methylobacterium sp.
Methylobacterium nodulans
Methylobacterium populi
Methylobacterium radiotolerans
Xanthobacter autotrophicus
Granulibacter bethesdensis
Rhodobacter sphaeroides
Pediculus humanus
Rhodopseudomonas palustris
Nitrobacter hamburgensis

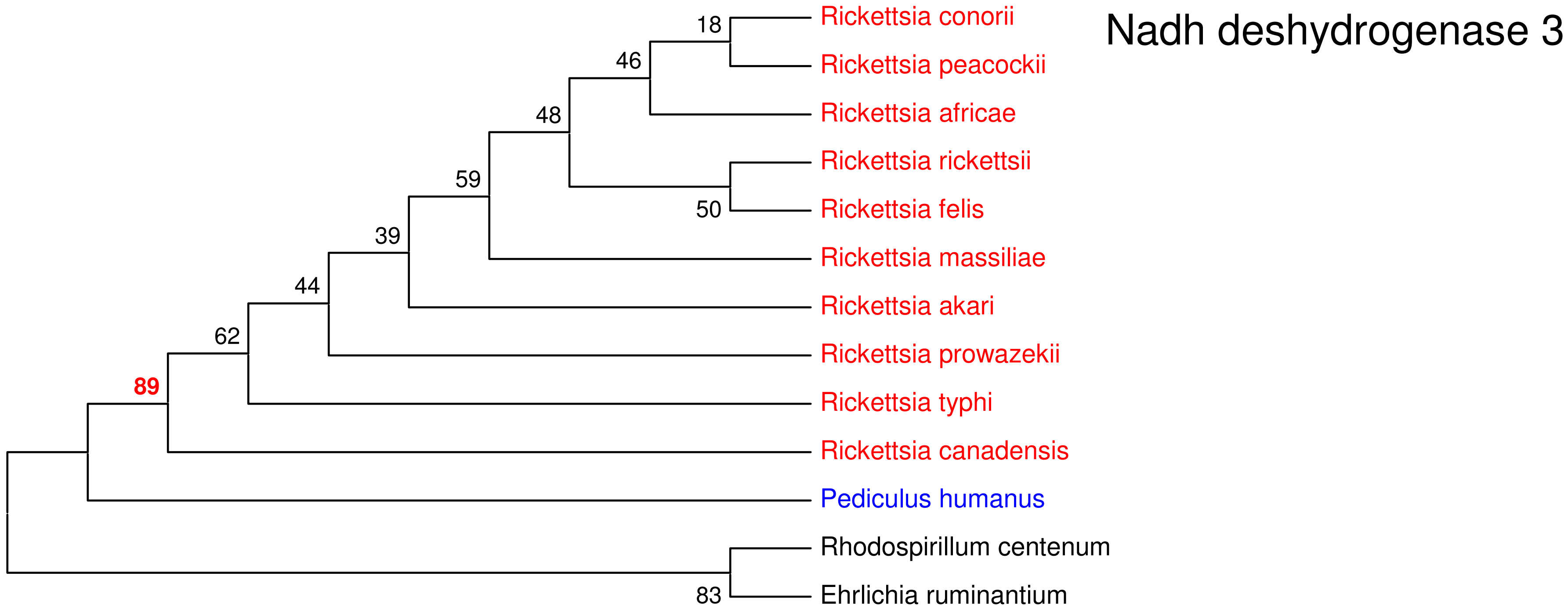
Nadh deshydrogenase 3
18
46
48
59
39
44
62
89
50
83
Rickettsia conorii
Rickettsia peacockii
Rickettsia africae
Rickettsia rickettsii
Rickettsia felis
Rickettsia massiliae
Rickettsia akari
Rickettsia prowazekii
Rickettsia typhi
Rickettsia canadensis
Pediculus humanus
Rhodospirillum centenum
Ehrlichia ruminantium

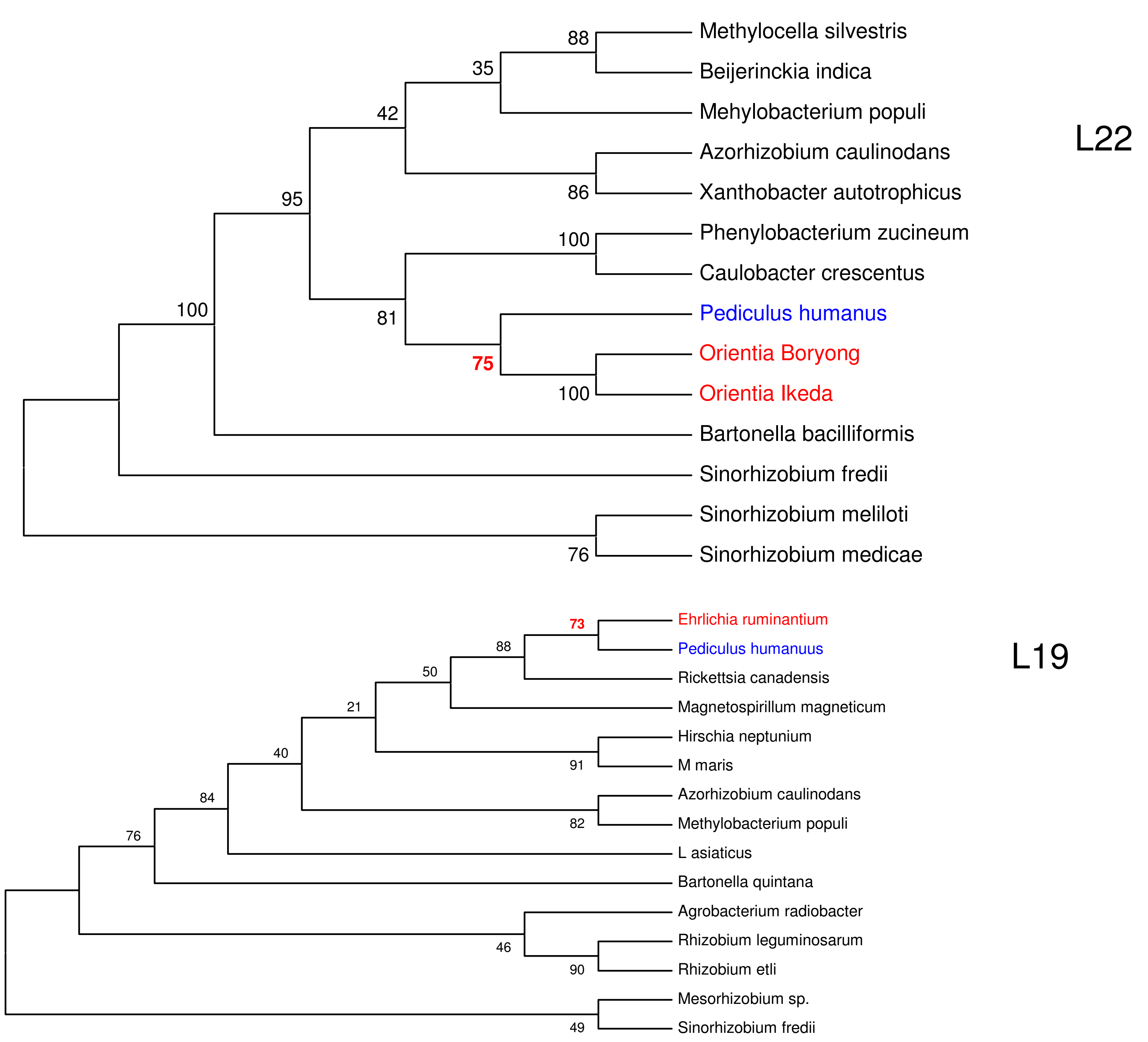

L22
Methylocella silvestris
Beijerinckia indica
Mehylobacterium populi
Azorhizobium caulinodans
Xanthobacter autotrophicus
Phenylobacterium zucineum
Caulobacter crescentus
Pediculus humanus
Orientia Boryong
Orientia Ikeda
Bartonella bacilliformis
Sinorhizobium fredii
Sinorhizobium meliloti
Sinorhizobium medicae
88
35
42
86
95
100
100
81
75
100
76
L19
Ehrlichia ruminantium
Pediculus humanuus
Rickettsia canadensis
Magnetospirillum magneticum
Hirschia neptunium
M maris
Azorhizobium caulinodans
Methylobacterium populi
L asiaticus
Bartonella quintana
Agrobacterium radiobacter
Rhizobium leguminosarum
Rhizobium etli
Mesorhizobium sp.
Sinorhizobium fredii
73
88
50
21
91
40
82
84
76
46
90
49

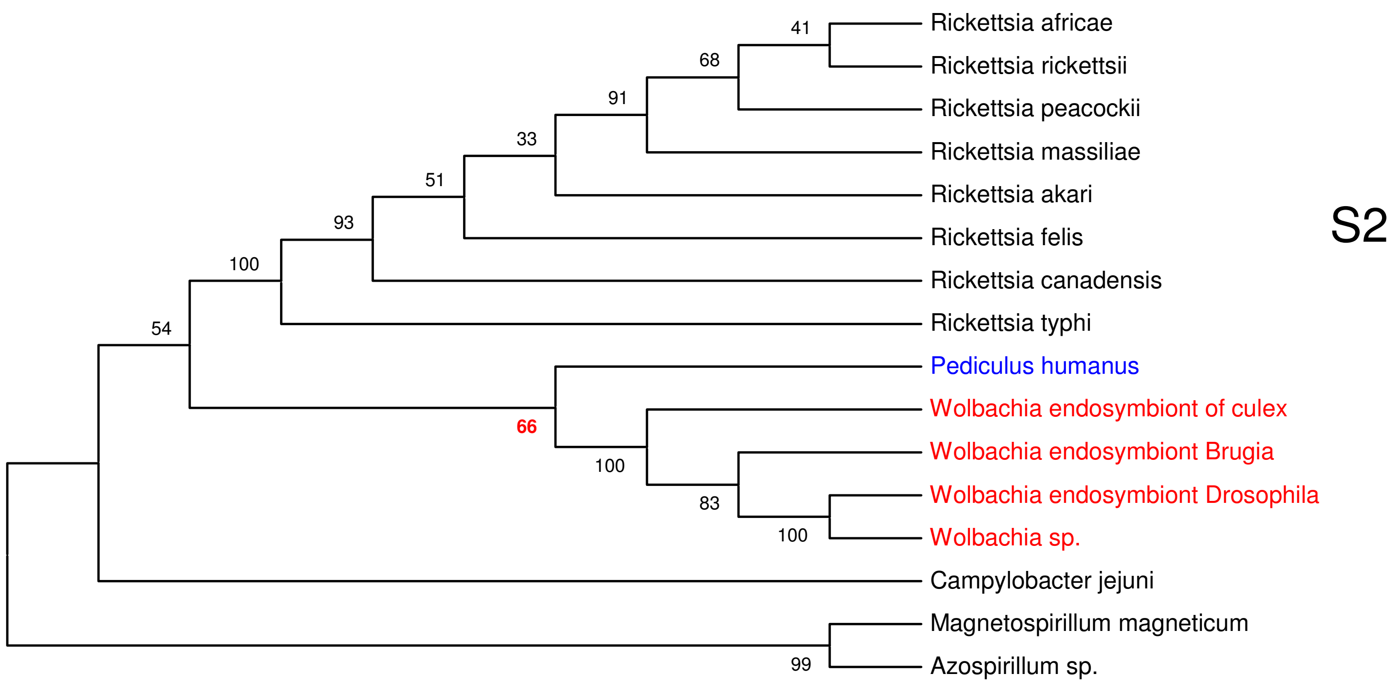
S2
Rickettsia africae
Rickettsia rickettsii
Rickettsia peacockii
Rickettsia massiliae
Rickettsia akari
Rickettsia felis
Rickettsia canadensis
Rickettsia typhi
Pediculus humanus
Wolbachia endosymbiont of culex
Wolbachia endosymbiont Brugia
Wolbachia endosymbiont Drosophila
Wolbachia sp.
Campylobacter jejuni
Magnetospirillum magneticum
Azospirillum sp.
41
68
91
33
51
93
100
54
66
100
83
100
99

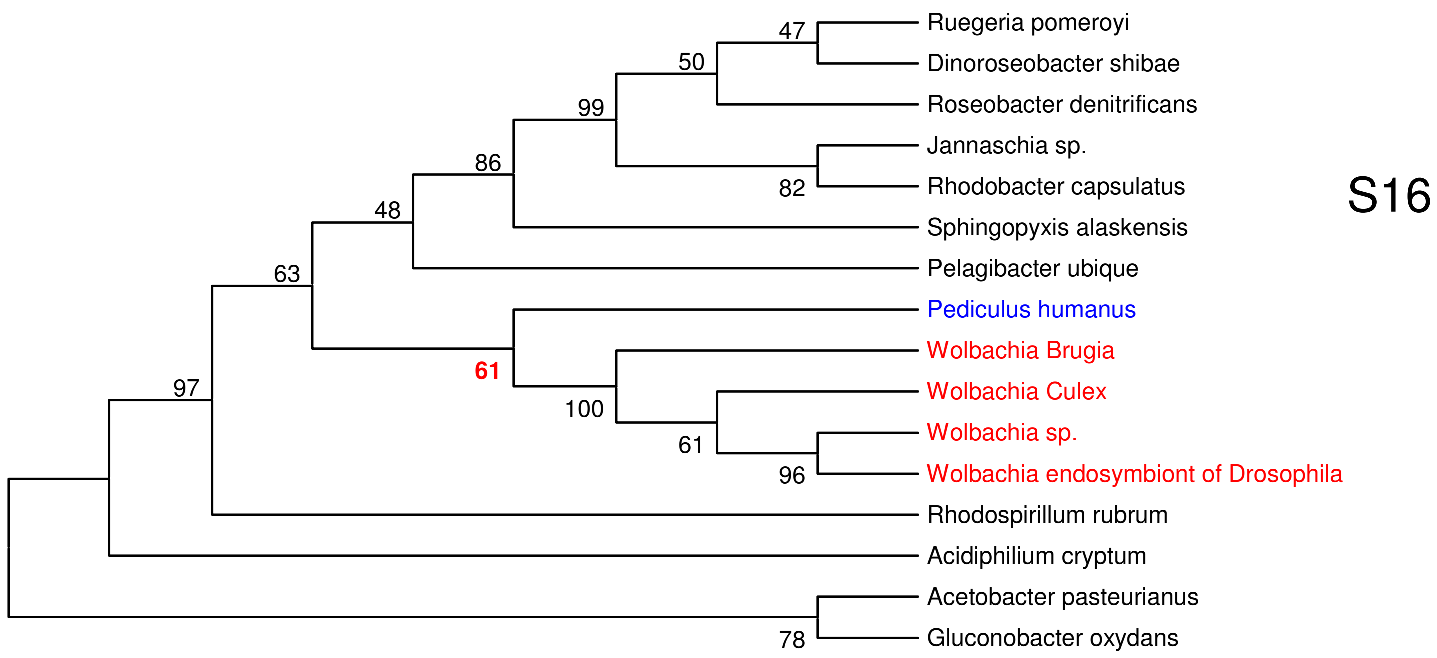
S16
Ruegeria pomeroyi
Dinoroseobacter shibae
Roseobacter denitrificans
Jannaschia sp.
Rhodobacter capsulatus
Sphingopyxis alaskensis
Pelagibacter ubique
Pediculus humanus
Wolbachia Brugia
Wolbachia Culex
Wolbachia sp.
Wolbachia endosymbiont of Drosophila
Rhodospirillum rubrum
Acidiphilium cryptum
Acetobacter pasteurianus
Gluconobacter oxydans
47
50
99
82
86
48
63
61
100
61
96
97
78

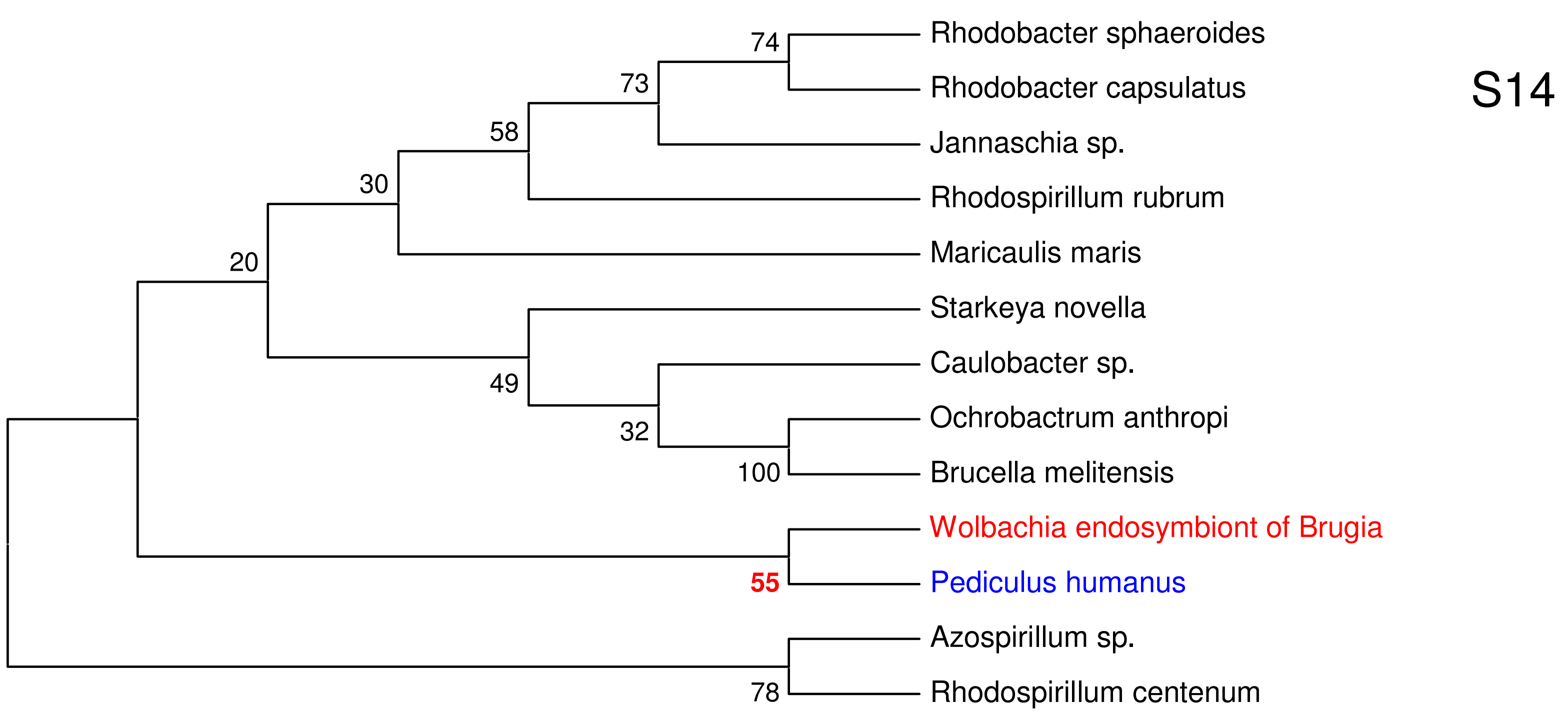
S14
Rhodobacter sphaeroides
Rhodobacter capsulatus
Jannaschia sp.
Rhodospirillum rubrum
Maricaulis maris
Starkeya novella
Caulobacter sp.
Ochrobactrum anthropi
Brucella melitensis
Wolbachia endosymbiont of Brugia
Pediculus humanus
Azospirillum sp.
Rhodospirillum centenum
74
73
58
30
20
49
32
100
55
78

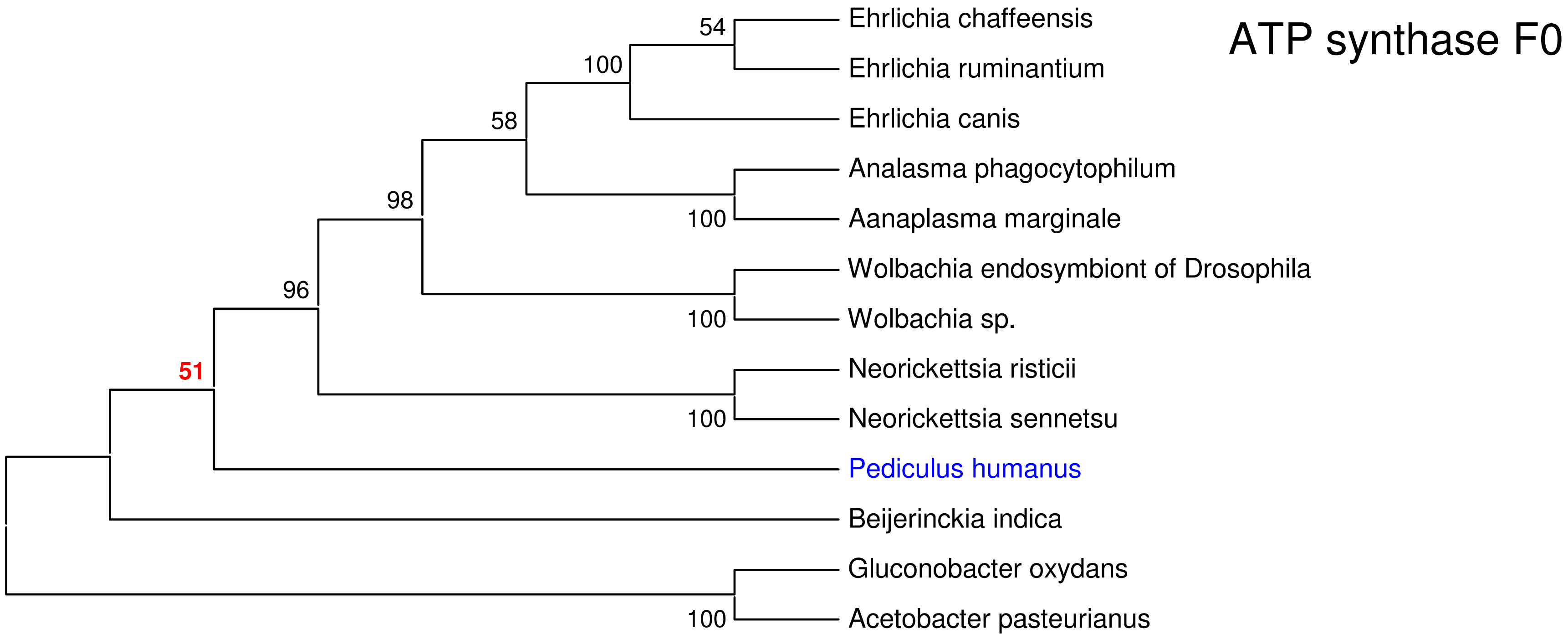
ATP synthase F0
Ehrlichia chaffeensis
Ehrlichia ruminantium
Ehrlichia canis
Analasma phagocytophilum
Aanaplasma marginale
Wolbachia endosymbiont of Drosophila
Wolbachia sp.
Neorickettsia risticii
Neorickettsia sennetsu
Pediculus humanus
Beijerinckia indica
Gluconobacter oxydans
Acetobacter pasteurianus
54
100
58
98
96
51
100
100
100
100

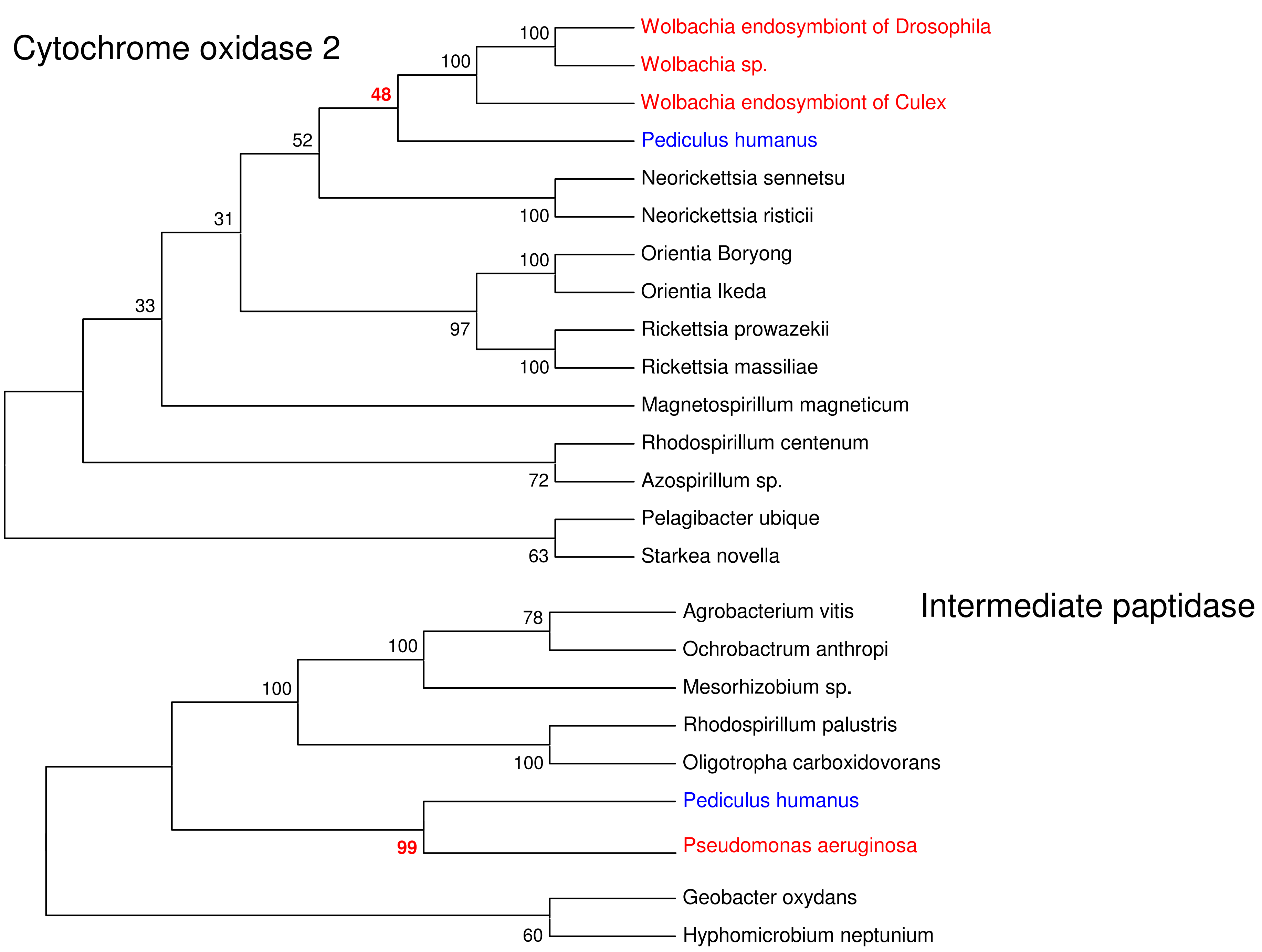

Cytochrome oxidase 2
Wolbachia endosymbiont of Drosophila
Wolbachia sp.
Wolbachia endosymbiont of Culex
Pediculus humanus
Neorickettsia sennetsu
Neorickettsia risticii
Orientia Boryong
Orientia Ikeda
Rickettsia prowazekii
Rickettsia massiliae
Magnetospirillum magneticum
Rhodospirillum centenum
Azospirillum sp.
Pelagibacter ubique
Starkea novella
100
100
48
52
100
31
100
97
100
33
72
63
Intermediate paptidase
Agrobacterium vitis
Ochrobactrum anthropi
Mesorhizobium sp.
Rhodospirillum palustris
Oligotropha carboxidovorans
Pediculus humanus
Pseudomonas aeruginosa
Geobacter oxydans
Hyphomicrobium neptunium
78
100
100
100
99
60

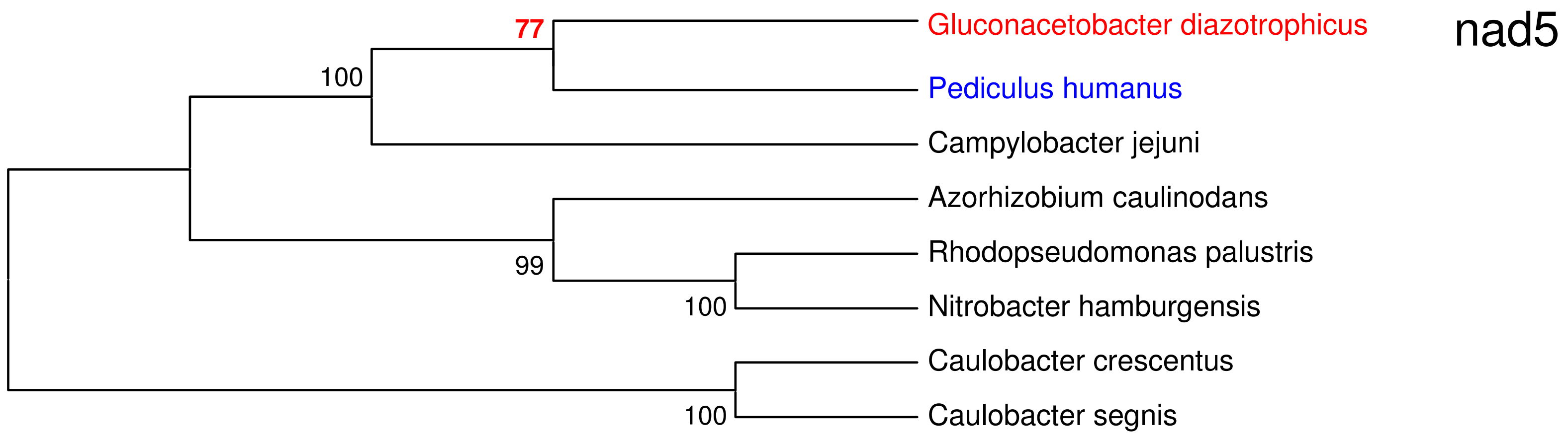
nad5
77
100
99
100
100
Gluconacetobacter diazotrophicus
Pediculus humanus
Campylobacter jejuni
Azorhizobium caulinodans
Rhodopseudomonas palustris
Nitrobacter hamburgensis
Caulobacter crescentus
Caulobacter segnis

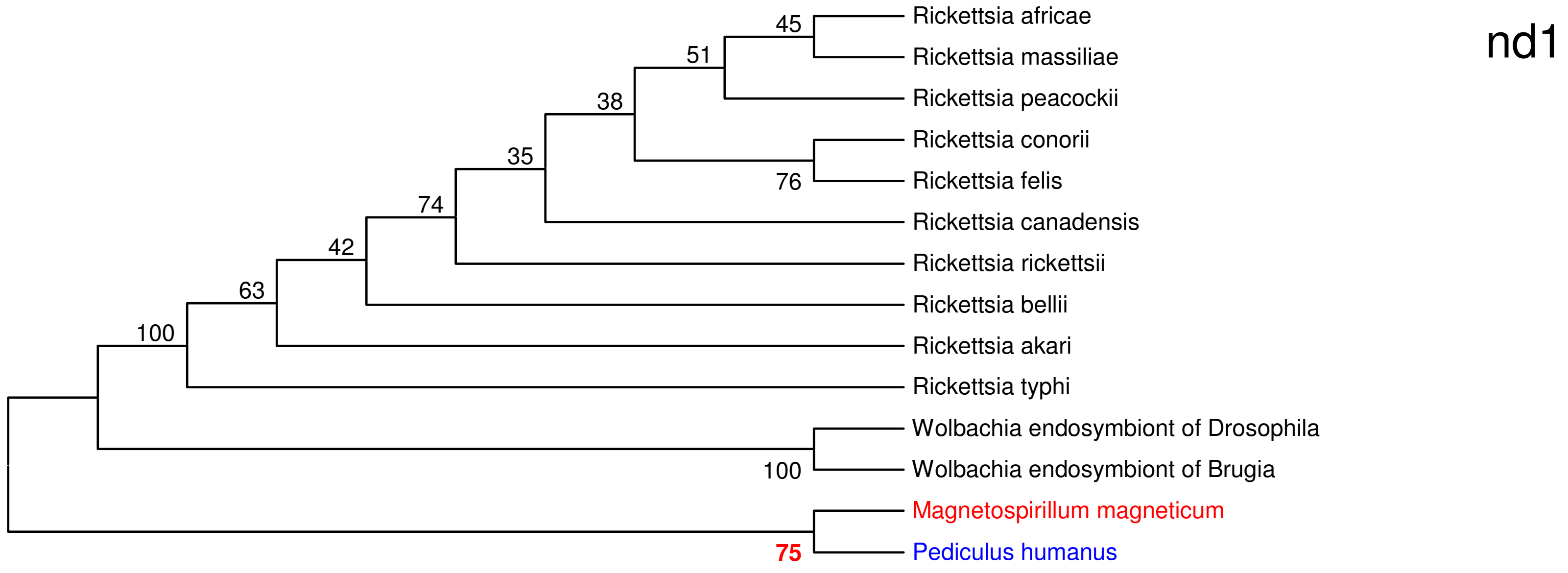
nd1
45
51
38
76
35
74
42
63
100
100
75
Rickettsia africae
Rickettsia massiliae
Rickettsia peacockii
Rickettsia conorii
Rickettsia felis
Rickettsia canadensis
Rickettsia rickettsii
Rickettsia bellii
Rickettsia akari
Rickettsia typhi
Wolbachia endosymbiont of Drosophila
Wolbachia endosymbiont of Brugia
Magnetospirillum magneticum
Pediculus humanus

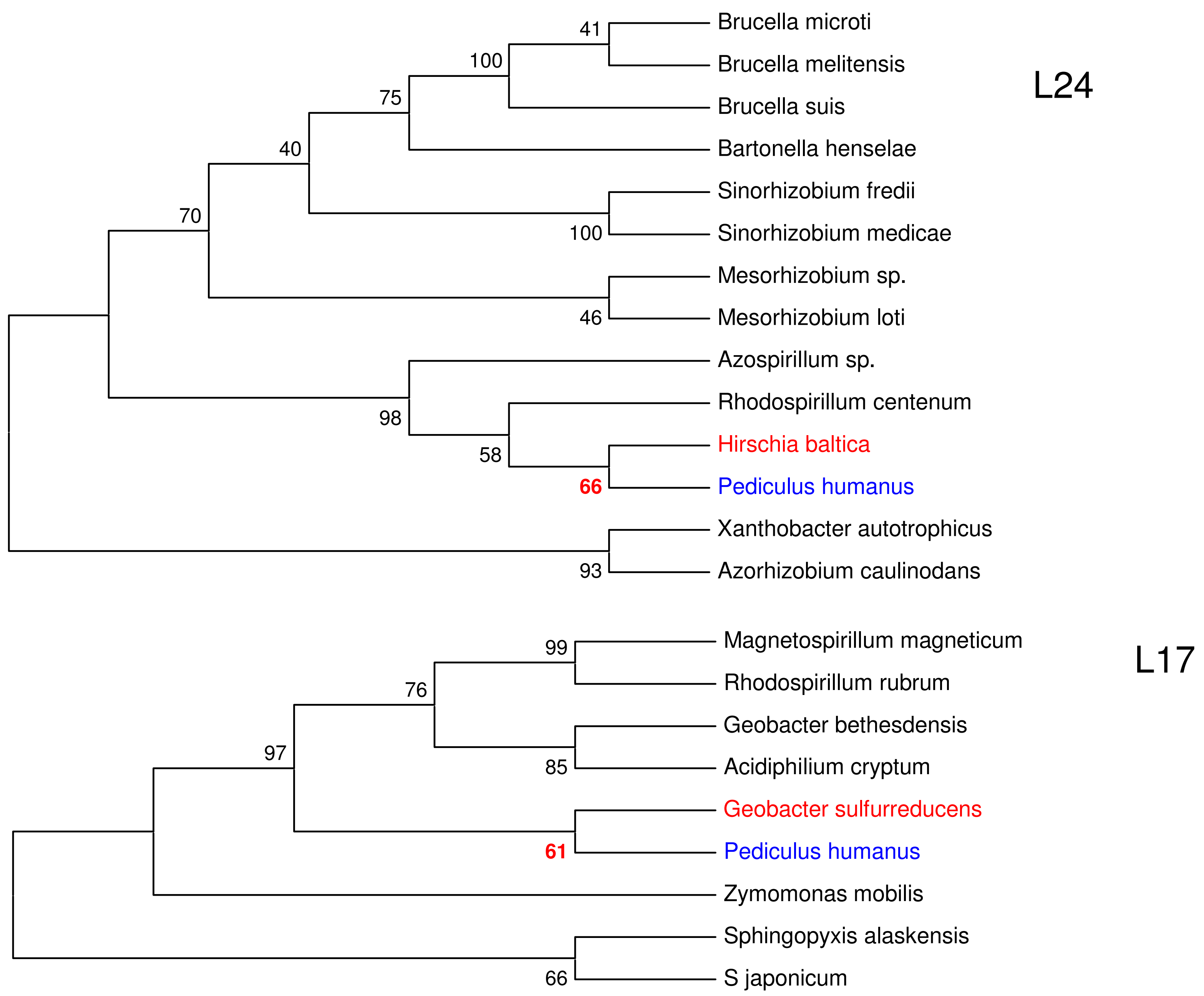
L24
Brucella microti
Brucella melitensis
Brucella suis
Bartonella henselae
Sinorhizobium fredii
Sinorhizobium medicae
Mesorhizobium sp.
Mesorhizobium loti
Azospirillum sp.
Rhodospirillum centenum
Hirschia baltica
Pediculus humanus
Xanthobacter autotrophicus
Azorhizobium caulinodans
41
100
75
40
70
100
46
98
58
66
93
L17
Magnetospirillum magneticum
Rhodospirillum rubrum
Geobacter bethesdensis
Acidiphilium cryptum
Geobacter sulfurreducens
Pediculus humanus
Zymomonas mobilis
Sphingopyxis alaskensis
S japonicum
99
76
97
85
61
66

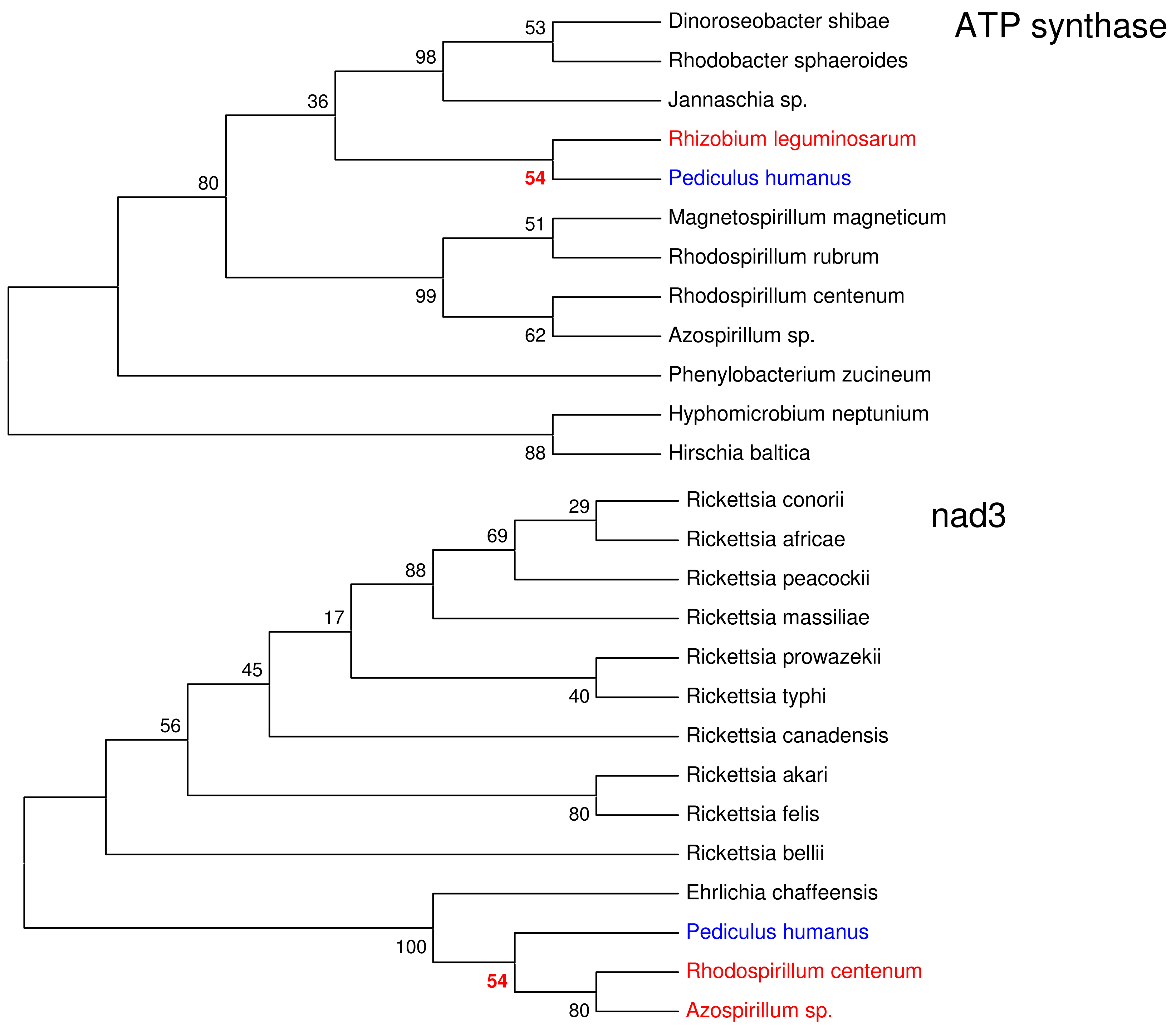
ATP synthase
Dinoroseobacter shibae
Rhodobacter sphaeroides
Jannaschia sp.
Rhizobium leguminosarum
Pediculus humanus
Magnetospirillum magneticum
Rhodospirillum rubrum
Rhodospirillum centenum
Azospirillum sp.
Phenylobacterium zucineum
Hyphomicrobium neptunium
Hirschia baltica
53
98
36
54
80
51
99
62
88
nad3
Rickettsia conorii
Rickettsia africae
Rickettsia peacockii
Rickettsia massiliae
Rickettsia prowazekii
Rickettsia typhi
Rickettsia canadensis
Rickettsia akari
Rickettsia felis
Rickettsia bellii
Ehrlichia chaffeensis
Pediculus humanus
Rhodospirillum centenum
Azospirillum sp.
29
69
88
17
45
40
56
80
100
54
80

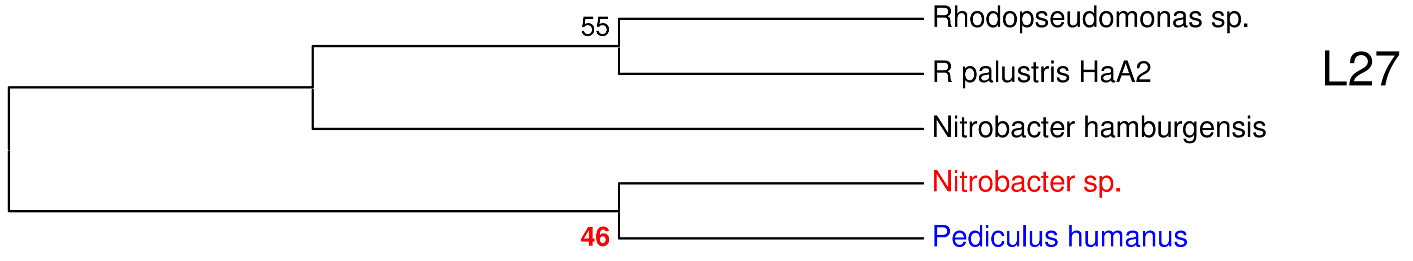
55
Rhodopseudomonas sp.
R palustris HaA2
L27
Nitrobacter hamburgensis
Nitrobacter sp.
46
Pediculus humanus

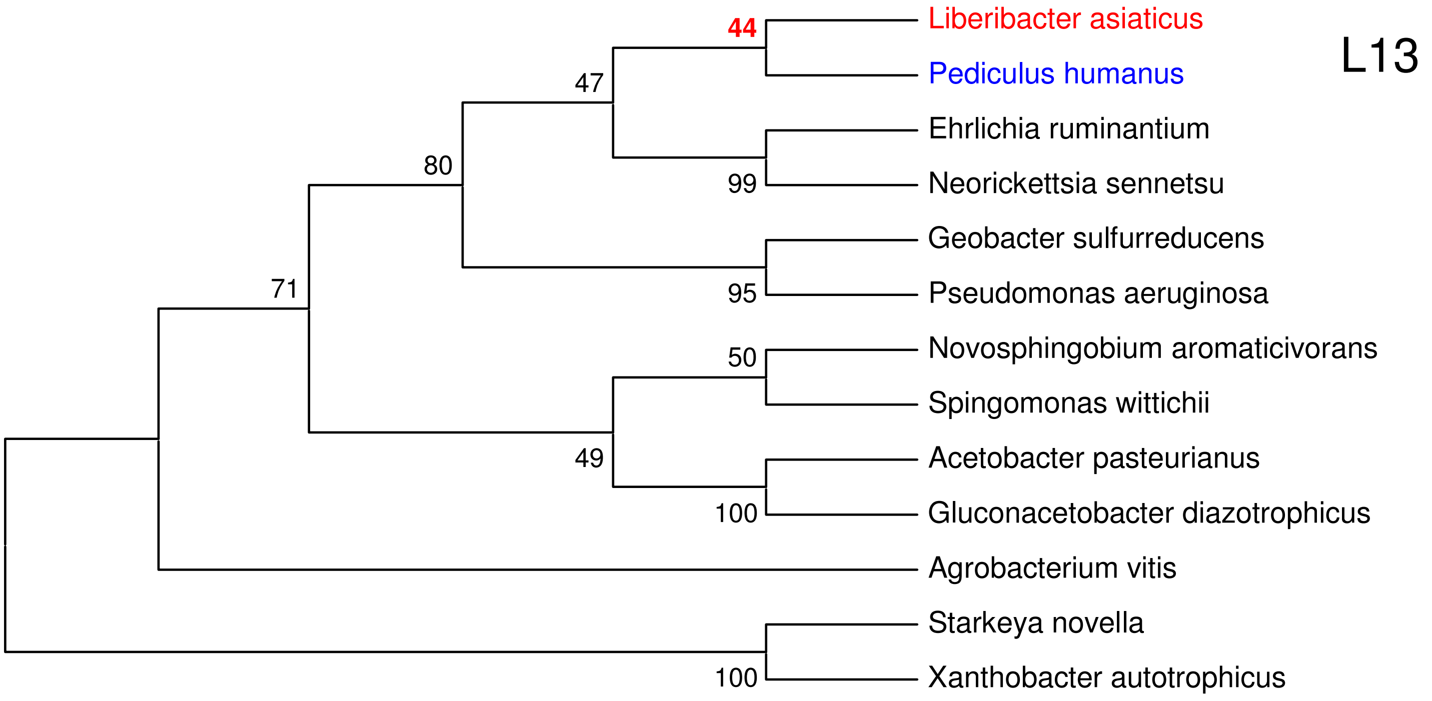
44
Liberibacter asiaticus
Pediculus humanus
L13
47
Ehrlichia ruminantium
80
99
Neorickettsia sennetsu
Geobacter sulfurreducens
71
95
Pseudomonas aeruginosa
50
Novosphingobium aromaticivorans
Spingomonas wittichii
49
Acetobacter pasteurianus
100
Gluconacetobacter diazotrophicus
Agrobacterium vitis
Starkeya novella
100
Xanthobacter autotrophicus

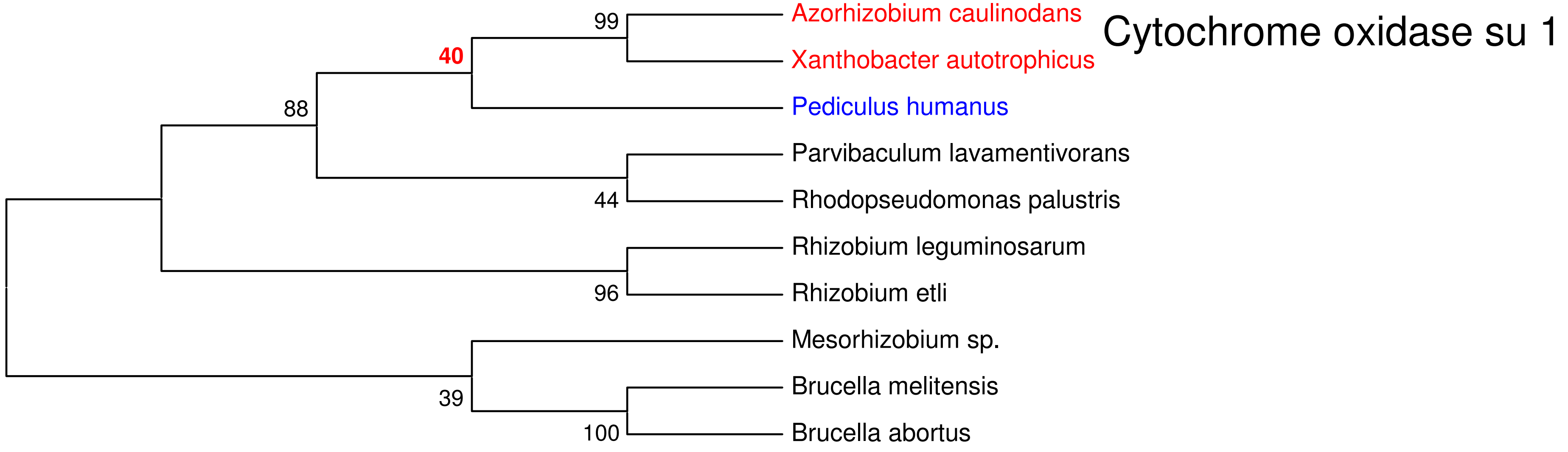
Cytochrome oxidase su 1
Azorhizobium caulinodans
Xanthobacter autotrophicus
Pediculus humanus
Parvibaculum lavamentivorans
Rhodopseudomonas palustris
Rhizobium leguminosarum
Rhizobium etli
Mesorhizobium sp.
Brucella melitensis
Brucella abortus
99
40
88
44
96
39
100

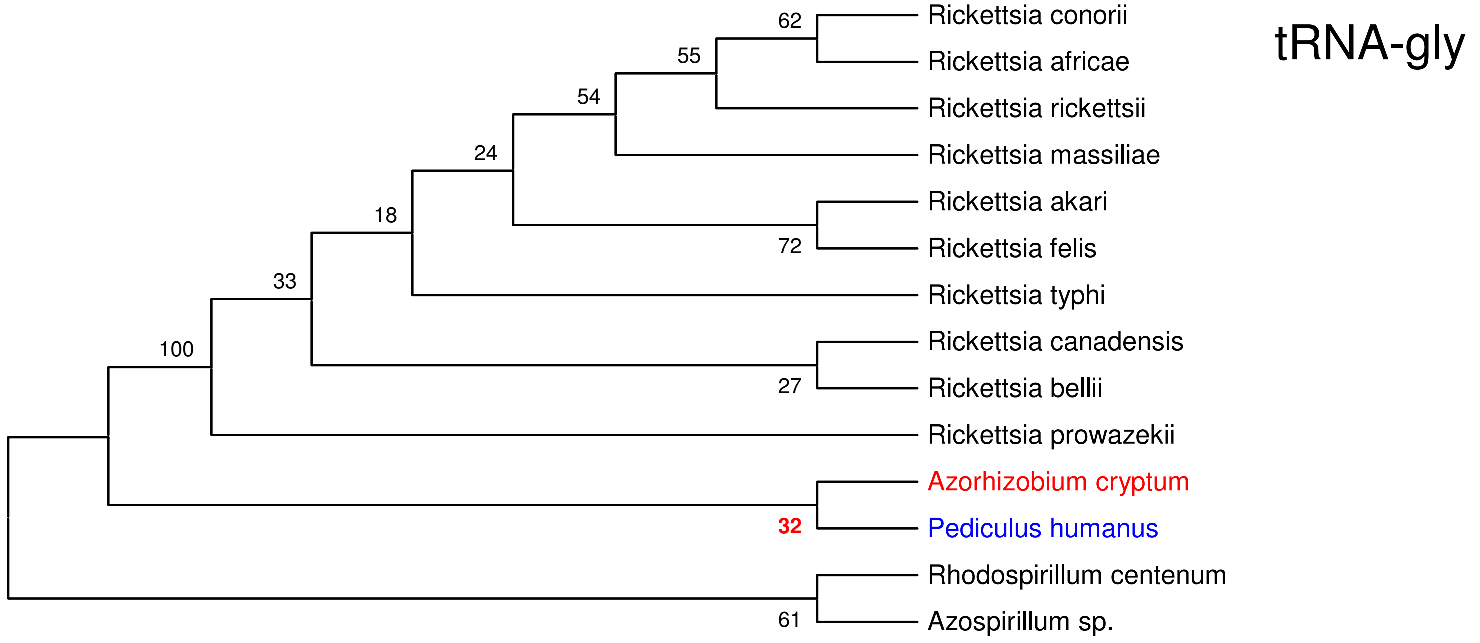
tRNA-gly
Rickettsia conorii
Rickettsia africae
Rickettsia rickettsii
Rickettsia massiliae
Rickettsia akari
Rickettsia felis
Rickettsia typhi
Rickettsia canadensis
Rickettsia bellii
Rickettsia prowazekii
Azorhizobium cryptum
Pediculus humanus
Rhodospirillum centenum
Azospirillum sp.
62
55
54
24
18
33
100
72
27
32
61
