# Supplementary material for: The rhizome of Reclinomonas americana, Homo sapiens, Pediculus humanus and Saccharomyces cerevisiae mitochondria
Source: Biol Direct. 2011 Oct 20;6:55. doi: 10.1186/1745-6150-6-55 (PMC3214132; doi:10.1186/1745-6150-6-55)
Supplement: Additional file 5 — Previous horizontal gene transfers in Alphaproteobacteria. [file 1745-6150-6-55-S5.PDF]

Ribosomal protein L6

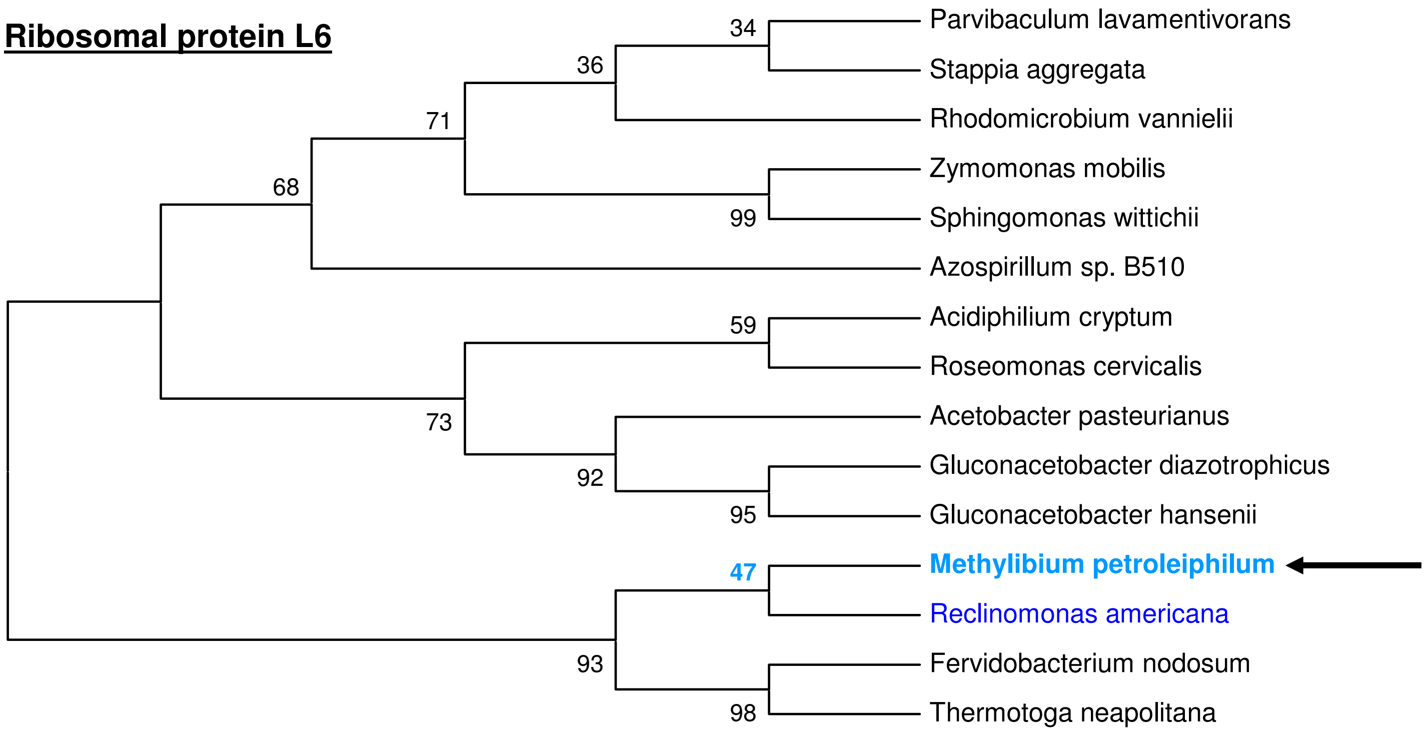

Methylium petroleiphilum gained gene by Betaproteobacteria

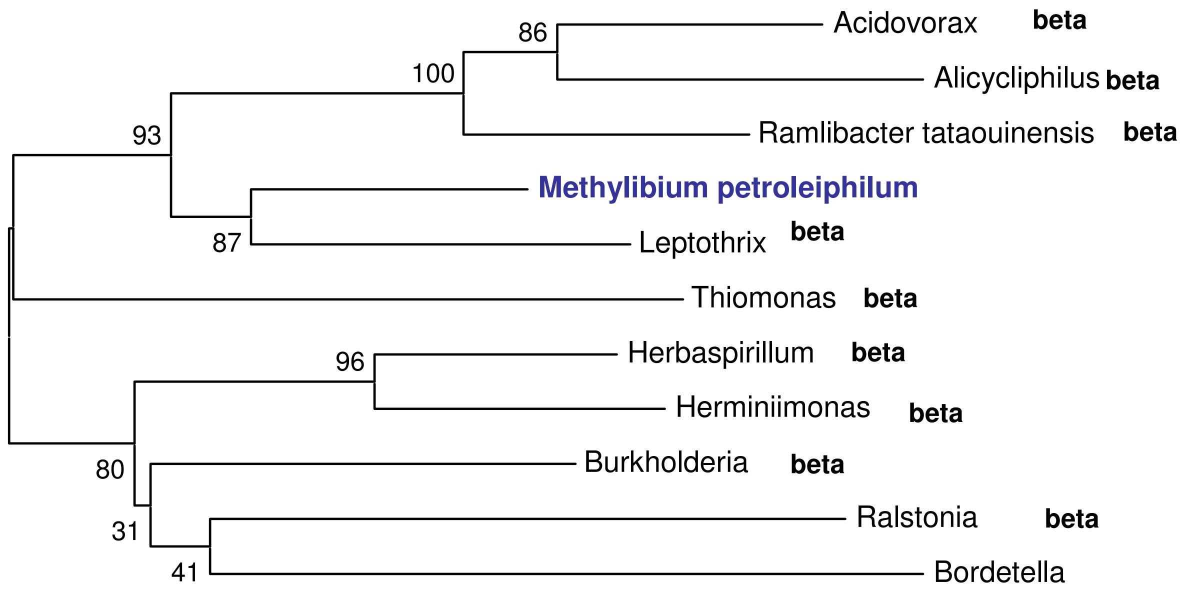

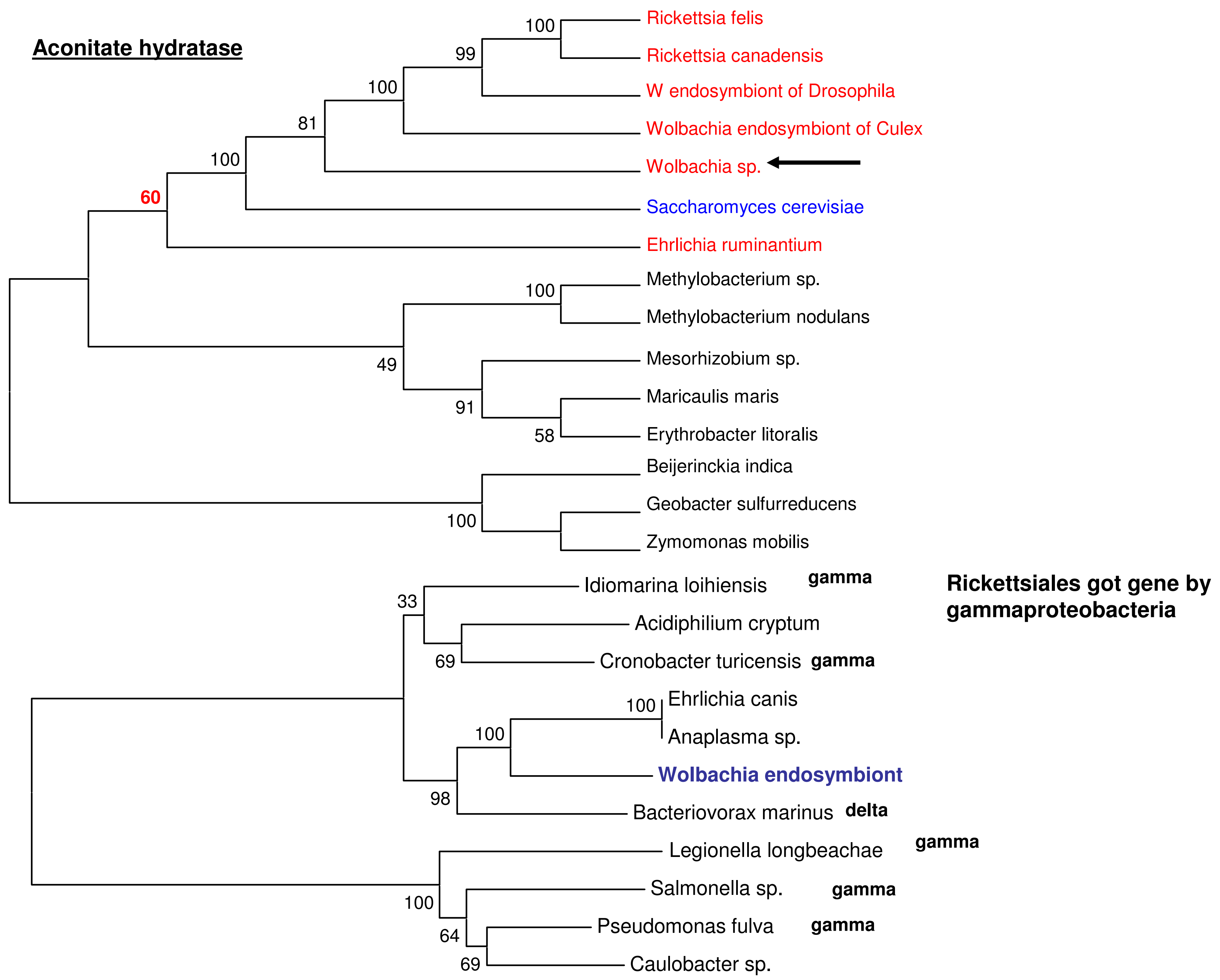

Aconitate hydratase
Rickettsia felis
Rickettsia canadensis
W endosymbiont of Drosophila
Wolbachia endosymbiont of Culex
Wolbachia sp.
Saccharomyces cerevisiae
Ehrlichia ruminantium
Methylobacterium sp.
Methylobacterium nodulans
Mesorhizobium sp.
Maricaulis maris
Erythrobacter litoralis
Beijerinckia indica
Geobacter sulfurreducens
Zymomonas mobilis
100
99
100
81
100
60
100
49
91
58
100
Idiomarina loihiensis
gamma
Rickettsiales got gene by gammaproteobacteria
Acidiphilium cryptum
Cronobacter turicensis
gamma
Ehrlichia canis
Anaplasma sp.
Wolbachia endosymbiont
Bacteriovorax marinus
delta
Legionella longbeachae
gamma
Salmonella sp.
gamma
Pseudomonas fulva
gamma
Caulobacter sp.
33
69
100
100
98
100
64
69
