# Supplementary material for: The rhizome of Reclinomonas americana, Homo sapiens, Pediculus humanus and Saccharomyces cerevisiae mitochondria
Source: Biol Direct. 2011 Oct 20;6:55. doi: 10.1186/1745-6150-6-55 (PMC3214132; doi:10.1186/1745-6150-6-55)

Ribosomal protein L16

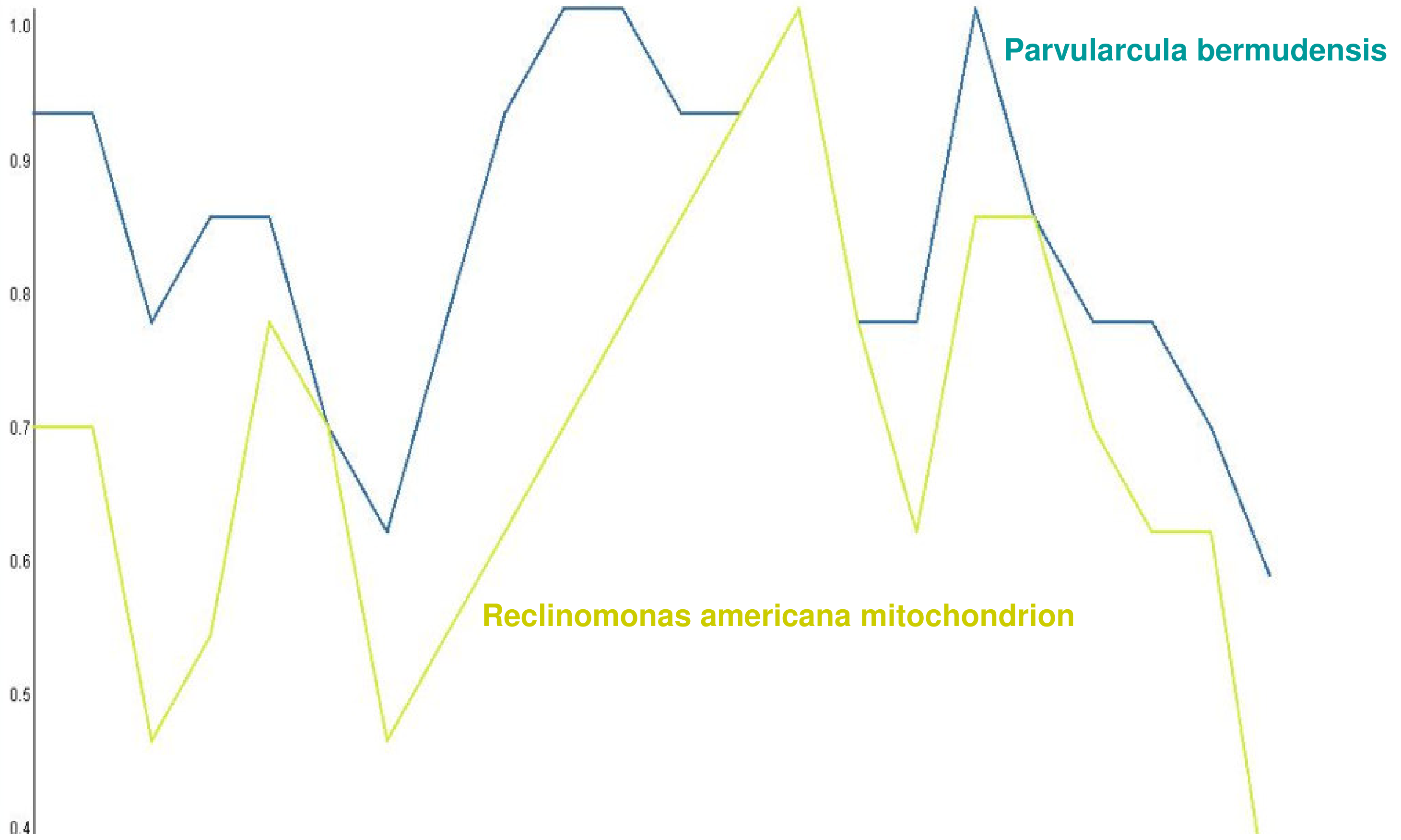

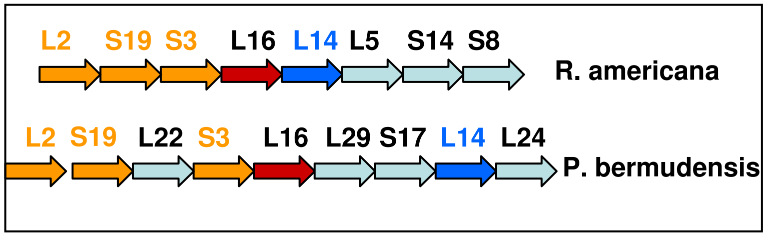

Ribosomal protein L14

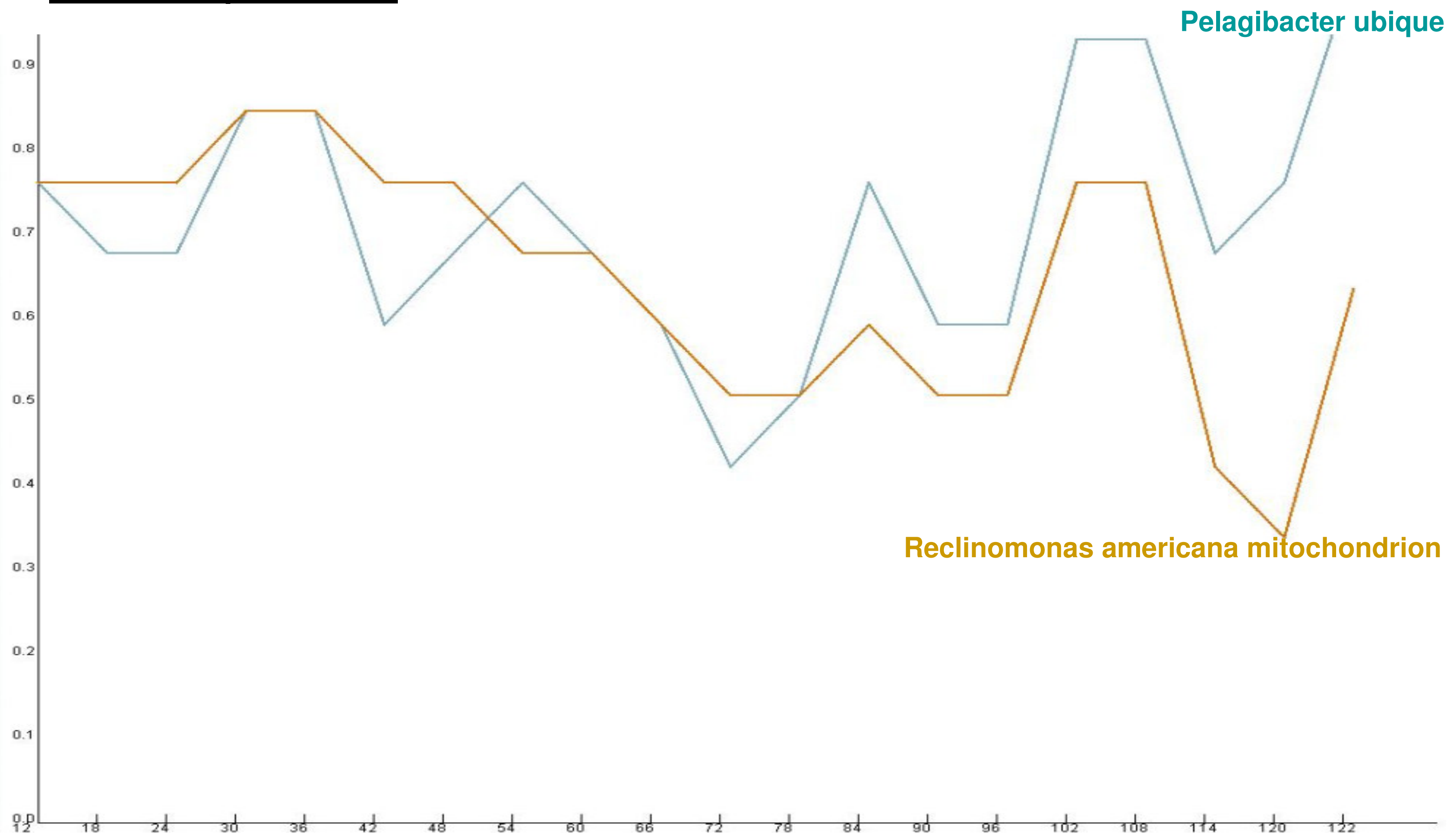

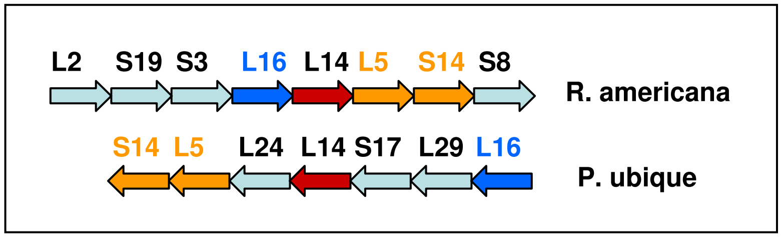

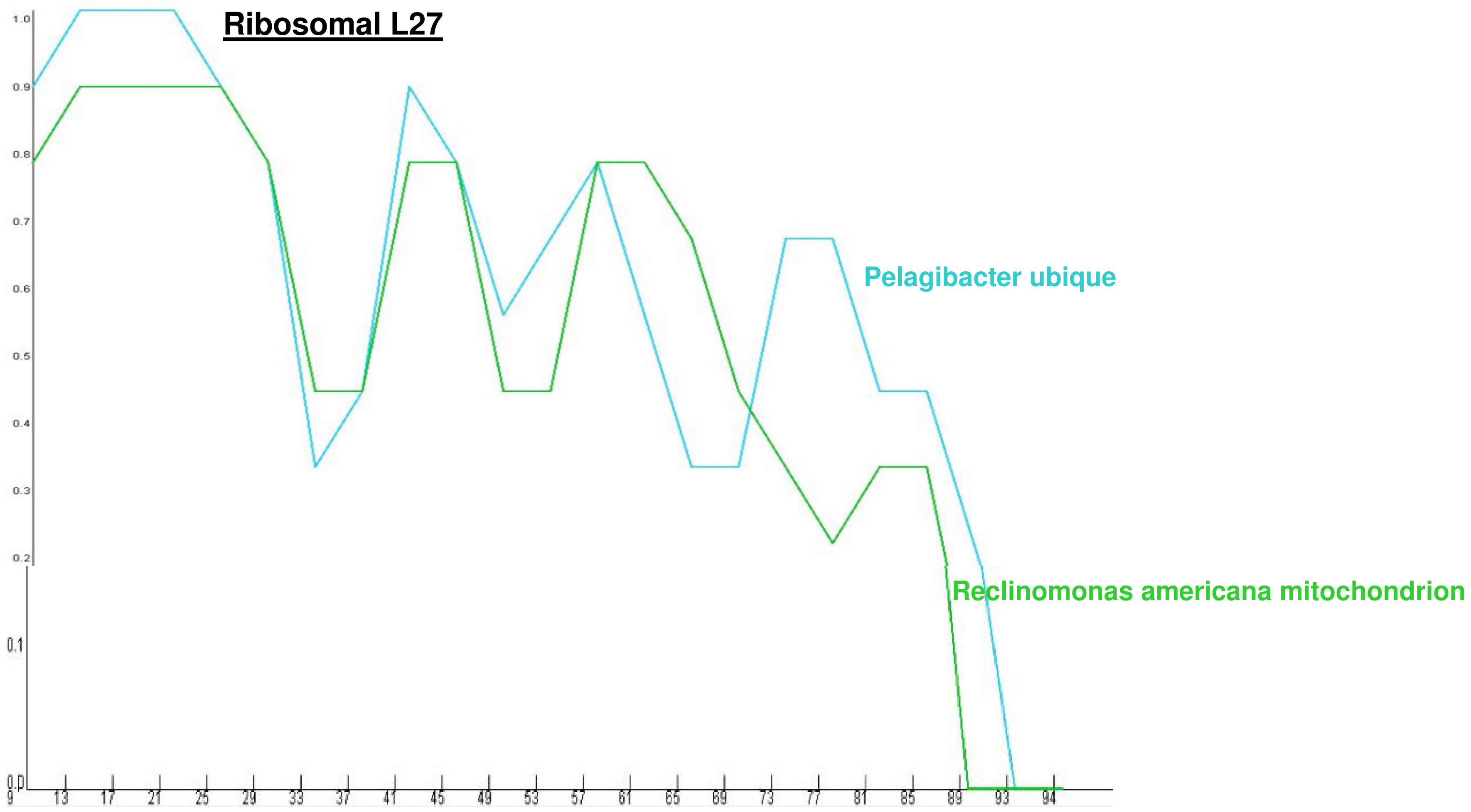
Ribosomal L27
1.0
0.9
0.8
0.7
0.6
0.5
0.4
0.3
0.2
0.1
0.0
9 13 17 21 25 29 33 37 41 45 49 53 57 61 65 69 73 77 81 85 89 93 94
Pelagibacter ubique
Reclinomonas americana mitochondrion

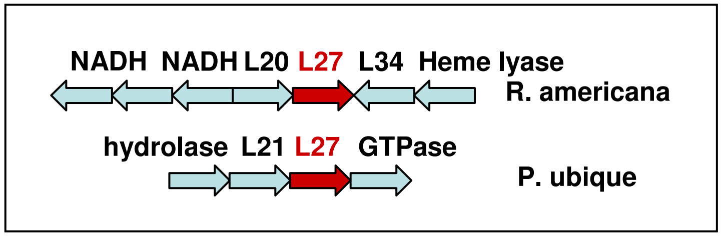
NADH NADH L20 L27 L34 Heme lyase
R. americana
hydrolase L21 L27 GTPase
P. ubique

Succinate ubiquinone oxidoreductase

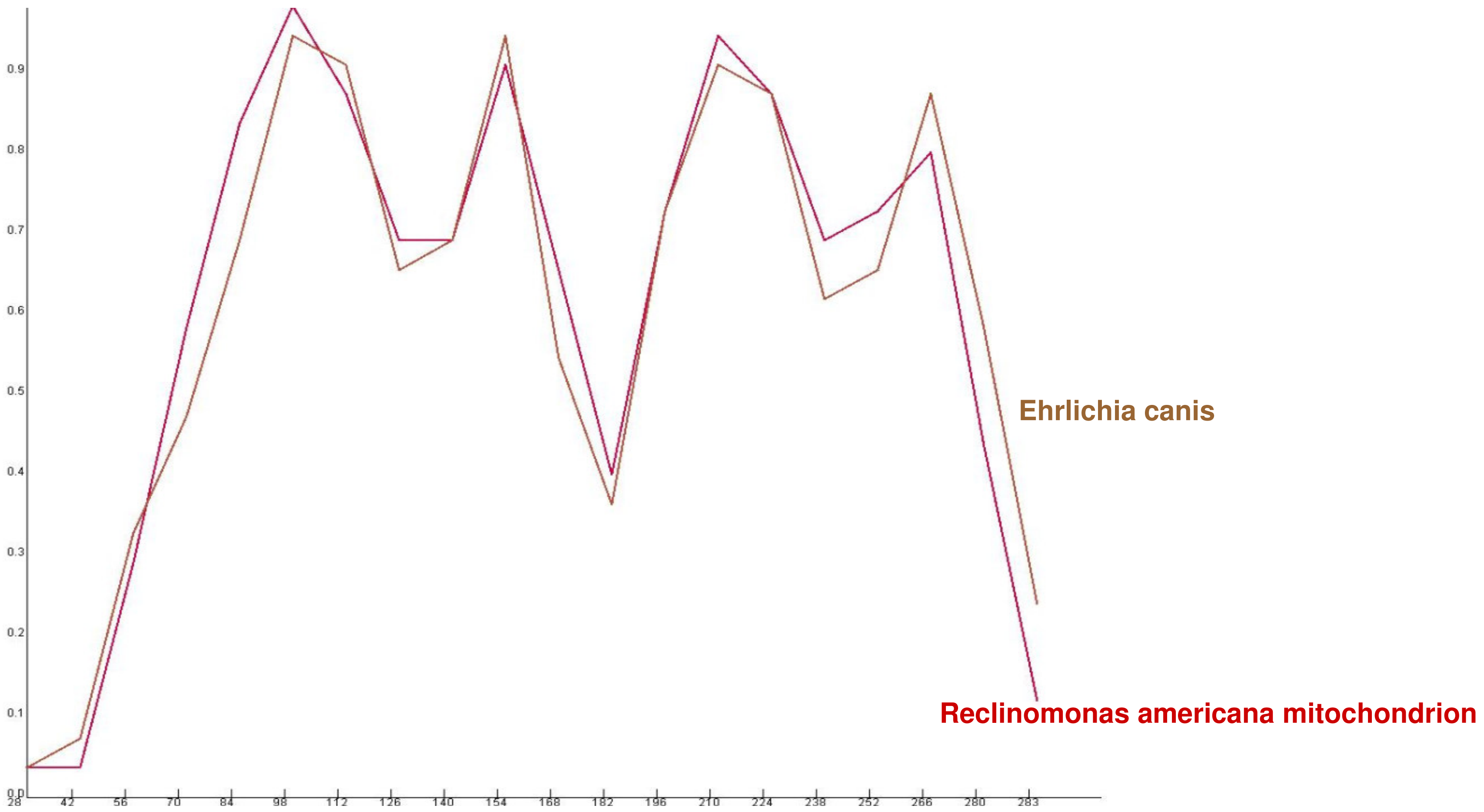

Repeat elements (*) and recombioned genes positioning:

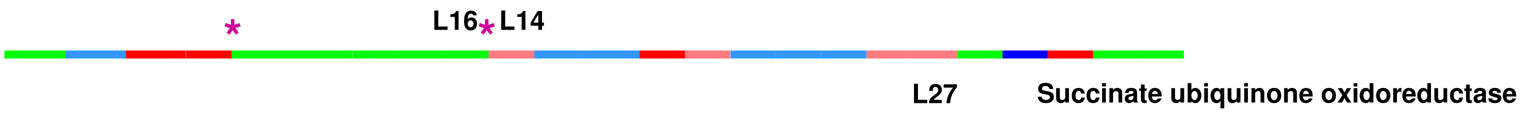

Ribosomal L22

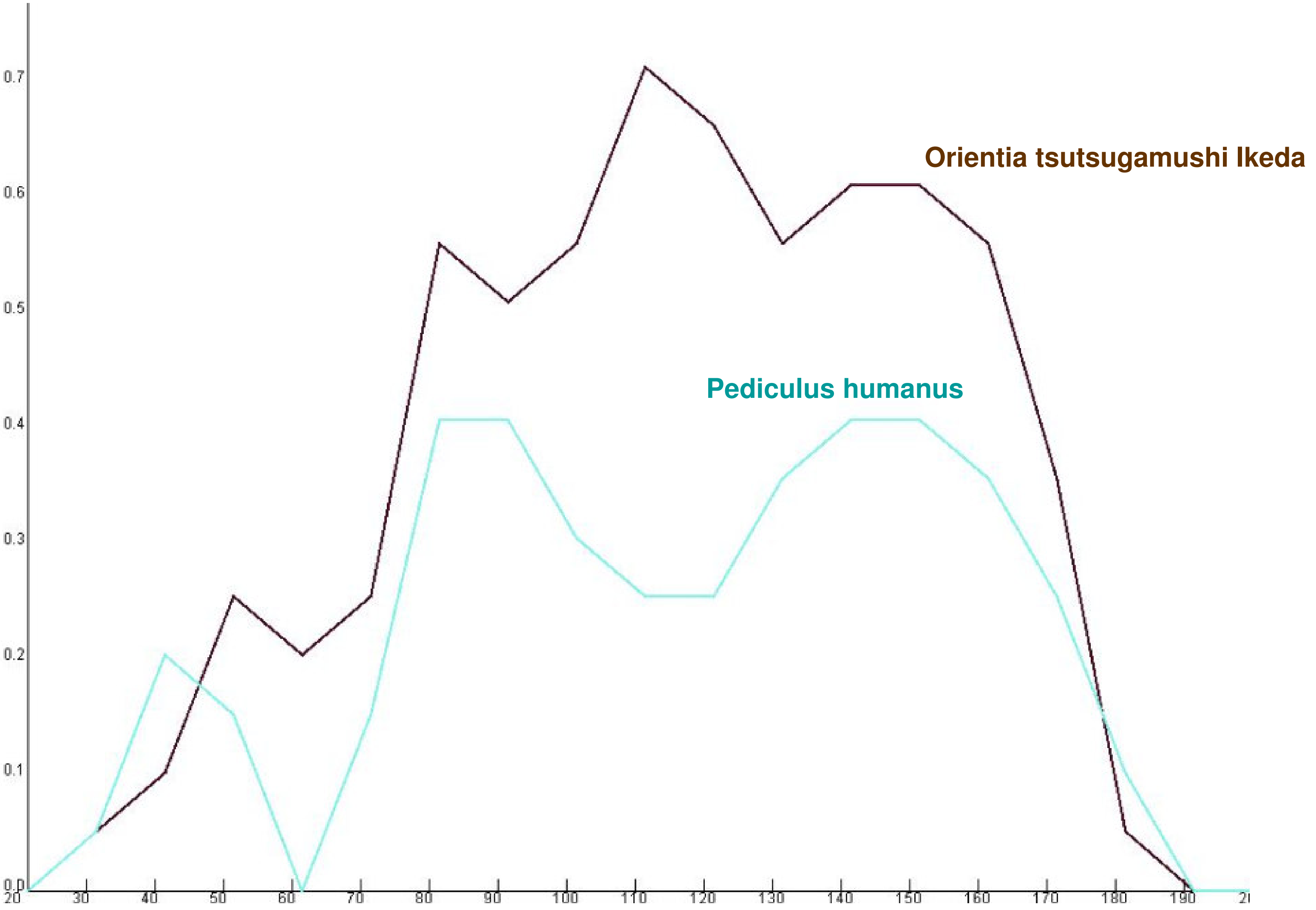

Ribosomal S2

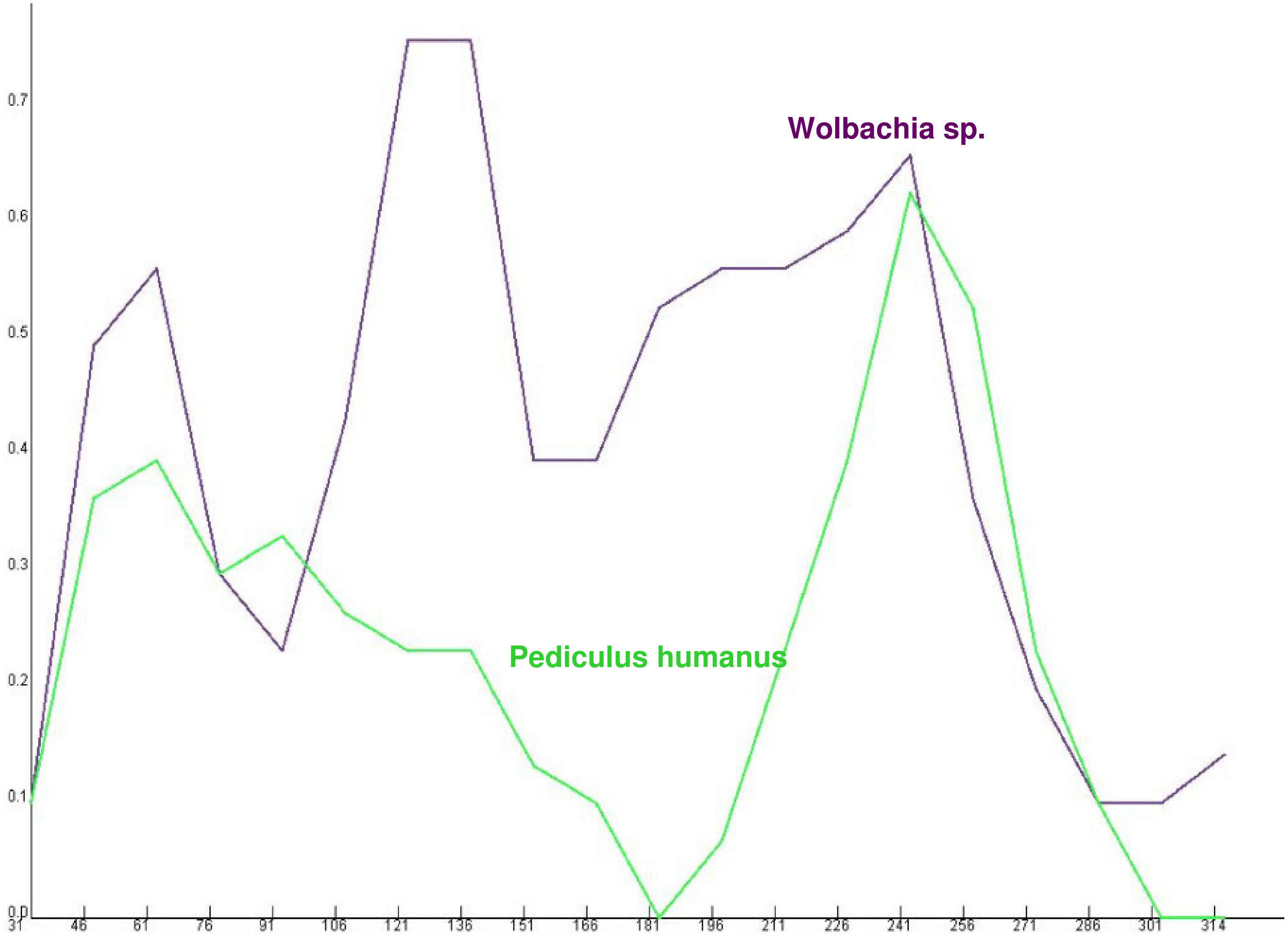

Ribosomal S16

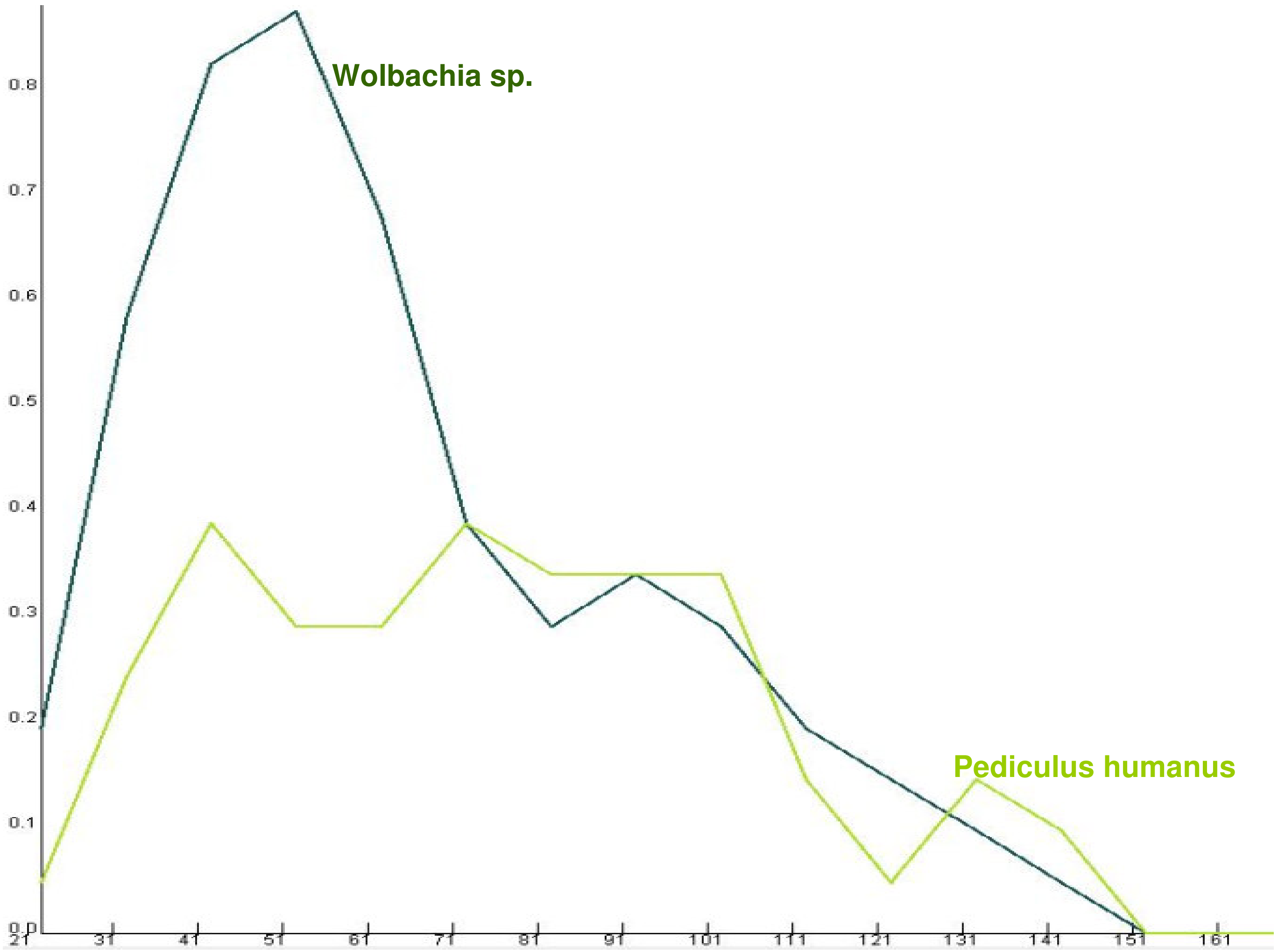

Ribosomal L13

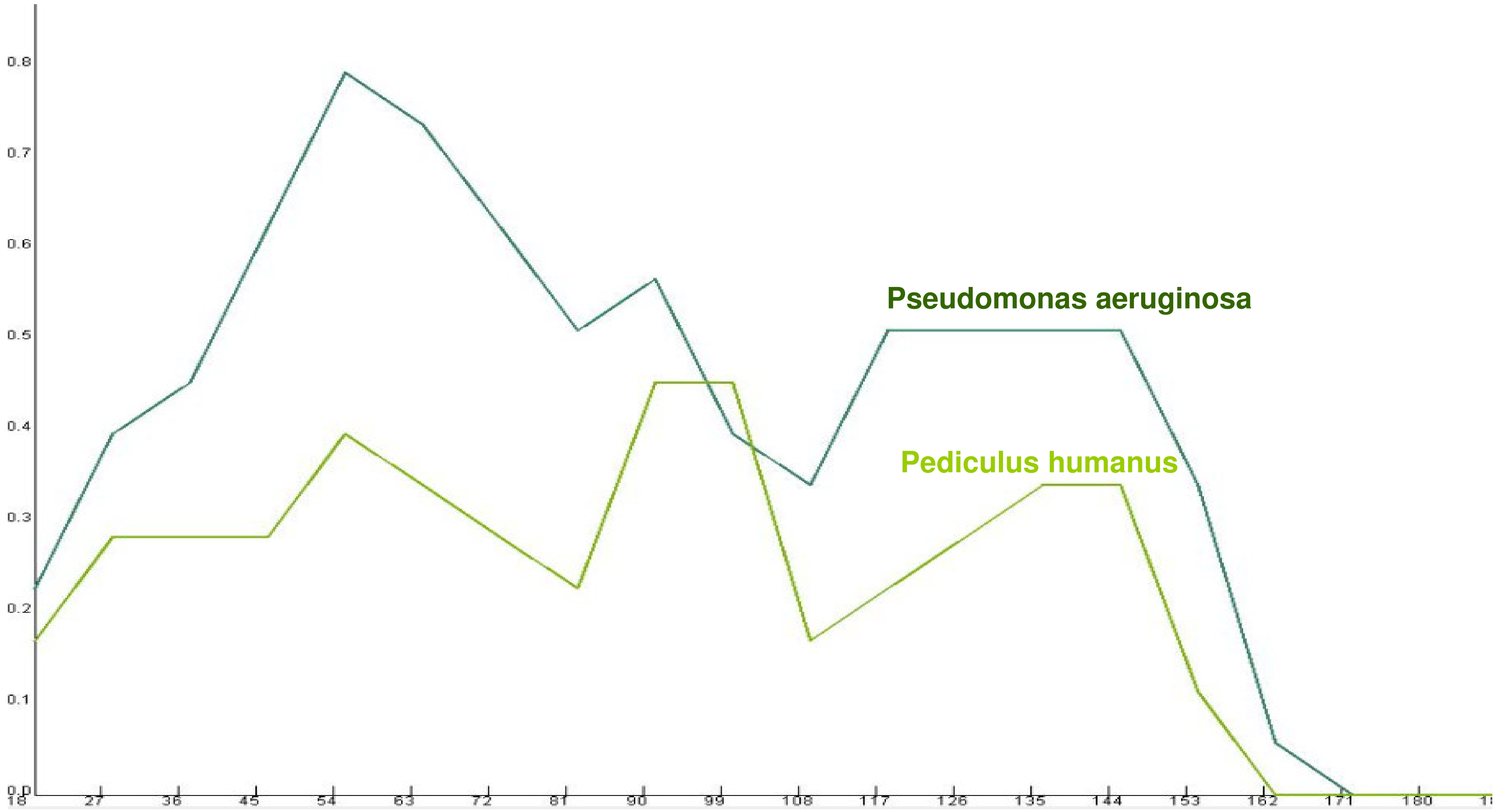

Supplement: Additional file 6 — Recombination events in the Reclinomonas americana and Pediculus humanus mitochondria. [file 1745-6150-6-55-S6.PDF]
